# Nos2 Inactivation Promotes the Development of Medulloblastoma in *Ptch1^+/−^* Mice by Deregulation of Gap43–Dependent Granule Cell Precursor Migration

**DOI:** 10.1371/journal.pgen.1002572

**Published:** 2012-03-15

**Authors:** Daniel Haag, Petra Zipper, Viola Westrich, Daniela Karra, Karin Pfleger, Grischa Toedt, Frederik Blond, Nicolas Delhomme, Meinhard Hahn, Julia Reifenberger, Guido Reifenberger, Peter Lichter

**Affiliations:** 1 Division of Molecular Genetics, German Cancer Research Center (DKFZ), Heidelberg, Germany; 2 Department of Neuropathology, Heinrich Heine University, Düsseldorf, Germany; 3 Department of Dermatology, Heinrich Heine University, Düsseldorf, Germany; Washington University School of Medicine, United States of America

## Abstract

Medulloblastoma is the most common malignant brain tumor in children. A subset of medulloblastoma originates from granule cell precursors (GCPs) of the developing cerebellum and demonstrates aberrant hedgehog signaling, typically due to inactivating mutations in the receptor PTCH1, a pathomechanism recapitulated in *Ptch1^+/−^* mice. As nitric oxide may regulate GCP proliferation and differentiation, we crossed *Ptch1^+/−^* mice with mice lacking inducible nitric oxide synthase (Nos2) to investigate a possible influence on tumorigenesis. We observed a two-fold higher medulloblastoma rate in *Ptch1^+/−^ Nos2^−/−^* mice compared to *Ptch1^+/−^ Nos2^+/+^* mice. To identify the molecular mechanisms underlying this finding, we performed gene expression profiling of medulloblastomas from both genotypes, as well as normal cerebellar tissue samples of different developmental stages and genotypes. Downregulation of hedgehog target genes was observed in postnatal cerebellum from *Ptch1^+/+^ Nos2^−/−^* mice but not from *Ptch1^+/−^ Nos2^−/−^* mice. The most consistent effect of *Nos2* deficiency was downregulation of growth-associated protein 43 (Gap43). Functional studies in neuronal progenitor cells demonstrated nitric oxide dependence of *Gap43* expression and impaired migration upon Gap43 knock-down. Both effects were confirmed *in situ* by immunofluorescence analyses on tissue sections of the developing cerebellum. Finally, the number of proliferating GCPs at the cerebellar periphery was decreased in *Ptch1^+/+^ Nos2^−/−^* mice but increased in *Ptch1^+/−^ Nos2^−/^* ^−^ mice relative to *Ptch1^+/−^ Nos2^+/+^* mice. Taken together, these results indicate that Nos2 deficiency promotes medulloblastoma development in *Ptch1^+/−^* mice through retention of proliferating GCPs in the external granular layer due to reduced Gap43 expression. This study illustrates a new role of nitric oxide signaling in cerebellar development and demonstrates that the localization of pre-neoplastic cells during morphogenesis is crucial for their malignant progression.

## Author Summary

Medulloblastoma is a common pediatric brain tumor, a subtype of which is driven by aberrant hedgehog pathway activation in cerebellar granule cell precursors. Although this tumor etiology has been intensively investigated in the well-established *Ptch1^+/−^* mouse model, knowledge is still lacking about the molecular interactions between neoplastic transformation and other developmental processes. Nitric oxide (NO) has been reported to be involved in controlling proliferation and differentiation of these cells. Therefore, inactivation of the NO–producing enzyme Nos2 in combination with the mutated *Ptch1* gene should provide insight into how developmental regulation influences pathogenesis. Here, we describe a new role for NO in developing neuronal precursors of the cerebellum facilitating physiologically accurate migration via regulation of Gap43. We further demonstrate that disturbance of these processes leads to retention of granule precursor cells to the cerebellar periphery. Together with the sustained proliferation of these cells in combined *Ptch1^+/−^ Nos2^−/−^* mice, this effect results in an increased medulloblastoma incidence relative to *Ptch1^+/−^* mice and demonstrates a new disease-promoting mechanism in this tumor entity.

## Introduction

Medulloblastoma (MB) is a highly malignant tumor of the cerebellum that preferentially develops in children and adolescents. Although the survival rate for standard risk MB is around 70% [1] surviving patients often suffer from neurodevelopmental and cognitive side effects of the aggressive therapy [2]. Therefore, improved understanding of the molecular pathomechanisms driving MB growth is necessary to develop less toxic and more effective treatments. Recent molecular profiling studies suggested at least four MB subtypes that are associated with distinct expression profiles, genomic aberrations and clinical features [3], [4]. One of these MB subtypes is characterized by aberrant activation of the hedgehog (Hh) pathway and typically corresponds to the desmoplastic (nodular) MB variant. This subtype is supposed to develop from granule cell precursors (GCPs) of the external granular layer (EGL) [5].

The EGL is a transient germinal zone at the subpial cerebellar surface consisting of rhombic lip-derived progenitor cells that have migrated tangentially to the emerging cerebellar cortex at late stages of embryonal brain development [6]. During the early postnatal period in mouse, the morphogenic factor sonic hedgehog (Shh) is secreted by subjacent Purkinje cells and binds to patched receptors (Ptch1 and Ptch2) expressed on the GCP surface [7]. Ligand binding to Ptch1 then leads to functional de-repression of Smoh (*Drosophila smoothened* homolog) and subsequent activation of Gli (Glioma-associated oncogene family zinc finger) transcription factors [8]. This launches a temporally concerted gene expression pattern causing a proliferation burst and massive expansion of the GCP population during the first two postnatal weeks [7]. In particular, the direct Gli-target *N-myc* [9], [10] and D type cyclins [11] were shown to be crucial for the growth and neoplastic transformation of GCPs [12]. In addition, the set of genes targeted by activated Gli transcription factors also include components of the canonical Hh pathway for feedback-loop regulation, such as the receptors *Ptch1* and *Ptch2* as well as the hedgehog-interacting protein (*Hip*) [10], [13]. After several rounds of cell division, GCPs normally exit cell cycle and accumulate at the inner site of the EGL [14], where they start to migrate through the molecular layer (ML) and the Purkinje cell layer to form the internal granular layer (IGL) [15]. The mechanisms underlying the attenuation of the mitotic response and eventually the stop of GCP proliferation are not well understood. The most evident concepts describe extrinsic cues in gradient-based models to trigger GCP differentiation with increasing distance to the region of the outer EGL [16]. Finally, the EGL disappears at about three weeks after birth in mice.

*PTCH1* was identified as a frequent target of inactivating mutations or genomic loss in sporadic MBs [17]–[19] that belong to the molecular subtype hallmarked by an aberrant activity of hedgehog signaling. The monoallelic inactivation of the *Ptch1* gene in mice and thus downstream activation of the Hh pathway leads to MB development at a frequency of about 10–15% [20]. This mouse model has provided substantial insights into the pathogenesis of Hh-dependent MBs and has been used in different cross-breeding experiments to investigate tumor suppressor gene functions in this particular context [21], [22].

Nitric oxide (NO) is a highly reactive gaseous molecule involved in various physiological processes ranging from vasculature modulation to neurotransmission [23], [24]. NO is produced by three distinct enzyme isoforms: *i)* neuronal nitric oxide synthase (nNos/Nos1), *ii)* inducible nitric oxide synthase (iNos/Nos2), and *iii)* endothelial nitric oxide synthase (eNos/Nos3). Though being constitutively expressed in their respective tissue, nNos and eNos activity strongly depends on calcium [25], whereas calcium-independent iNos is primarily regulated by transcriptional induction, e.g. by inflammatory cytokines and endotoxins [26], which permits higher quantities of NO generation. The role of NO in cancer initiation and progression is heterogeneous with opposing effects in different malignancies [27]. Considering effects of tumor stroma, increased angiogenesis was reported to be associated with elevated Nos activity [28] and some immune-related processes were found to be mediated by NO [29], including cytotoxicity of activated microglia [30]. Finally, NO released by vascular endothelial cells was reported to build a niche-like microenvironment for maintenance of glioma stem cells [31]. In the context of cerebellar development, Nos2 (inducible Nos) is initially expressed in early GCPs, whereas Nos1 (neuronal Nos) is hardly present before postnatal day 7 (Cerebellar Development Transcriptome Database [32]). Successively, Nos1 expression increases along with granule cell differentiation [33] and predominantly contributes to the common NO signaling that becomes apparent in the IGL as development proceeds [34]. Evidence has been provided that NO negatively acts on proliferation of neuronal precursors during adult neurogenesis [35]. Similarly, Ciani and colleagues demonstrated enhanced proliferation of cerebellar precursor cells upon inhibition of NO synthases [36].

Here, we report on the generation of *Ptch1^+/−^ Nos2^−/−^* mice to investigate the impact of Nos2 on tumor development in *Ptch1* hemizygous mutant mice. Interestingly, we observed an approximately two-fold increase in the incidence of spontaneous MB in *Ptch1^+/−^ Nos2^−/−^* mice in comparison to *Ptch1^+/−^ Nos2^+/+^* mice. To characterize the molecular pathomechanism underlying the tumor-promoting effect of Nos2 deficiency in *Ptch1^+/−^* mice, we performed comprehensive expression and DNA copy number profiling of MB tumors (*Ptch1^+/−^ Nos2^+/+^ versus Ptch1^+/−^ Nos2^−/−^*) as well as expression profiling of normal cerebellar tissue samples from different developmental stages and various genotypes (*Ptch1^+/−^ Nos2^+/+^*, *Ptch1^+/−^ Nos2^−/−^*, *Ptch1^+/+^ Nos2^−/−^* and wild-type mice). Downregulation of the growth-associated protein 43 (*Gap43*) was the most striking feature in the cerebellum of *Nos2*-deficient mice when compared to *Ptch1^+/−^ Nos2^+/+^* and wild-type mice. Subsequent functional analyses and results from *in situ* studies of GCPs in postnatal cerebellum allowed us to formulate a model for the tumor promoting role of *Nos2* deficiency in *Ptch1* mutant mice via deregulation of Gap43-dependent migration of GCPs.

## Results

### Loss of *Nos2* increases the rate of spontaneous MB in *Ptch1^+/−^* mice

**Figure 1:**
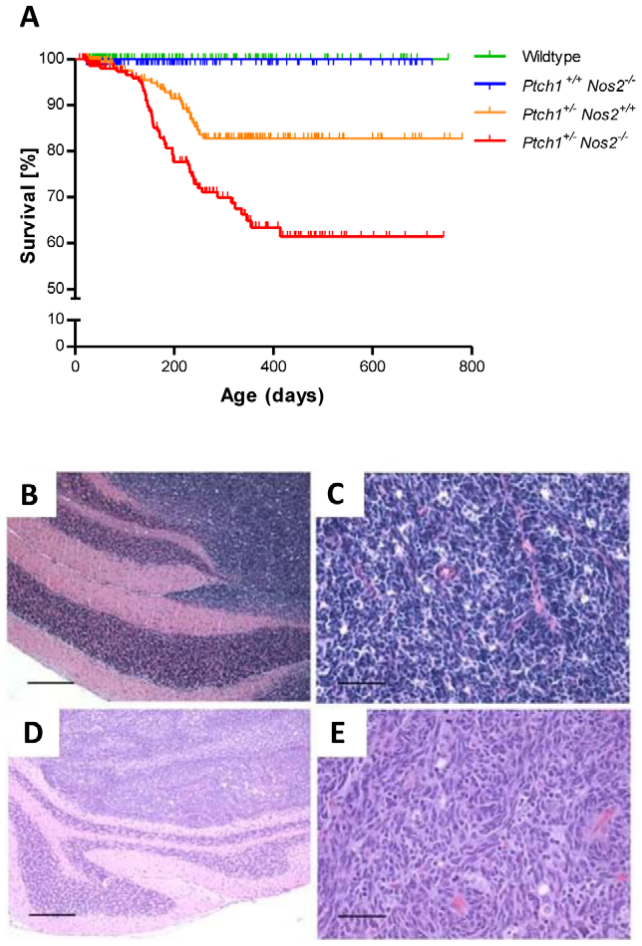
*Nos2* deficiency increases the incidence of MB in *Ptch1* ^+/−^ mice. (A) Kaplan-Meier analysis of MB incidence in 215 *Ptch1* ^+/−^ *Nos2* ^+/+^ mice (orange line) versus 221 *Ptch1* ^+/−^ *Nos2* ^−/−^ mice (red line). *Ptch1* ^+/−^ *Nos2* ^−/−^ mice demonstrated an approximately two-fold increase in MB incidence (p = 0.0007, Logrank test). None of 315 wild-type (green line) and 412 *Ptch1* ^+/+^ *Nos2* ^−/−^mice (blue line) (control littermates) developed any MB. Vertical ticks represent censored mice. (B–E) Histological features of MBs from *Ptch1* ^+/−^ *Nos2* ^+/+^ and *Ptch1* ^+/−^ *Nos2* ^−/−^ mice. MBs in both genotypes (B–C, *Ptch1* ^+/−^ *Nos2* **^+/+^**; D–E, *Ptch1* ^+/−^ *Nos2* ^−/−^) were densely cellular primitive neuroectodermal tumors of the cerebellum corresponding histologically to the classic subtype of human MB. Hematoxylin eosin-stained sections showed no obvious differences between the genotypes concerning gross growth pattern, with well demarcated growth in the cerebellar cortex (B, D, scale bar = 500 µm), and cellular morphology (C, E, scale bar = 50 µm).

Survival analyses of 315 wild-type mice, 412 *Ptch1^+/+^ Nos2^−/−^* mice, 215 *Ptch1^+/−^ Nos2^+/+^* mice and 221 *Ptch1^+/−^ Nos2^−/−^* mice demonstrated a significantly higher MB incidence in the group of *Ptch1^+/−^ Nos2^−/−^* mice relative to the group of *Ptch1^+/−^ Nos2^+/+^* mice (p = 0.0007, Logrank test, Figure 1A). In total, 11% of the *Ptch1^+/−^ Nos2^+/+^* mice (24/215) and 21% of the *Ptch1^+/−^ Nos2^−/−^* mice (47/221) were sacrificed due to the development of cerebellar MB. None of the 315 wild-type and the 412 *Ptch1^+/+^ Nos2^−/−^* mice developed MBs. These observations indicate a MB-promoting role of *Nos2* deficiency in *Ptch1* ^+/−^ mice.

### MBs in *Ptch1^+/−^ Nos2^+/+^* and *Ptch1^+/−^ Nos2^−/−^* mice show identical histological features

In humans, Hh-dependent MBs typically correspond to the desmoplastic subtype. MBs in *Ptch1^+/−^* mice, however, microscopically resemble the classic MB subtype [20]. Histological analysis of MBs in *Ptch1^+/−^ Nos2^+/+^* and *Ptch1^+/−^ Nos2^−/−^* mice demonstrated similar morphological features (Figure 1B–1E). The tumors were composed of densely packed sheets of cells with hyperchromatic carrot-shaped nuclei and scant cytoplasm. There were no obvious histopathological differences between MBs of the two genotypes.

### Molecular analyses of MBs in *Ptch1^+/−^ Nos2^+/+^* and *Ptch1^+/−^ Nos2^−/−^* mice

For an initial assessment of the molecular tumor characteristics, gene expression of hedgehog signaling pathway components were measured in 21 MBs and 24 normal (adult) cerebellar tissue samples from both *Ptch1^+/−^ Nos2^+/+^* and *Ptch1^+/−^ Nos2^−/−^* mice. Using quantitative real-time PCR (qRT-PCR), significant downregulation of the wild-type *Ptch1* transcript and upregulation of the Shh target genes *Gli1* and *N-myc* were generally observed in the tumor samples (Figure S1), indicating all examined MBs to be of the same Hh-dependent molecular subtype. However, there were no significant differences for these genes between MBs of the two genotypes. Furthermore, targeted genetic analyses showed a loss of the wild-type *Ptch1* allele in 10 of the 21 MBs investigated, while none of the tumors demonstrated a *Tp53* mutation or *N-myc* amplification. The *Cdkn2a/p16^INK4a^* locus was retained in all tumors while a single MB demonstrated a homozygous *p19^ARF^* deletion (see Table S1 and Text S1 for details).

In order to identify the molecular pathomechanism contributing to the increased MB rate in *Nos2*-deficient *Ptch1* mutant mice, we performed array-based gene expression profiling of three *Ptch1^+/−^ Nos2^+/+^* versus six *Ptch1^+/−^ Nos2^−/−^* and comparative genomic hybridization (array-CGH) analyses of five *Ptch1^+/−^ Nos2^+/+^* versus seven *Ptch1^+/−^ Nos2^−/−^* MB tissue samples. All specimens investigated had tumor cell contents between 70% and 90% as determined on corresponding formalin-fixed and paraffin-embedded (FFPE) reference sections. Differential expression of selected candidate genes was validated by qRT-PCR on an expanded, partially overlapping tumor set of seven *Ptch1^+/−^ Nos2^+/+^* versus seven *Ptch1^+/−^ Nos2^−/−^* MB samples.

The expression profiling analysis revealed a total of 87 differentially regulated genes between tumors of the two genotypes (Table S2) with the vast majority (87%) showing lower transcript levels in *Ptch1^+/−^ Nos2^−/−^* when compared to *Ptch1^+/−^ Nos2^+/+^* mice. As expected from the initial targeted qRT-PCR measurements, there was no difference detectable concerning the activation of Hh pathway genes. Due to the important role of *Nos2* during angiogenesis and cancer-associated immune response, including microglia, stromal effects need to be particularly considered in a systemic *Nos2* knockout model. However, neither the set of significantly deregulated genes nor selective determination of marker expression for pericytes, vascular endothelial cells or microglia suggested any differences in the tumor stroma between the two genotypes (see Table S3 and Text S1 for details). According to the findings of Ciani and co-workers [37], reduction of NO enhances GCP proliferation through an increased expression of the proto-oncogene N-myc. Therefore, protein levels were particularly examined for differences between tumor samples from *Ptch1^+/−^ Nos2^+/+^* and *Ptch1^+/−^ Nos2^−/−^* mice. The results shown in Figure S2, however, revealed similar expression of N-myc in all MBs.

**Figure 2:**
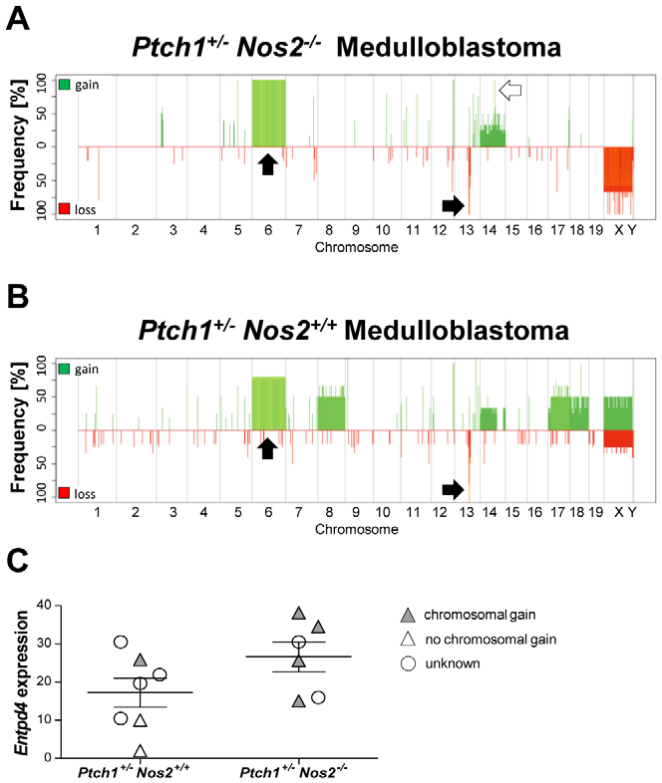
Microarray-based CGH analysis of MB samples. (A–B) Alteration frequency plots of *Ptch1^+/−^ Nos2^−/−^* and *Ptch1^+/−^ Nos2^+/+^* tumors show a common trisomy of chromosome 6 and deleted regions around the *Ptch1* locus (chromosome 13), green: gains, red: losses. Arrows denote frequent aberrations, black: common, white: differential. Color shading indicates percentage of available signals for a respective oligonucelotide (% of samples), light: high percentage, dark: low percentage. Concurrent gains and losses of X and Y chromosomes, or *vice versa*, reflect sample against reference hybridizations of different gender. The most consistent difference between MBs of both genotypes affected a small region on chromosome 14 harboring the *Entpd4* gene. (C) QRT-PCR of *Entpd4* showed no significant difference in an expanded set of tumor samples. Relative expression values are normalized to housekeeping genes. Error bars reflect SEM (standard error of the mean).

Analyses of genomic copy number alterations revealed a trisomy of chromosome 6 in the majority of MBs from both groups (11/12, Figure 2A and 2B). Moreover, a small region on chromosome 13, approximately 1.5 Mb upstream of the *Ptch1* gene, showed a hemizygous deletion in healthy cerebella of *Ptch1*-mutant mice (data not shown) but a homozygous deletion in most tumors (10/12). Similarly, a second small region 3.8 Mb downstream of the last *Ptch1* exon exhibited a loss in 9 of 12 MBs. This suggests structural changes flanking the *Ptch1* locus that likely contribute to inactivation of the wild-type allele. The frequencies of genomic aberrations showed a more heterogeneous karyotype with gross structural changes in *Ptch1^+/−^ Nos2^+/+^* MBs when compared to *Ptch1^+/−^ Nos2^−/−^* MBs (see Figure 2A, 2B and Text S1 for details). However, a general difference in chromosomal instability was not obvious between both genotypes. Only a small region (205.6 kb) on chromosome 14 containing the *Entpd4* (ectonucleoside triphosphate diphosphohydrolase 4) gene was more frequently gained in *Ptch1^+/−^ Nos2^−/−^* MBs (7/7) than in *Ptch1^+/−^ Nos2^+/+^* MBs (1/5, Figure 2A). Accordingly, *Entpd4* expression appeared to be specifically upregulated in expression profiles of *Ptch1^+/−^ Nos2^−/−^* tumors. QRT-PCR validation confirmed an elevated mean expression in *Ptch1^+/−^ Nos2^−/−^* compared to *Ptch1^+/−^ Nos2^+/+^* MBs in those samples that overlapped with the array-CGH analysis but revealed no significant difference across the expanded tumor set (Figure 2C).

### *Nos2* inactivation is associated with low expression of mitotic genes in postnatal cerebellum

As GCPs are considered the cells of origin for the Hh-dependent MB subtype, we examined the effect of *Nos2* ablation in the context of cerebellar development. Therefore, gene expression profiles of normal cerebellar tissue samples from postnatal day 9 (P9), 6 weeks after birth (6W), and 1 year of age (1Y) were generated from wild-type, *Ptch1^+/−^ Nos2^+/+^*, *Ptch1^+/−^ Nos2^−/−^*, and *Ptch1^+/+^ Nos2^−/−^* animals. While specimens of mature cerebellum (6W and 1Y) were investigated separately in 3 biological replicates per genotype and developmental stage, samples of postnatal cerebellum consisted of pooled RNA from 4–5 individuals processed in technical replicates due to limited tissue amounts.

**Figure 3:**
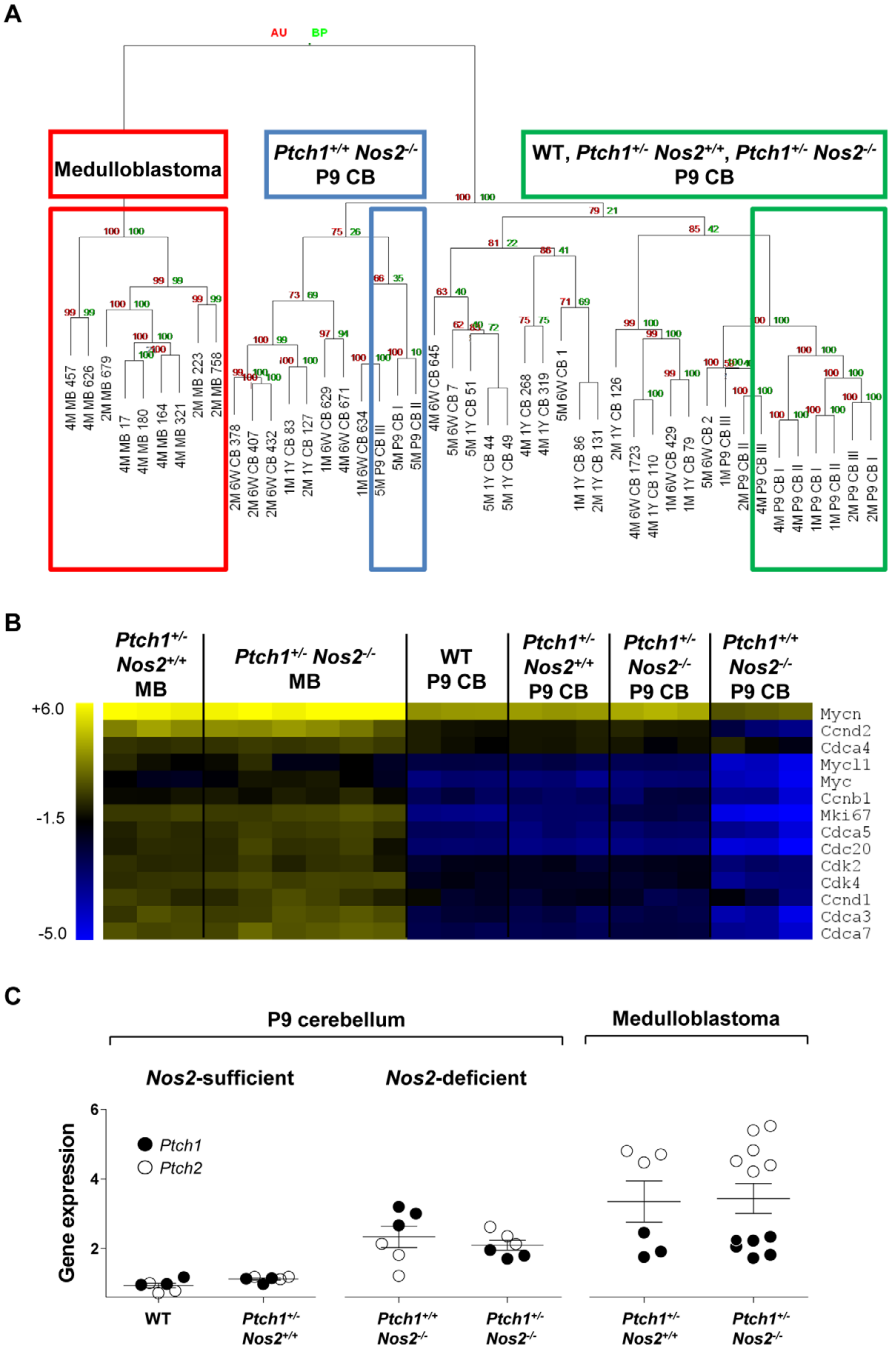
Microarray-based gene expression profiling. (A) Hierarchical cluster analysis of normal cerebellar tissue and MB samples showed a clear separation of *Ptch1^+/+^ Nos2^−/−^* P9 cerebella. (B) Heat map of proliferation-associated genes downregulated in P9 cerebella of *Ptch1^+/+^ Nos2^−/−^* versus wild-type mice. Depicted samples include postnatal cerebellar tissue samples of each genotype and all MB cases. Values are normalized to gene-wise average for better visualization. (C) Gene expression of *Ptch1* was increased in P9 cerebellar tissue samples of *Nos2*-deficient mice. *Ptch2* was increased over *Ptch1* expression only in *Ptch1^+/−^ Nos2^−/−^* P9 cerebella and MBs. Values are indicated as log2 ratios of sample against Universal Reference RNA (Stratagene). Error bars reflect SEM. AU: approximately unbiased p-values by multiple bootstrap resampling [%], BP: boostrap probability p-values by normal bootstrap resampling [%]. MB: medulloblastoma, CB: cerebellum, P9: postnatal day 9, 6W: 6 weeks after birth, 1Y: 1 year after birth.

An unsupervised hierarchical cluster analysis of transcriptome data clearly separated developing cerebellum (P9) of wild-type mice and the two *Ptch1*-mutated genotypes from mature cerebellum. Interestingly, P9 cerebellum of *Ptch1^+/+^ Nos2^−/−^* mice displayed different properties highly similar to mature cerebellum (Figure 3A). Expression profiles of MBs formed a distinct cluster clearly separated from all healthy tissue samples.

A direct comparison between gene expression profiles from *Ptch1^+/+^ Nos2^−/−^* and wild-type P9 cerebellar tissue samples resulted in a total of 984 deregulated genes with 755 genes (76.7%) showing a decreased expression in *Ptch1^+/+^ Nos2^−/−^* mice (Table S4). P9 cerebellum from *Ptch1^+/−^ Nos2^+/+^* and *Ptch1^+/−^ Nos2^−/−^* mice revealed only 5 and 32 deregulated genes relative to wild-type, respectively (Table S5 and Table S6). This large deviation of postnatal gene expression in the *Ptch1^+/+^ Nos2^−/−^* genotype included a set of downregulated genes that are essential for proliferation of GCPs (e.g. cyclin D1, cyclin D2 and *N-myc*, Figure 3B). As hedgehog signaling constitutes the main regulatory pathway for neonatal cell proliferation in GCPs of the EGL, the 984 deregulated genes were analyzed for enrichment of Gli transcription factor targets. Matching this list to a set of recently identified Gli-targets in GCPs [10] yielded a significant overrepresentation of Gli1-regulated genes (p = 0.005, chi-square test). Hence, the reduced transcript levels of these target genes suggests an attenuated hedgehog signaling in postnatal *Ptch1^+/+^ Nos2^−/−^* cerebellum compared to wild-type (or any other genotype).

Notably, the decreased expression of Gli1-targets and proliferation-associated genes observed in *Nos2*-deficient cerebellar tissue was abolished upon additional inactivation of the hedgehog receptor *Ptch1* (in *Ptch1^+/−^ Nos2^−/−^* mice). Therefore, we examined the transcript levels of patched receptors themselves in more detail. While neither *Ptch1* nor *Ptch2* expression was changed between wild-type and *Ptch1^+/−^ Nos2^+/+^* P9 cerebellum, a significant increase of *Ptch1* and a minor increase of *Ptch2* expression were observed in *Ptch1^+/+^ Nos2^−/−^* mice relative to wild-type mice (Figure 3C). Notably, in *Ptch1^+/−^ Nos2^−/−^* cerebellar tissue samples, *Ptch2* expression was more elevated than *Ptch1*. However, since Ptch2 is not capable of inhibiting *smoothened* (Smoh), it probably failed to take over the attenuating effect on Gli activity [38]. MB specimens from *Ptch1^+/−^ Nos2^+/+^* versus *Ptch1^+/−^ Nos2^−/−^* mice showed no significant difference in expression levels of either patched receptor, with *Ptch2* being substantially increased over *Ptch1* in both groups (Figure 3C). These findings indicate that *Nos2* deficiency leads to an upregulation of *Ptch1* in GCPs, which results in a downregulation of mitotic genes and Gli-targets only in a *Ptch1*-wild-type background.

### Decreased expression of *Gap43* is the most common effect of *Nos2* inactivation

**Figure 4:**
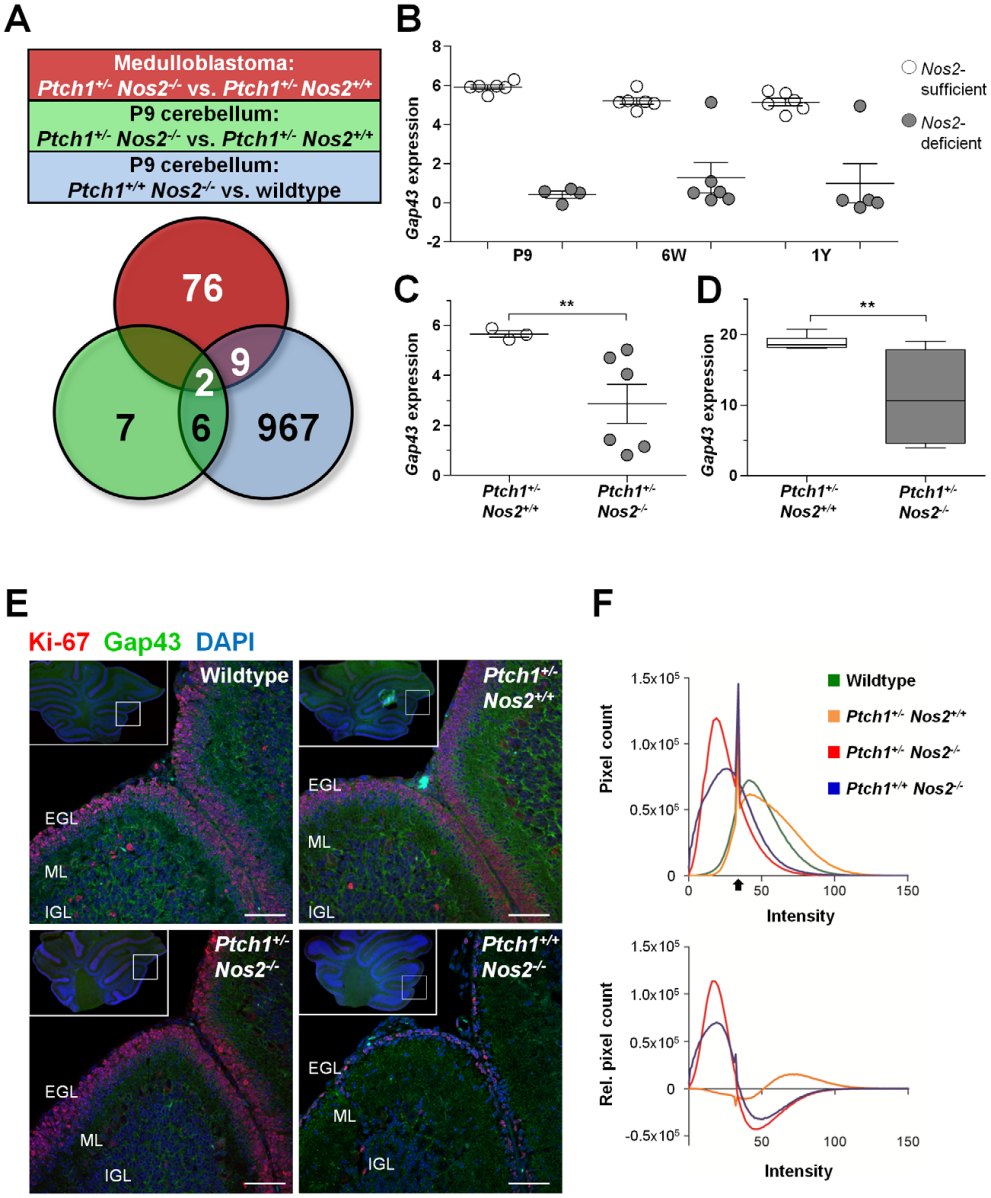
Identification of *Nos2*-regulated candidate genes. (A) The overlap between three group comparisons of expression profiles revealed *Gap43* and *Stmn1* as commonly deregulated by *Nos2* inactivation according to the microarray data. (B) *Gap43* gene expression was *Nos2*-dependent during different developmental stages of cerebellar development. *Nos2*-sufficient: wild-type and *Ptch1^+/−^ Nos2^+/+^*, *Nos2*-deficient: *Ptch1^+/+^ Nos2^−/−^* and *Ptch1^+/−^ Nos2^−/−^*. (C) *Gap43* was differentially expressed between *Ptch1^+/−^ Nos2^+/+^* and *Ptch1^+/−^ Nos2^−/−^* MB samples. Values in (B) and (C) were obtained from the microarray data and indicate log2 ratios of sample against Universal Reference RNA (Stratagene). (D) Differential *Gap43* gene expression was confirmed by qRT-PCR in an expanded tumor sample set (n = 7 per genotype). Linear expression values are normalized to housekeeping genes. Significant expression differences between groups are indicated by asterisks (**p<0.01). Error bars reflect SEM. (E) Immunofluorescent co-staining of Gap43 (green) and Ki-67 (red) on FFPE sections from P9 cerebella. Blue: DAPI-stained nuclei. Overview sections (upper left corner) were acquired by wide-field microscopy and detail sections represent confocal laser scanning microscopy images. Intense Gap43 signal was observed in wild-type and *Ptch1^+/−^ Nos2^+/+^* cerebella, in particular at the outer ML, scale bar = 50 µM. (F) Quantification of Gap43 staining showing intensity histograms of the EGL and ML area (upper panel), as well as intensity distribution of the different genotypes with subtracted wild-type signal (lower panel). Black arrow indicates a common peak of nucleus background signal. EGL: external granule layer, ML: molecular layer, and IGL: internal granule layer.

So far, *Nos2* inactivation was shown to counteract proliferation and antagonize hedgehog signaling in developing cerebella. To identify those *Nos2*-dependent effects promoting MB induction, we determined the features that were common to *Ptch1^+/+^ Nos2^−/−^* and *Ptch1^+/−^ Nos2^−/−^* genotypes and persisted in the tumor tissues. Accordingly, the overlap of differential gene expression from three comparisons was built: *i) Ptch1^+/+^ Nos2^−/−^* versus wild-type P9 cerebellum, *ii) Ptch1^+/−^ Nos2^−/−^* versus *Ptch1^+/−^ Nos2^+/+^* P9 cerebellum; and *iii) Ptch1^+/−^ Nos2^−/−^* versus *Ptch1^+/−^ Nos2^+/+^* MB. As a result, only 2 genes were observed to be deregulated in a *Nos2*-dependent manner during cerebellar development and in MBs (Figure 4A). Although *Stmn1* (stathmin 1) appeared to be upregulated in *Ptch1^+/−^ Nos2^−/−^* MBs relative to *Ptch1^+/−^ Nos2^+/+^* MBs, this could not be confirmed by qRT-PCR (Figure S3). Gene expression of *Gap43* was consistently reduced in *Nos2*-deficient cerebellar tissue samples and downregulation in *Ptch1^+/−^ Nos2^−/−^* tumors relative to *Ptch1^+/−^ Nos2^+/+^* tumors was also significant in the expanded validation set (Figure 4C and 4D). To further assess the immediacy of *Nos2* inactivation and *Gap43* deregulation, *Gap43* transcript levels were determined in expression profiles of healthy cerebella from all developmental stages (P9, 6W and 1Y). Groups for comparison were built according to presence or absence of *Nos2*, irrespective of the *Ptch1* status. The results clearly demonstrated a close association of altered *Gap43* transcript levels and *Nos2* status (Figure 4B), and indicated downregulation of *Gap43* to be the most common effect of *Nos2* deficiency in the cerebellum.

To investigate differences in Gap43 expression on protein level *in situ* we performed immunofluorescent double stainings of Gap43 and the proliferation marker Ki-67 on FFPE sections of P9 cerebella from wild-type, *Ptch1^+/−^ Nos2^+/+^*, *Ptch^+/+^ Nos2^−/−^* and *Ptch1^+/−^ Nos2^−/−^* mice. As illustrated in Figure 4E, Gap43 immunofluorescence was particularly prominent in the outer region of the molecular layer (ML) that is connected to and partially comprised of radial GCP process extensions. Image quantification further indicate a quantitative difference of Gap43 expression in this region with sections from wild-type and *Ptch1^+/−^ Nos2^+/+^* mice showing a more intense staining than sections from *Ptch1^+/−^ Nos2^−/−^* and *Ptch1^+/+^ Nos2^−/−^* mice (Figure 4F).

### Impaired NO signaling reduces *Gap43* transcript levels

**Figure 5:**
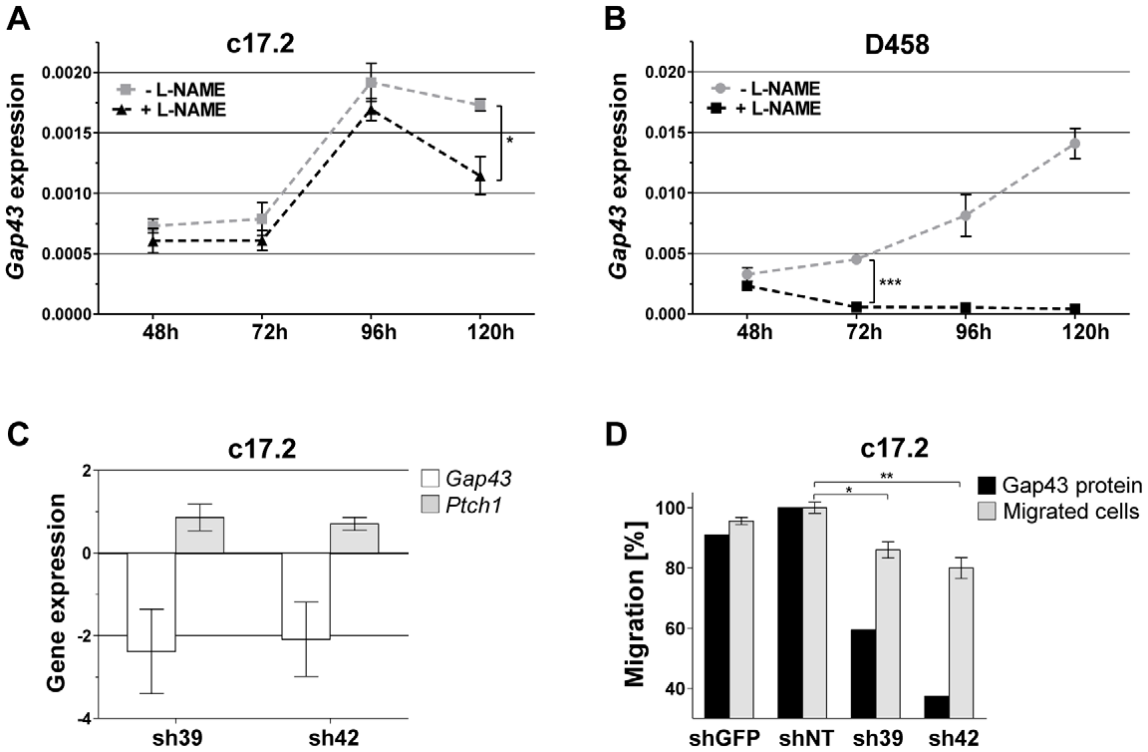
Characteristics and functional implication of Gap43 expression in cell culture. (A–B) Gene expression of *Gap43* was reduced upon inhibition of NO synthases. Expression values were obtained from qRT-PCR measurements and indicate linear expression values normalized to a pool of housekeeping genes. Error bars reflect SEM of three replicates. (A) In c17.2 cells, *Gap43* expression is significantly decreased upon L-NAME treatment after 120 hours (*p = 0.0234). (B) In D458 cells, *Gap43* expression is significantly decreased upon L-NAME treatment after 72 hours (***p<0.0001). (C–D) Functional analyses were performed after knockdown of *Gap43* in neuronal progenitor cells (c17.2). (C) *Gap43* and *Ptch1* showed inverse gene expression behavior measured by qRT-PCR and normalized to non-target control. (D) Cells exhibited reduced migration upon decrease of Gap43 protein levels. The percentage of migrated cells was normalized to non-target control. Significant decrease of migration in knockdown samples is indicated by asterisks (*p = 0.013, **p = 0.007). Sh39, sh42: anti-*Gap43* target shRNA, shGFP: control shRNA against GFP, shNT: non-target control shRNA.

The association of *Nos2* inactivation and decreased *Gap43* expression suggests a gene-regulatory function of NO signaling. In order to investigate this possible link *in vitro*, we used the murine cerebellar precursor cell line c17.2 and the human MB cell line D458 (see Text S2 for details). Both cell lines were treated either with the Nos inhibitor L-NAME (Nω-nitro-L-arginine methyl ester) to reduce NO levels or solvent control (Figure S4). Relative expression of *Gap43* was assessed every 24 hours by qRT-PCR. In c17.2 cells, *Gap43* transcript abundance was generally low and increased with culture duration. We observed a slightly decreased expression of *Gap43* upon L-NAME treatment that reached significance (p = 0.023) after 120 hours (Figure 5A). NOS inhibition in D458 human MB cells resulted in a significant reduction of *Gap43* transcript levels starting already after 72 hours with further decrease after 96 hours and 120 hours (Figure 5B). FACS analyses of apoptosis and cell cycle excluded these observations to be attributed to secondary effects of changing cell conditions (Figure S5). These results suggest *Gap43* downregulation as a direct consequence of reduced NO levels in murine neuronal precursors and human MB cells.

### Increase of *Ptch1* expression and impairment of GCP migration upon knockdown of *Gap43*

The dependency of *Gap43* expression on NO signaling suggests this gene as key mediator of the effects observed in *Nos2*-deficient P9 cerebellum and *Ptch1^+/−^ Nos2^−/−^* MB, in particular, the upregulation of functional *Ptch1* in *Ptch1^+/+^ Nos2^−/−^* mice. Mishra *et al.* recently reported a central role of Gap43 in the polarization of developing GCPs by regulating centrosome positioning and thus defining correct orientation towards the IGL [39]. Since this is a prerequisite for directed migration, reduced levels of Gap43 in P9 cerebellar tissue may lead to retention of GCPs in the EGL. To test these hypotheses, shRNA-mediated knockdown of *Gap43* was performed in c17.2 cells (see Text S1 for details). Upon knockdown of Gap43 we observed a strong inverse behavior of *Ptch1* and *Gap43* transcript levels (Figure 5C). Changes in migration characteristics were assayed in a Boyden chamber using recombinant SDF-1α (CXCL12) as chemoattractant, which was reported to participate in guiding migration of embryonal GCPs *in vivo* [40]. Downregulation of Gap43 yielded a significant decrease in cell migration between 14% (p = 0.013) and 20% (p = 0.007) (Figure 5D; Figure S6C). A pseudo-effect of the knockdown due to altered proliferation of the *v-myc*-immortalized c17.2 cells was excluded by FACS-based cell cycle analysis (Figure S7).

### GCPs of the external granular layer show Gap43–associated phenotypes

**Figure 6:**
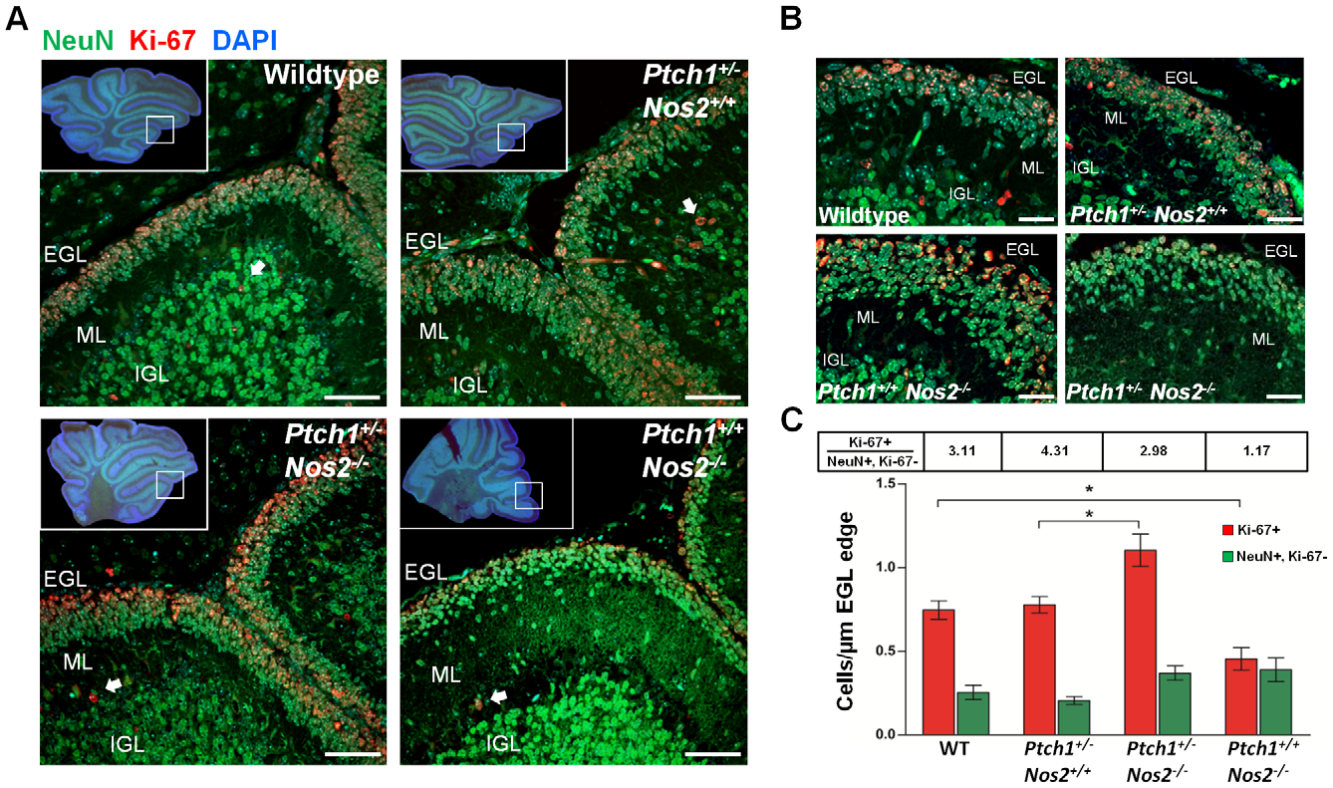
Proliferation and accumulation of GCPs in postnatal cerebellum. (A) Immunofluorescent co-staining of NeuN (green) and Ki-67 (red) on FFPE sections from P9 cerebella showed an increased accumulation of proliferating GCP in the EGL of *Ptch1^+/−^ Nos2^−/−^* mice. Overview sections were acquired by wide-field microscopy and detail images by confocal laser scanning microscopy. Blue: DAPI-stained nuclei. White arrows denote proliferating granule cells in the IGL. (B) High magnification images of the EGL and ML displayed altered morphologies especially in *Ptch1^+/−^ Nos2^−/−^* and *Ptch1^+/+^ Nos2^−/−^* mice. (C) Cell counts from immunofluorescence images. Numbers of proliferating (Ki-67+) and non-proliferating (Ki67−) cells normalized to the length of the EGL edge show significant enrichment of dividing cells in *Ptch1^+/−^ Nos2^−/−^* mice and correspondingly low numbers for *Ptch1^+/+^ Nos2^−/−^* mice. Ratios of dividing to non-dividing cells (Ki-67+, Ki-67− NeuN+) are indicated for each genotype. Significant differences are indicated by asterisks (*p<0.05). Scale bars = 50 µM. EGL: external granule layer, ML: molecular layer, IGL: internal granule layer.

Transcriptome and functional analyses suggest that a decreased *Gap43* expression accounts for *Ptch1* upregulation and impairment of directed neuronal precursor migration *in vitro*. Accordingly, *Ptch1^+/+^ Nos2^−/−^* P9 cerebella are supposed to increasingly retain GCPs with reduced mitotic activity in the EGL compared to wild-type and *Ptch1^+/−^ Nos2^+/+^* mice. Moreover, the *Ptch1^+/−^ Nos2^−/−^* genotype is also expected to exhibit retention of GCPs, but not to show any cell cycle arrest. To further verify this hypothesis *in situ* we performed immunofluorescent double staining of proliferating (Ki-67+) and post-mitotic GCPs on FFPE sections of postnatal cerebellum (Figure 6A). Here, post-mitotic cells were delineated by the neuronal marker NeuN (neuronal nuclear antigen A60) [41]. At least three different regions of each mouse cerebellum were analyzed in three to four animals per genotype using confocal laser scanning microscopy. In accordance with the microarray data, averaged cell counts of wild-type and *Ptch1^+/−^ Nos2^+/+^* mice did not show significant difference. In contrast, an increase of post-mitotic GCPs (NeuN+, Ki-67−) was detectable in the EGL of *Ptch1^+/−^ Nos2^−/−^* and *Ptch1^+/+^ Nos2^−/−^* mice (Figure 6C). Concurrently, the ratio of dividing to non-dividing GCPs was similar in *Ptch1^+/−^ Nos2^−/−^*, wild-type and *Ptch1^+/−^ Nos2^+/+^* P9 cerebella but markedly decreased in *Ptch1^+/+^ Nos2^−/−^* mice. This recapitulated the downregulation of mitotic genes observed in the expression profiles. However, the total amount of proliferating GCPs per EGL section was significantly higher in *Ptch1^+/−^ Nos2^−/−^* mice compared to any other genotype (Figure 6C). These results demonstrate a tissue phenotype that corresponds to the effects of reduced *Gap43* in developing cerebellar neuronal precursors (*in vitro*). The increased accumulation of proliferating GCPs in the EGL observed in the *Ptch1^+/−^ Nos2^−/−^* genotype supposedly leads to a larger pool of cells susceptible to neoplastic transformation and is therefore likely to promote medulloblastoma development.

## Discussion

The *Ptch1^+/−^* MB mouse model has been intensively studied and has greatly contributed to our understanding of Hh-dependent MB tumorigenesis in the context of cerebellar development. The data presented here indicate a role of Nos2 and hence NO signaling in Hh-dependent MB by demonstrating a significantly increased MB rate in *Ptch1^+/−^ Nos2^−/−^* mice compared to *Ptch1^+/−^ Nos2^+/+^* mice. The global genome-wide screens performed in the present study did not reveal obvious molecular differences between MBs in *Ptch1^+/−^ Nos2^+/+^* versus *Ptch1^+/−^ Nos2^−/−^* animals. Assessment of genomic alterations using array-CGH identified trisomy of chromosome 6 as a recurrent feature in tumors of both genotypes. This corresponds to a recent report on MBs of the same molecular subtype with inactivated double-strand break repair proteins targeted to neuronal progenitors of *p53^−/−^* mice [42]. The most common loss identified in our analyses affected two small regions on chromosome 13 encompassing the *Ptch1* gene and possibly indicate acquired homozygosity for the mutant allele or somatic rearrangements rather than a broad deletion of the locus. Targeted duplex PCR further confirmed loss of the functional wild-type allele to be a frequent event in these MBs. Notably, tumors of the *Ptch1^+/−^ Nos2^−/−^* genotype showed a higher frequency of a small gain on chromosome 14. The affected *Entpd4* gene encodes for an apyrase located at the internal membrane of lysosomal vacuoles and the Golgi apparatus. It preferentially catalyzes the hydrolysis of UDP to UMP [43] and thereby facilitates the inverse directed import of UDP-GlcNAc [44]. This in turn was reported to increase glycosylation of surface receptors (e.g. EGFR and PDGFR) and foster cell growth [45]. According to the microarray and qRT-PCR expression data, *Entpd4* transcript levels were indeed increased in tumors with this chromosomal gain. However, this effect did not turn out to be *Nos2*-dependent in an expanded sample set. Consequently, *Entpd4* likely plays a role in MB pathogenesis but is not directly linked to loss of Nos2.

The examination of tumor-relevant changes in developing cerebellum as a consequence of impaired Nos2 activity and hence NO signaling surprisingly revealed a decreased proliferation of GCPs in the cerebellum of *Ptch1^+/+^ Nos2^−/−^* mice. The concurrent upregulation of *Ptch1* and the significant enrichment of downregulated Gli1-target genes strongly suggest that this effect is a consequence of reduced hedgehog signaling. Moreover, this phenotype was completely abrogated by a concomitant *Ptch1* mutation. The slight increase of *Ptch2* in *Ptch1^+/−^ Nos2^−/−^* cells points to a compensatory effect and further supports the notion of an inhibitory function of Nos2 loss on the hedgehog pathway in postnatal cerebellum. Since neither a Smoh-regulating domain [38] nor a function for cell cycle arrest through seizing cyclin B1 [46] were reported for Ptch2, its upregulation may be insufficient for preventing MB induction. In contrast to these observations, Ciani *et al.* demonstrated that proliferation of cultured GCPs increased upon withdrawal of NO and that this effect was mediated by augmented N-myc levels [37]. However, N-myc was not differentially expressed between *Ptch1^+/−^ Nos2^−/−^* and *Ptch1^+/−^ Nos2^+/+^* MBs of our series. A possible explanation for this discrepancy might be an unrecognized heterogeneity in the isolated cerebellar cell population used in the Ciani study. Since eNos and nNos are known to attenuate the mitotic activity of subventricular neuronal stem cells [47], [48] Nos inhibitor treatment possibly resulted in a selective growth advantage over GCPs. Downregulation of Gap43 was the only feature observed in *Nos2*-deficient versus *Nos2*-proficient postnatal cerebella irrespective of the *Ptch1* status. This difference was also conserved between *Ptch1^+/−^ Nos2^−/−^* and *Ptch1^+/−^ Nos2^+/+^* MBs, and particularly visible in outer regions of the molecular layer, where maturating GCPs of the EGL develop contact forming projections prior to radial migration. Other studies already suggested a link between *Gap43* mRNA levels and NO signaling due to co-induction of *nNos* and *Gap43* during axon regeneration and reactive synaptogenesis following injury of spinal motoneurons [49] and sensory neurons [50]. Furthermore, a downregulation of *Gap43* was reported after silencing of soluble guanylate cyclase subunits, the central elements of cGMP-mediated NO signaling [51]. Finally, the present study demonstrates *Gap43* downregulation to be a consequence of NO withdrawal in neuronal progenitors and MB cells. A possible mechanism for this regulation refers to decreased protein levels of the poly(U)-binding and degradation factor AUF1 upon NO-dependent cGMP production [52]. AUF-proteins generally bind to AU-rich elements of the 3′UTR (untranslated region) of coding transcripts and associate with proteins of the ELAV-like family to control gene expression via mRNA decay [53]. Tsai *et al.* demonstrated that *Gap43* mRNA levels are post-transcriptionally regulated during neuronal differentiation and that elements of the 3′UTR confer transcript instability, which is abolished upon TPA treatment (*inter alia* inducing *NOS2*) [54]. At the same time, Chung *et al.* demonstrated that indeed ELAV-like family member HuD was binding to 3′UTR regions of *GAP43* [55]. Taken together, NO accumulation possibly decreases cellular levels of mRNA-destabilizing AUF1 protein and thus might contribute to a high transcript abundance of *Gap43*.

Gap43 is a membrane-anchored protein at the cytoplasmic side of neuronal cell projections and found to be highly expressed during development of the CNS [56]. It is particularly localized in axonal growth cones and participates in the coordination of extrinsic stimuli and intrinsic cell remodeling [57] by regulating cytoskeleton dynamics [58]. Granule cell (GC) migration follows a sequence of tangential and radial movements controlled by successive formation of leading projections [59]. As maturating GCPs exit cell cycle, positioning of the centrosome determines the site of axon growth cone emergence and thus neuronal polarity including localization of such projections [60]. This defines the structural orientation of GCPs in terms of directing its dendrite to descend across the molecular and Purkinje cell layers to populate the IGL. However, centrosome positioning and therefore accurate polarization of GCPs require phosphorylated Gap43 to bind to the centrosome-associated microtubule-organizing center [61]. Hence, inaccurate GCP migration was observed in *Gap43^−/−^* animals [39], a finding that is in full agreement with our data from the functional Gap43 knockdown assays. Downregulation of *Gap43* in *Nos2*-deficient P9 cerebellum therefore likely mediates the retention of GCPs observed in FFPE sections. Accordingly, NO/cGMP signaling was demonstrated to be crucial for accurate migration of the neuronal precursor cell line NT2 [62]. Furthermore, slice culture experiments of neonatal cerebella (P9) exhibited a substantial reduction of proliferation and migration of maturating granule cells to the IGL upon application of NO synthases inhibitors [63]. The elevation of *Ptch1* levels upon Gap43 reduction *in vitro* fits to the data by Shen *et al.* who reported an upregulation of *Ptch1* gene expression in inner EGL regions of *Gap43^−/−^* mice compared to wild-type animals. Moreover, cultured *Gap43*-deficient GCPs show decreased proliferation in response to administered recombinant Shh protein [64]. A possible regulatory link was recently provided as the activation of the hedgehog signaling component Smoh was found to depend on PI4P (phosphatidylinositol 4-phosphate) levels that immediately increase when Shh binds to Ptch1 or when functional Ptch1 is absent [65]. The authors further showed that imbalanced conversion of the precursor molecule PI into PI4P influences hedgehog pathway activity. Alternatively, the production of PI4P can also result from a specific dephosphorylation of PI(4,5)P2 [66]. In this context, Gap43 protein was recently demonstrated to build oligomeric structures in the plasma membrane which sequester specifically PI(4,5)P2 [67]. A similar finding has been reported earlier showing that GAP43 participates in the accumulation of plasmalemma rafts, which promoted retention of PI(4,5)P2 [68]. The amount of Gap43 associated with the plasma membrane therefore possibly modulates the utilization of PI(4,5)P2, including its conversion into PI4P, which in turn directly affects hedgehog signaling through Smoh activation. However, the effective impact on downstream Gli-targets would still be difficult to conclude regarding the multitude of responses to Shh, including negative feedback regulation [13]. Further studies applying depletion and enrichment of specific phosphatidyl derivatives and selective silencing of hedgehog pathway elements will be necessary to elucidate the molecular nature of this proposed signaling axis.

**Figure 7:**
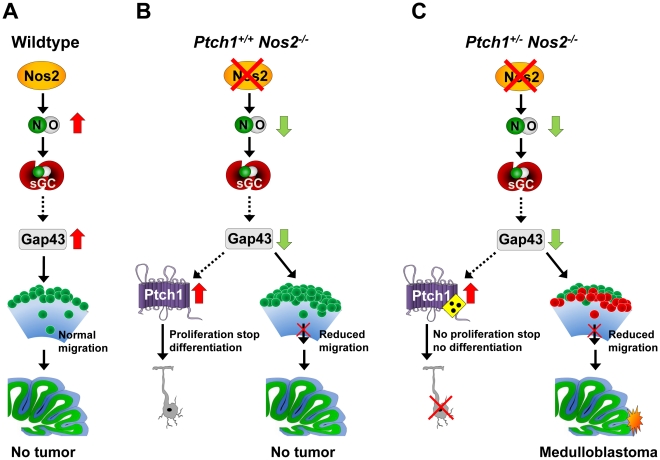
Proposed mechanism promoting MB development by *Nos2* inactivation in *Ptch1*-mutant mice. (A) Under normal conditions Nos2 generates NO in immature GCPs, which increases/stabilizes *Gap43* transcript levels and permits accurate migration. (B) Inactivation of Nos2 decreases NO, which reduces *Gap43* expression. As a result, cell polarization, and thus directed migration of GCPs is impaired. Concomitant upregulation of functional *Ptch1* stops proliferation and prevents tumor formation. (C) In combined *Ptch1^+/−^ Nos2^−/−^* cerebellum GCP migration is also impaired due to reduced Gap43 levels. Upregulation of mutant *Ptch1* fails to arrest cell cycle. Both effects in combination lead to accumulation of proliferating GCPs and promote MB development. NO: nitric oxide, sGC: soluble guanylate cyclase.

The increased accumulation of mitotic granule cells at the EGL seen in the combined *Ptch1^+/−^ Nos2^−/−^* genotype supposedly gives a special clue to MB induction. In contrast to the classical view of neonatal EGL organization, which describes radial migration of granule cells to follow a proliferation stop, more and more evidence arises showing that cell cycle arrest is not a prerequisite for migration but rather occurs during a temporally coordinated interplay of gene expression patterns [69]. This corroborates our data shown in Figure 6 (white arrows), where proliferation is still maintained in migrating granule cells and even in cells of the IGL of wild-type cerebellum. Regulation of such expression patterns is largely dependent on Shh stimuli being most intensive in the EGL [70], as well as gradients of other soluble factors such as Bmps, which were reported to account for a regulatory environment along the transition through the cerebellar layers [71]. Further evidence for a niche-like-concept was provided by Choi *et al.* in *Bdnf^−/−^* mice that displayed a severe retardation of GCP migration [16]. The authors could demonstrate that mitotic activity of maturating GCPs was significantly enhanced when cells were retained in the EGL and declined with increasing distance from outer EGL regions. Therefore, the accumulation of GCPs in the EGL in combination with the insensitivity to *Ptch1*-mediated cell cycle arrest in *Ptch1^+/−^ Nos2^−/−^* mice provide a growth advantage and increase the number of putative transformation targets over *Ptch1^+/−^ Nos2^+/+^* mice (Figure 7C).

In conclusion, the following picture emerged from our data: Homozygous deletion of *Nos2* leads to a reduction of basic NO levels in immature GCPs of the EGL during postnatal development of the cerebellum. This reduction causes a downregulation of *Gap43* expression, which results in an increased expression of *Ptch1* and impaired directed migration of maturating GCPs. As a consequence, undifferentiated granule cell precursors exit cell cycle and are retained at the EGL (Figure 7B). In case of an additional heterozygous *Ptch1* mutation, upregulation of this receptor does not suffice to exert the anti-proliferative stimulus following *Gap43* decrease, which results in an increased fraction of continuously dividing cells in the EGL (Figure 7C). As reduced migration towards the IGL further leads to a withdrawal of growth-limiting signals, expansion of the GCP population is additionally supported. Finally, this advances medulloblastoma development in *Ptch1^+/−^ Nos2^−/−^* mice compared to *Ptch1^+/−^ Nos2^+/+^* mice. The mechanism described here illustrates a new tumor-promoting concept in MB showing that the localization of pre-neoplastic cells within the developing cerebellum is important for pathogenesis.

## Materials and Methods

### Generation of *Ptch1^+−^ Nos2^−/^* ^−^ mice

*Ptch1^+/−^* mice (B6; 129P2-Ptch1^tm1Mps^/Ptch^+^; [20]) and *Nos2^−/−^* mice (B6;129P2-*Nos2* ^tm1Lau^; [72]) were obtained from the Jackson Laboratory (Bar Harbor, Maine, USA) and crossbred to generate double heterozygous mice (*Ptch1^+/−^ Nos2^+/^* ^−^). The F1 hybrids were backcrossed with *Ptch1^+/+^ Nos2^−/^* ^−^ mice to generate *Ptch1^+/−^ Nos2^−/^* ^−^ mice. Later on, *Ptch1^+/−^ Nos2^−/^* ^−^ mice were directly mated. For details on housing and genotyping see Text S2 and Table S9. All animal experiments were approved by the responsible federal authorities (Landesamt für Natur, Umwelt und Verbraucherschutz Nordrhein-Westfalen, Recklinghausen, Germany, Az. 50.05-230-17/06).

### Microarray-based genomic and expression profiling

Total RNA of tumor specimens and normal cerebellar tissue samples was isolated via CsCl density gradient centrifugation [73] and assessed for integrity using an Agilent 2100 Bioanalyzer (Agilent Technologies, Santa Clara, USA). For gene expression microarrays, linear amplification of mRNA and labeling of cDNA were conducted on samples and Mouse Universal Reference RNA (Stratagene, La Jolla, USA) according to the TAcKLE protocol [74]. Both were combined for two-color hybridizations with each sample being performed as two replicates of inverse dye orientation. Global gene expression profiling was performed on self-printed oligonucleotide microarrays. Further details of microarray production and hybridization are described in Text S2. Genomic DNA of tumor specimens was isolated from the interphase of the CsCl gradient by ethanol precipitation, proteinase K digest, and phenol/chloroform extraction. DNA samples were monitored for purity and adequate fragment size using spectrophotometric measurements and gel electrophoresis. Array-based comparative genomic hybridization (array-CGH, matrix-CGH, [75]) was performed on Mouse Genome CGH 244 k Microarrays (Agilent). Cy5-labeled tumor DNA was combined with corresponding Cy3-labeled reference (wild-type genomic DNA) to receive either sex-matched sample pairs or pairs of different gender for internal negative or positive control. Sample preparation, microarray hybridization, and washing procedures were carried out as described in the manufacturer's protocol. Microarray data are available in GEO (http://www-ncbi.nlm.nih.gov/geo), under accession number GSE29201.

### qRT–PCR analyses

Total RNA isolated from cell culture samples using the RNeasy Mini Kit (Qiagen, Hilden, Germany) or RNA from tissue specimens was subjected to oligo(dT)-primed reverse transcription. QRT-PCR measurements were conducted in an ABI PRISM 7900HT thermal cycler (Applied Biosystems, Foster City, USA) using the SYBR green reaction and detection system (ABgene, Epsom, UK). For relative quantification mean ratios were calculated between genes of interest and a set of five housekeeping genes (Table S7) according to the Pfaffl method [76].

The expression levels of *Ptch1*, *Gli1*, *N-myc*, and *Nos2* were determined by real-time reverse transcription PCR analysis using the ABI PRISM 5700 system (Applied Biosystems) as reported before [73]. For these experiments, the mRNA expression level of mitochondrial ribosomal protein L32 (*Mrpl32*) served as housekeeping reference. All primer sequences are depicted in Table S8.

### Cell culture experiments

Inhibition of NO synthases was performed in c17.2 and D458 cells which were seeded at densities of 2×10^5^ and 4×10^5^ cells per well in 12-well plates, respectively. Cells were daily treated with either 1 mM of the inhibitor L-NAME or 1× PBS as solvent control. For knockdown experiments of Gap43, c17.2 cells were grown in a 12-well plate to 80% confluency and transfected with 2 µg of pLKO.1-puro vector that contained either shRNA constructs targeting Gap43, shRNA against GFP, or non-target shRNA as a control (Sigma-Aldrich, St. Louis, USA) using 9 µl FuGene HD reagent (Roche, Basel, Switzerland). Transfection was repeated 2 times each after 8 hours and subjected to selection conditions (1 µg/ml puromycin) for 24 hours. Subsequently, cells were trypsinized, adjusted to 4×10^5^ cells/ml and seeded into the inserts of a Costar Polycarbonate Membrane Transwell plate (8 µm pores, Corning, USA). After 24 hours cells were either harvested for gene expression and protein analyses or 0.1 µg/µl recombinant SDF-1α was applied to the lower compartment for migration assays. Following 12 hours of incubation, cells at the bottom of the insert membrane were methanol-fixed, hematoxylin-stained, and counted.

### Immunofluorescence analyses

FFPE sections of postnatal cerebella were pre-processed as described in Text S2. For immunofluorescence co-staining, Gap43 (Sigma-Aldrich, clone GAP-7b10) or NeuN (Millipore, clone A60) first primary antibodies were diluted 1∶1000 or 1∶200, respectively and applied using the Dako REAL Detection System (Dako, Glostrup, Denmark). Following over night incubation at 4°C, washing in TBS, and blocking of residual biotin/streptavidin, sections were subsequently incubated with biotinylated anti-mouse secondary antibody (Dako) and stained with 20 ng/µl FITC-conjugated streptavidin (Invitrogen, Carlsbad, USA). The second primary antibody against Ki-67 (Novocastra, Wetzlar, Germany) was diluted 1∶1000 and accordingly applied using biotinylated anti-rabbit secondary antibody (Dako) and 20 ng/µl Cy5-conjugated streptavidin (Invitrogen). Co-stained sections were then covered with DAPI-containing VECTASHIELD Mounting Medium (Vector, Burlingame, USA) and subjected to confocal laser scanning microscopy.

Quantification of Gap43 staining was performed for areas of interest using Image J software (NIH). Numbers of dividing and non-dividing cells in the EGL of postnatal cerebellar tissue sections were counted manually and normalized to the corresponding length of the EGL edge. Cell counts for each region were averaged across three sections, each with 10–20 µm distance in z-axis, and per individual.

### Statistical analyses

Kaplan-Meier survival plots were calculated for a total of 1167 mice, including 315 wild-type mice, 412 *Ptch1^+/+^ Nos2^−/−^* mice, 215 *Ptch1^+/−^ Nos2^+/+^* mice and 221 *Ptch1^+/−^ Nos2^−/−^* mice. MB-free survival was plotted using the GraphPad Prism 5 software (GraphPad, La Jolla, USA). The logrank test was applied to compare survival (tumor occurrence) of the different genotypes. For comparisons of differences of means between two groups of replicates, p-value calculations were performed using an unpaired, two-tailed t-test, unless indicated otherwise. Calculated error bars represent the standard error of the mean (SEM). For details on microarray statistics please refer to Text S2.

## Supporting Information

Figure S1 Expression of *Ptch1*, *Gli1*, and *N-myc* in medulloblastomas. (A) Expression of *Ptch1* is significantly downregulated in medulloblastomas of both genotypes relative to normal adult cerebellum. (B–C) *Gli1* and *N-myc* transcripts are significantly upregulated relative to the same control cerebella. Shown are mRNA expression levels (means with standard deviation) of 8 *Ptch1* ^+/−^ *Nos2* ^+/+^ and 13 *Ptch1* ^+/−^ *Nos2* ^−/−^ MBs as well as 12 normal cerebellum samples (NCB) of each genotype. All data are based on qRT-PCR results calculated relative to the reference gene *MrpL32* and normalized to mouse universal reference RNA (Stratagene). Significant differences are indicated (*p<0.05, **p<0.01, ***p<0.001, Mann-Whitney U test or ANOVA test, respectively).
(TIF)

Figure S2 N-myc protein is not differentially expressed between *Ptch1^+/−^ Nos2^+/+^* and *Ptch1^+/−^ Nos2^−/−^* medulloblastomas. (A) Western blot analysis of N-myc protein expression in separated protein extracts from tumor specimens of three *Ptch1^+/−^ Nos2^+/+^* and five *Ptch1^+/−^ Nos2^−/−^* mice, as well as healthy adult cerebellum of wild-type, *Ptch1^+/−^ Nos2^+/+^* and *Ptch1^+/−^ Nos2^−/−^*. Depicted is stained for N-myc and α-tubulin (housekeeping protein). (B) Quantification of N-myc protein bands normalized to α-tubulin, p = 0.34, Δmean = −0.149±0.144.
(TIF)

Figure S3 Validation of candidate genes that were observed to be differentially expressed between *Ptch1^+/−^ Nos2^+/+^* and *Ptch1^+/−^ Nos2^−/−^* tumors in the microarray data. Linear expression values were obtained from qRT-PCR measurements on the expanded sample set and indicate mRNA expression against a pool of housekeeping genes normalized to mouse universal reference RNA (Stratagene). (A) Expression of *Otx1*, p = 0.071, Δmean = −2.738±1.354. (B) Expression of *Pdgfra*, p = 0.281, Δmean = −0.959±0.849. (C) Expression of *Stmn1*, p = 0.710, Δmean = 0.202±0.531.
(TIF)

Figure S4 Nitric oxide (NO) assay on neuronal progenitor cells (c17.2) and medulloblastoma cells (D458) upon inhibition of NO synthases. Treatment samples were supplemented with 1 mM L-NAME and control samples were supplemented with solvent (PBS). After 24 hours control sample exceeded NO levels in treatment samples indicating successful impairment of NO production.
(TIF)

Figure S5 Apoptosis and cell cycle analyses of the cell lines c17.2 and D458 by FACS, after inhibition of NO synthases (L-NAME treatment). (A, C) PI (propidium iodide) signals of fixated cells representing different cell cycle phases. (B, D) Dot plot showing apoptosis in freshly harvested cells stained with Annexin V and 7-AAD. (E) Plotted fractions of cells in G_0_/G_1_, G_2_/M-phase, or S-phase. (F) Plotted fractions of dead cells and viable cells. Inhibition of NO synthases by L-NAME application shows no prominent changes in cell physiology. I: cell debris, II: G_0_/G_1_, III: S-phase, IV: G_2_/M-phase, V: doublets.
(TIF)

Figure S6 Reduction of c17.2 migration depends on Gap43 knockdown efficiency. (A) Western blot stained for Gap43 and α-tubulin (housekeeping protein). (B) Quantification of Gap43 protein bands normalized to α-tubulin. (C) Proportion of migrated cells relative to non-target (NT) control. The constructs sh39 and sh42 showed the highest knockdown efficiency and the strongest effect on migration.
(TIF)

Figure S7 Cell cycle analysis of neuronal progenitor cells (c17.2) by FACS, 72 h after knockdown of *Gap43*. (A) PI (propidium iodide) signals of fixated cells representing different cell cycle phases. I: cell debris, II: G_0_/G_1_, III: S-phase, IV: G_2_/M-phase, V: doublets. (B) Plotted fractions of cells in G_0_/G_1_, G_2_/M-phase, or S-phase.
(TIF)

Table S1 Targeted molecular analyses of selected genes.
(DOC)

Table S2 Differentially expressed genes in medulloblastomas of *Ptch1^+/−^ Nos2^−/−^* against *Ptch1^+/−^ Nos2^+/+^* mice.
(DOC)

Table S3 Gene expression of markers for stromal cells in medulloblastomas of *Ptch1^+/−^ Nos2^−/−^* against *Ptch1^+/−^ Nos2^+/+^* mice.
(DOC)

Table S4 Differentially expressed genes in P9 cerebella of *Ptch1^+/+^ Nos2^−/−^* against wild-type mice.
(DOC)

Table S5 Differentially expressed genes in P9 cerebella of *Ptch1^+/−^ Nos2^+/+^* against wild-type mice.
(DOC)

Table S6 Differentially expressed genes in P9 cerebella of *Ptch1^+/−^ Nos2^−/−^* against wild-type mice.
(DOC)

Table S7 Housekeeping genes used for qRT-PCR analyses.
(DOC)

Table S8 Primer sequences used for qRT-PCR analyses.
(DOC)

Table S9 Primer sequences used for duplex-PCR analyses and genotyping.
(DOC)

Text S1 Supporting Results.
(DOC)

Text S2 Supporting Methods.
(DOC)
