# Supplementary figures and images for: Nos2 Inactivation Promotes the Development of Medulloblastoma in Ptch1+/− Mice by Deregulation of Gap43–Dependent Granule Cell Precursor Migration
Source: PLoS Genet. 2012 Mar 15;8(3):e1002572. doi: 10.1371/journal.pgen.1002572 (PMC3305407; doi:10.1371/journal.pgen.1002572)

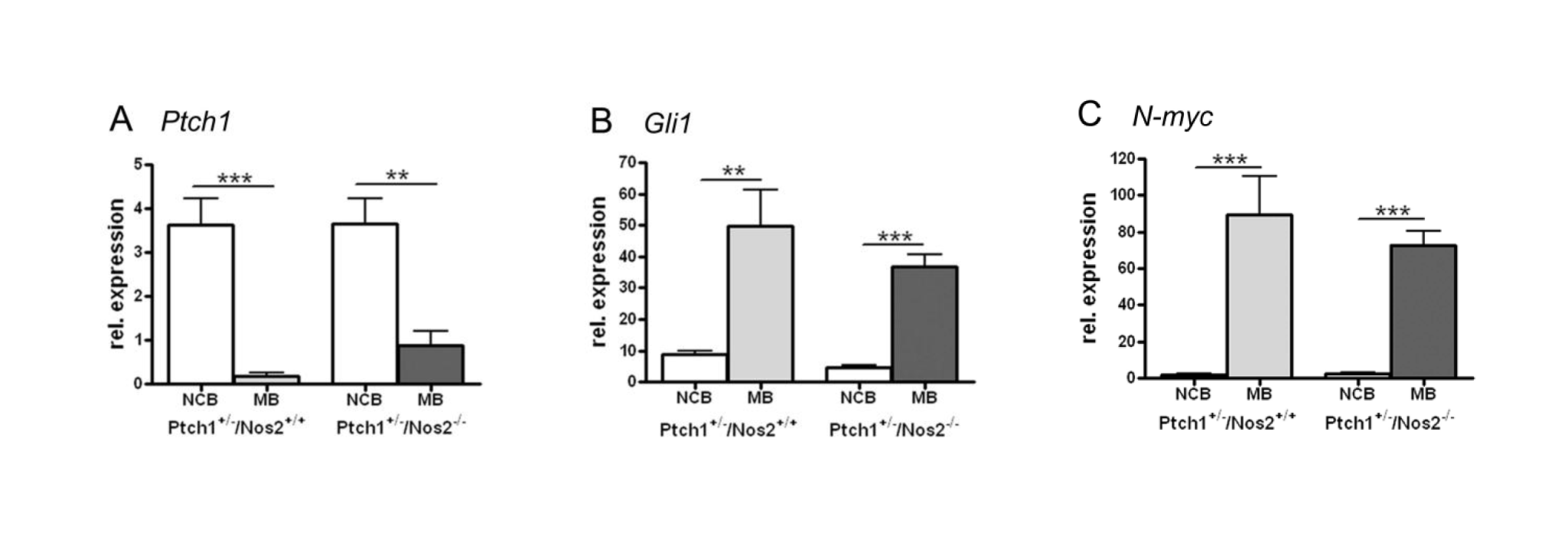

Supplement: Figure S1 — Expression of Ptch1, Gli1, and N-myc in medulloblastomas. (A) Expression of Ptch1 is significantly downregulated in medulloblastomas of both genotypes relative to normal adult cerebellum. (B–C) Gli1 and N-myc transcripts are significantly upregulated relative to the same control cerebella. Shown are mRNA expression levels (means with standard deviation) of 8 Ptch1 +/− Nos2 +/+ and 13 Ptch1 +/− Nos2 −/− MBs as well as 12 normal cerebellum samples (NCB) of each genotype. All data are based on qRT-PCR results calculated relative to the reference gene MrpL32 and normalized to mouse universal reference RNA (Stratagene). Significant differences are indicated (*p<0.05, **p<0.01, ***p<0.001, Mann-Whitney U test or ANOVA test, respectively). (TIF) [file pgen.1002572.s001.tif]

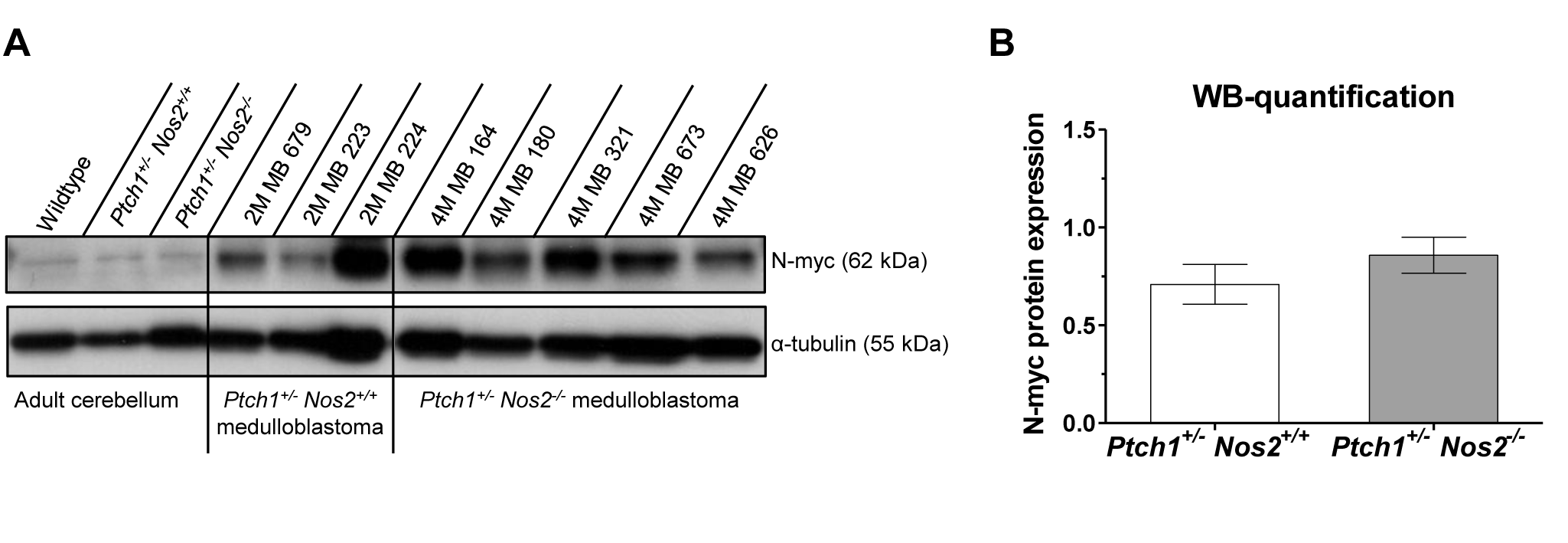

Supplement: Figure S2 — N-myc protein is not differentially expressed between Ptch1+/− Nos2+/+ and Ptch1+/− Nos2−/− medulloblastomas. (A) Western blot analysis of N-myc protein expression in separated protein extracts from tumor specimens of three Ptch1+/− Nos2+/+ and five Ptch1+/− Nos2−/− mice, as well as healthy adult cerebellum of wild-type, Ptch1+/− Nos2+/+ and Ptch1+/− Nos2−/−. Depicted is stained for N-myc and α-tubulin (housekeeping protein). (B) Quantification of N-myc protein bands normalized to α-tubulin, p = 0.34, Δmean = −0.149±0.144. (TIF) [file pgen.1002572.s002.tif]

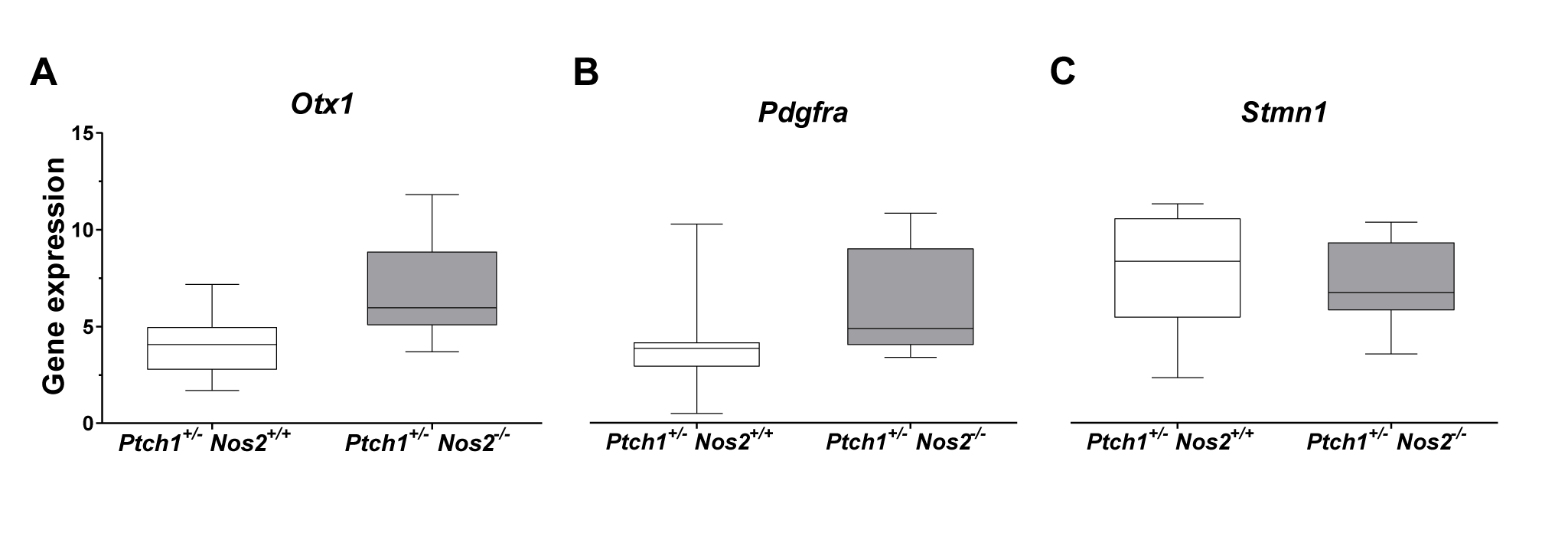

Supplement: Figure S3 — Validation of candidate genes that were observed to be differentially expressed between Ptch1+/− Nos2+/+ and Ptch1+/− Nos2−/− tumors in the microarray data. Linear expression values were obtained from qRT-PCR measurements on the expanded sample set and indicate mRNA expression against a pool of housekeeping genes normalized to mouse universal reference RNA (Stratagene). (A) Expression of Otx1, p = 0.071, Δmean = −2.738±1.354. (B) Expression of Pdgfra, p = 0.281, Δmean = −0.959±0.849. (C) Expression of Stmn1, p = 0.710, Δmean = 0.202±0.531. (TIF) [file pgen.1002572.s003.tif]

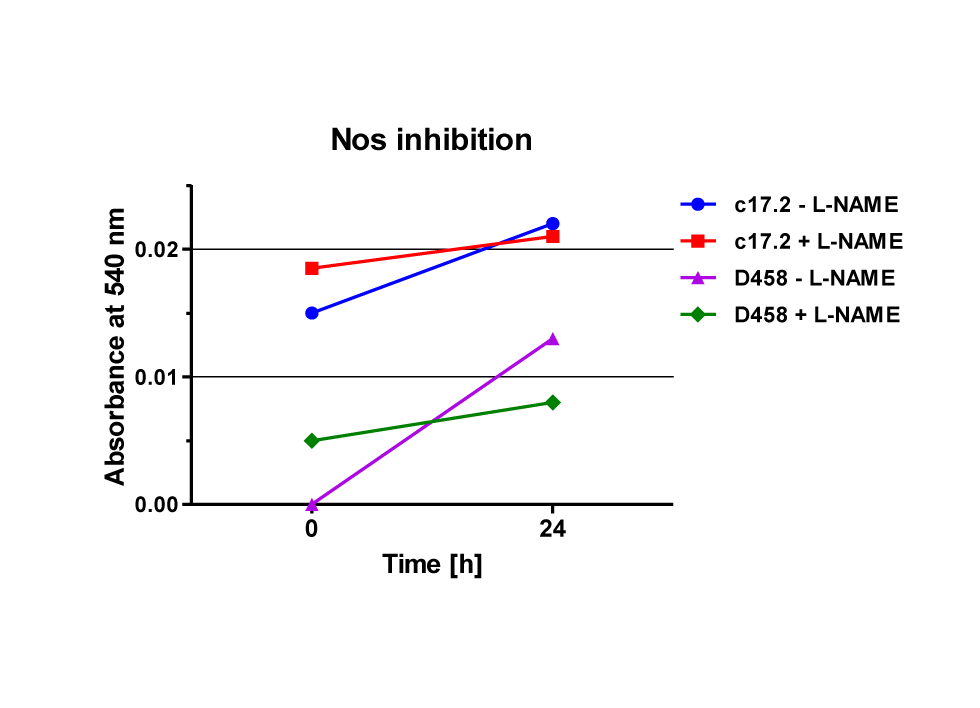

Supplement: Figure S4 — Nitric oxide (NO) assay on neuronal progenitor cells (c17.2) and medulloblastoma cells (D458) upon inhibition of NO synthases. Treatment samples were supplemented with 1 mM L-NAME and control samples were supplemented with solvent (PBS). After 24 hours control sample exceeded NO levels in treatment samples indicating successful impairment of NO production. (TIF) [file pgen.1002572.s004.tif]

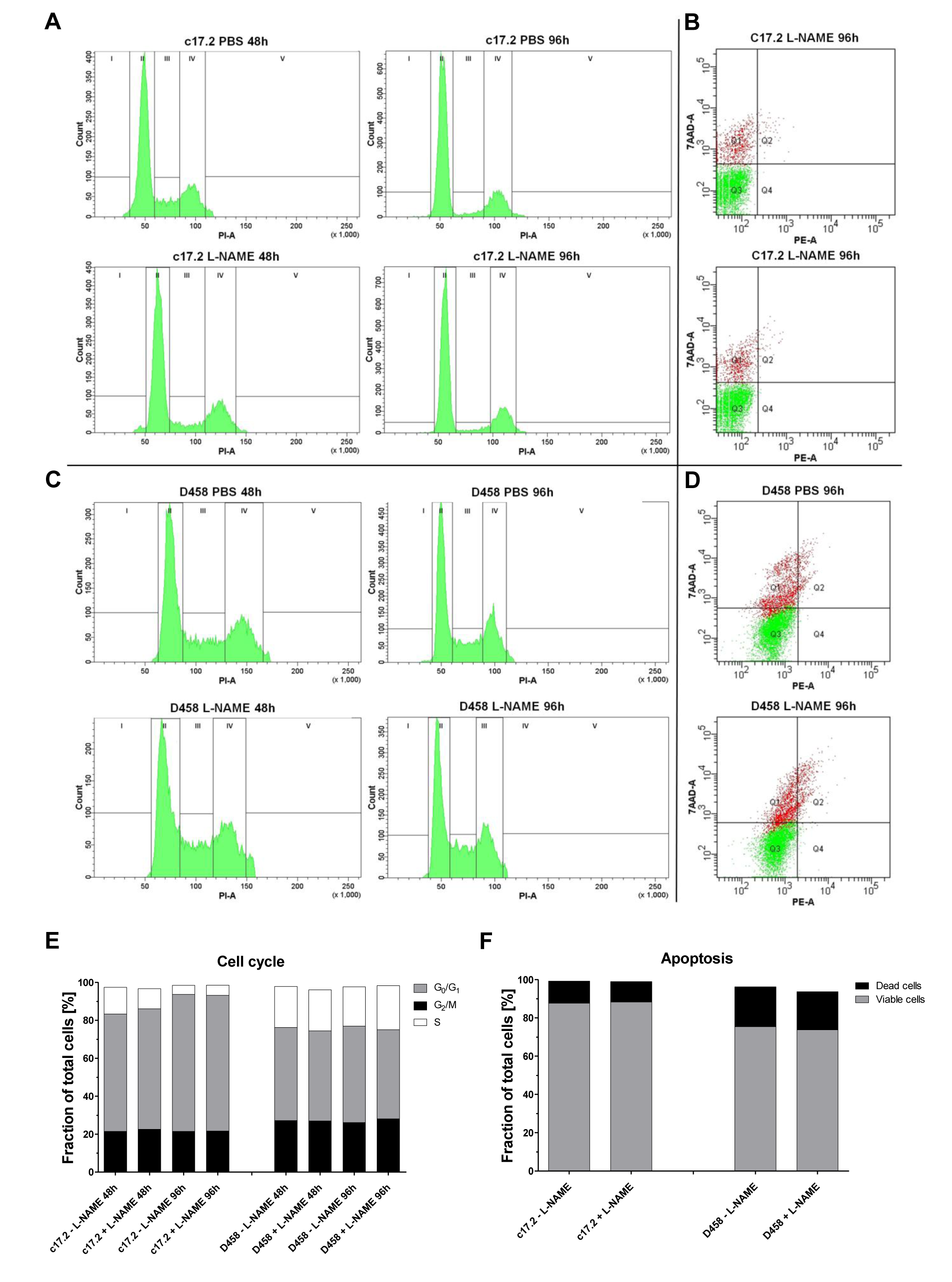

Supplement: Figure S5 — Apoptosis and cell cycle analyses of the cell lines c17.2 and D458 by FACS, after inhibition of NO synthases (L-NAME treatment). (A, C) PI (propidium iodide) signals of fixated cells representing different cell cycle phases. (B, D) Dot plot showing apoptosis in freshly harvested cells stained with Annexin V and 7-AAD. (E) Plotted fractions of cells in G0/G1, G2/M-phase, or S-phase. (F) Plotted fractions of dead cells and viable cells. Inhibition of NO synthases by L-NAME application shows no prominent changes in cell physiology. I: cell debris, II: G0/G1, III: S-phase, IV: G2/M-phase, V: doublets. (TIF) [file pgen.1002572.s005.tif]

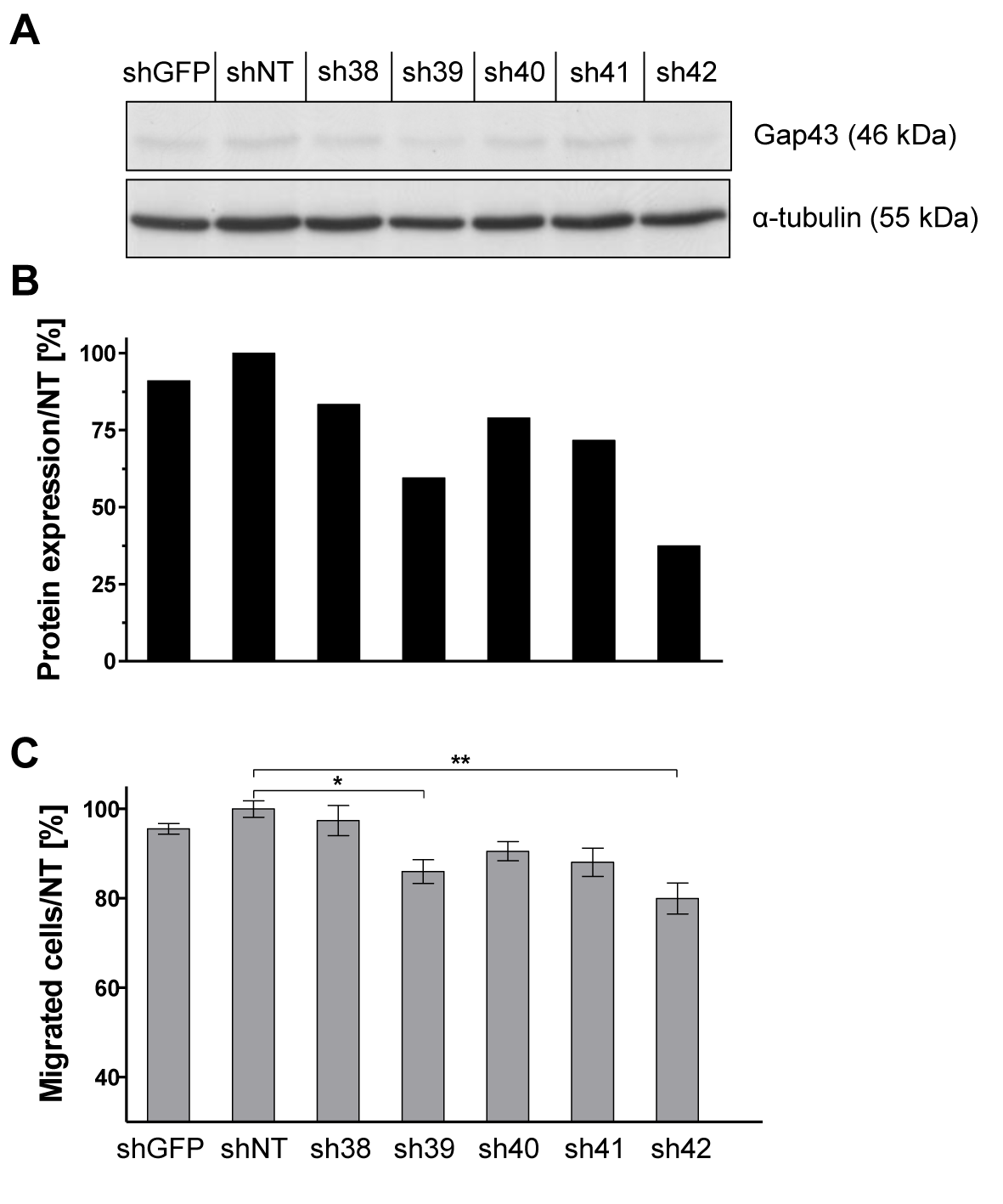

Supplement: Figure S6 — Reduction of c17.2 migration depends on Gap43 knockdown efficiency. (A) Western blot stained for Gap43 and α-tubulin (housekeeping protein). (B) Quantification of Gap43 protein bands normalized to α-tubulin. (C) Proportion of migrated cells relative to non-target (NT) control. The constructs sh39 and sh42 showed the highest knockdown efficiency and the strongest effect on migration. (TIF) [file pgen.1002572.s006.tif]

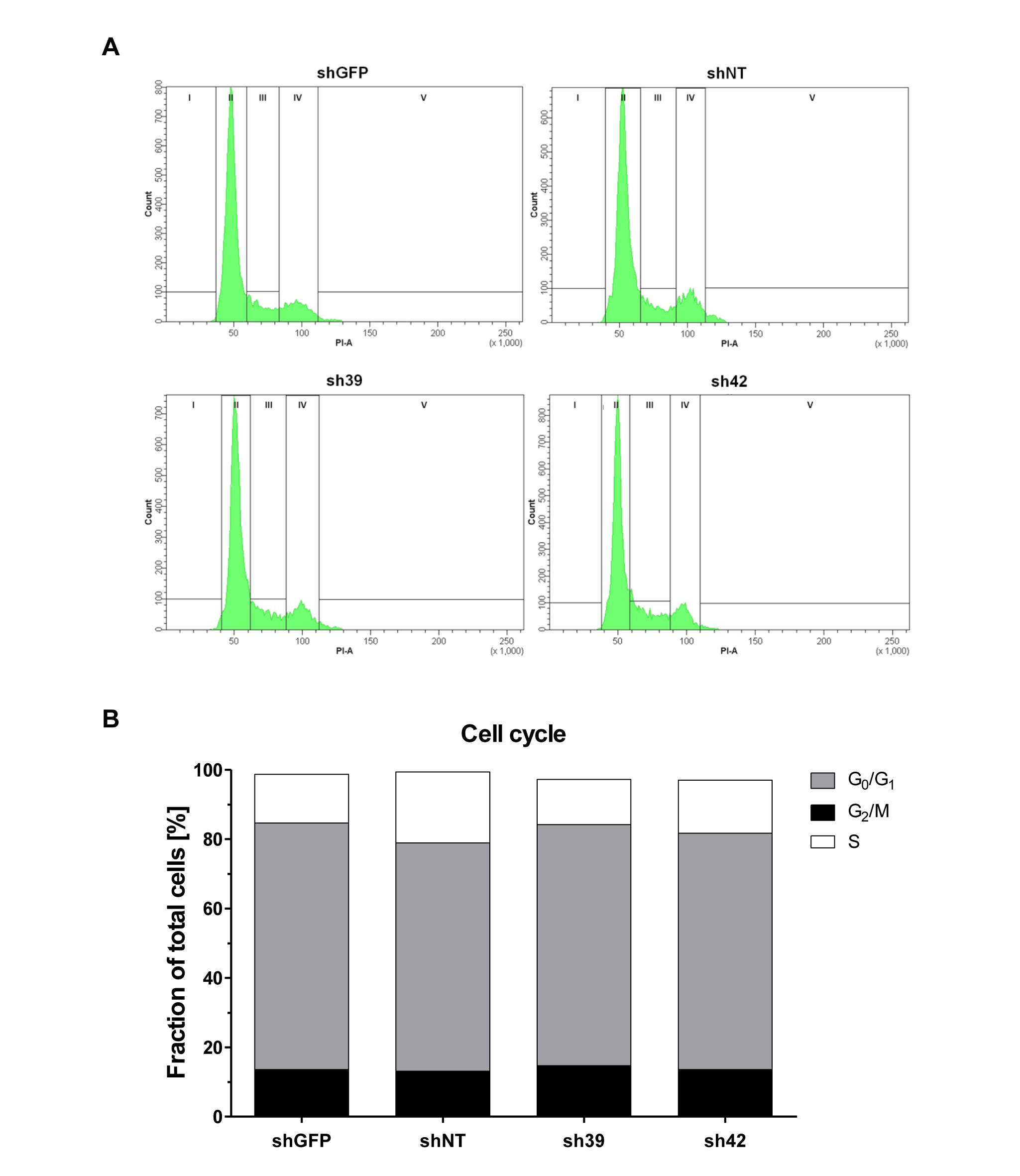

Supplement: Figure S7 — Cell cycle analysis of neuronal progenitor cells (c17.2) by FACS, 72 h after knockdown of Gap43. (A) PI (propidium iodide) signals of fixated cells representing different cell cycle phases. I: cell debris, II: G0/G1, III: S-phase, IV: G2/M-phase, V: doublets. (B) Plotted fractions of cells in G0/G1, G2/M-phase, or S-phase. (TIF) [file pgen.1002572.s007.tif]
