# Supplementary material for: Nos2 Inactivation Promotes the Development of Medulloblastoma in Ptch1+/− Mice by Deregulation of Gap43–Dependent Granule Cell Precursor Migration
Source: PLoS Genet. 2012 Mar 15;8(3):e1002572. doi: 10.1371/journal.pgen.1002572 (PMC3305407; doi:10.1371/journal.pgen.1002572)
Supplement: Table S1 — Targeted molecular analyses of selected genes. (DOC) [file pgen.1002572.s008.doc]

**Table S1:** Targeted molecular analyses of selected genes.

|  | ***Ptch1* wildtype allele** | | ***Tp53*** | ***p16^INK4a^*** | ***p19^ARF^*** |
| --- | --- | --- | --- | --- | --- |
| **Tumor** | **Deletion status** | **Expression** | **Mutation status** | **Deletion status** | **Deletion status** |
| 2M MB 11 | deleted | absent | wildtype | not deleted | not deleted |
| 2M MB 223 | present | absent | wildtype | not deleted | not deleted |
| 2N MB 224 | present | reduced | wildtype | not deleted | not deleted |
| 2M MB 227 | present | n. d. | wildtype | not deleted | not deleted |
| 2M MB 689 | present | reduced | wildtype | not deleted | not deleted |
| 2M MB 679 | deleted | absent | wildtype | not deleted | not deleted |
| 2M MB 758 | deleted | absent | wildtype | not deleted | not deleted |
| 2M MB 683 | deleted | absent | wildtype | not deleted | not deleted |
| 4M MB 14 | present | reduced | wildtype | not deleted | not deleted |
| 4M MB 164 | deleted | absent | wildtype | not deleted | not deleted |
| 4M MB 17 | deleted | reduced | wildtype | not deleted | deletion (homozygous) |
| 4M MB 180 | present/mutated | increased | wildtype | not deleted | not deleted |
| 4M MB 208 | present | absent | wildtype | not deleted | not deleted |
| 4M MB 250 | present | reduced | wildtype | not deleted | not deleted |
| 4M MB 321 | deleted | reduced | wildtype | not deleted | not deleted |
| 4M MB 457 | present | absent | wildtype | not deleted | not deleted |
| 4M MB 68 | present | increased | wildtype | not deleted | deletion (heterozygous) |
| 4M MB 626 | deleted | reduced | wildtype | not deleted | not deleted |
| 4M MB 673 | deleted | reduced | wildtype | not deleted | not deleted |
| 4M MB 751 | present | increased | wildtype | not deleted | not deleted |
| 4M MB 620 | deleted | absent | wildtype | not deleted | not deleted |

2M, *Ptch1^+/-^ Nos2^+/+^*; 4M, *Ptch1^+/-^ Nos2^-/-^*; MB, medulloblastoma; n. d., not determined
