# Supplementary material for: Nos2 Inactivation Promotes the Development of Medulloblastoma in Ptch1+/− Mice by Deregulation of Gap43–Dependent Granule Cell Precursor Migration
Source: PLoS Genet. 2012 Mar 15;8(3):e1002572. doi: 10.1371/journal.pgen.1002572 (PMC3305407; doi:10.1371/journal.pgen.1002572)
Supplement: Table S2 — Differentially expressed genes in medulloblastomas of Ptch1+/− Nos2−/− against Ptch1+/− Nos2+/+ mice. (DOC) [file pgen.1002572.s009.doc]

**Table S2:** Differentially expressed genes in medulloblastomas of *Ptch1^+/-^* *Nos2^-/-^* against *Ptch1^+/-^ Nos2^+/+^* mice.

|  | **Fold Change** | **Symbol** | **Description** | **Ensembl ID** | **Oligo ID** |
| --- | --- | --- | --- | --- | --- |
| 1 | 5.661 | Otx1* | orthodenticle homolog 1 (Drosophila) Gene | ENSMUSG00000005917 | M300000735 |
| 2 | 4.308 | Stmn1* | Dlgap1 | stathmin 1 Gene | discs, large (Drosophila) homolog-associated protein 1 Gene | ENSMUSG00000028832 | ENSMUSG00000003279 | M300006321 |
| 3 | 2.595 | Hmgcll1 | 3-hydroxymethyl-3-methylglutaryl-Coenzyme A lyase-like 1 Gene | ENSMUSG00000007908 | M300000940 |
| 4 | 2.473 | Enpp2 | ectonucleotide pyrophosphatase/phosphodiesterase 2 Gene | ENSMUSG00000022425 | M200005834 |
| 5 | 2.224 | Tspan11 | tetraspanin 11 Gene | ENSMUSG00000030351 | M200011768 |
| 6 | 2.205 | Slc25a3 | solute carrier family 25 (mitochondrial carrier, phosphate carrier), member 3 Gene | ENSMUSG00000061904 | M300001917 |
| 7 | 2.141 | Ehbp1 | EH domain binding protein 1 Gene | ENSMUSG00000042302 | M300013624 |
| 8 | 2.078 | S100a4 | S100 calcium binding protein A4 Gene | ENSMUSG00000001020 | M400011125 |
| 9 | 2.045 | Mpp6 | membrane protein, palmitoylated 6 (MAGUK p55 subfamily member 6) Gene | ENSMUSG00000038388 | M300011535 |
| 10 | 2.027 | Pdgfra* | platelet derived growth factor receptor, alpha polypeptide Gene | ENSMUSG00000029231 | M200001112 |
| 11 | 2.003 | Entpd4* | ectonucleoside triphosphate diphosphohydrolase 4 Gene | ENSMUSG00000022066 | M400011543 |
| 12 | 0.500 | Uhrf1 | ubiquitin-like, containing PHD and RING finger domains, 1 Gene | ENSMUSG00000001228 | M400011084 |
| 13 | 0.500 | Prom1 | prominin 1 Gene | ENSMUSG00000029086 | M300006480 |
| 14 | 0.499 | Nrip1 | nuclear receptor interacting protein 1 Gene | ENSMUSG00000048490 | M200003989 |
| 15 | 0.497 | Pde4dip | phosphodiesterase 4D interacting protein (myomegalin) Gene | ENSMUSG00000038170 | M300011384 |
| 16 | 0.497 | Elmod1 | ELMO domain containing 1 Gene | ENSMUSG00000041986 | M300013452 |
| 17 | 0.494 | Ncoa1 | nuclear receptor coactivator 1 Gene | ENSMUSG00000020647 | M200000772 |
| 18 | 0.492 | Klf6 | Kruppel-like factor 6 Gene | ENSMUSG00000000078 | M200012730 |
| 19 | 0.491 | Rcor1 | REST corepressor 1 Gene | ENSMUSG00000037896 | M300011204 |
| 20 | 0.491 | Gatad2b | GATA zinc finger domain containing 2B Gene | ENSMUSG00000042390 | M200008514 |
| 21 | 0.491 | Lnp | limb and neural patterns Gene | ENSMUSG00000009207 | M400016181 |
| 22 | 0.490 | Bptf | bromodomain PHD finger transcription factor Gene | ENSMUSG00000040481 | M400000293 |
| 23 | 0.489 | Epm2aip1 | EPM2A (laforin) interacting protein 1 Gene | ENSMUSG00000046785 | M200013191 |
| 24 | 0.489 | Nav3 | neuron navigator 3 Gene | ENSMUSG00000020181 | M300002051 |
| 25 | 0.488 | Ankrd12 | ankyrin repeat domain 12 Gene | ENSMUSG00000034647 | M400018310 |
| 26 | 0.487 | Gpm6a | glycoprotein m6a Gene | ENSMUSG00000031517 | M400001380 |
| 27 | 0.485 | Sema3f | sema domain, immunoglobulin domain (Ig), short basic domain, secreted, (semaphorin) 3F Gene | ENSMUSG00000034684 | M300009480 |
| 28 | 0.484 | Myod1 | myogenic differentiation 1 Gene | ENSMUSG00000009471 | M200000620 |
| 29 | 0.484 | Fbxl4 | F-box and leucine-rich repeat protein 4 Gene | ENSMUSG00000040410 | M300012645 |
| 30 | 0.483 | Gabbr2 | gamma-aminobutyric acid (GABA) B receptor 2 Gene | ENSMUSG00000039809 | M400008218 |
| 31 | 0.483 | Baz1a | bromodomain adjacent to zinc finger domain 1A Gene | ENSMUSG00000035021 | M300002514 |
| 32 | 0.483 | Atp8a1 | ATPase, aminophospholipid transporter (APLT), class I, type 8A, member 1 Gene | ENSMUSG00000037685 | M400002040 |
| 33 | 0.482 | Rbm27 | RNA binding motif protein 27 Gene | ENSMUSG00000024491 | M400009083 |
| 34 | 0.482 | Nbea | neurobeachin Gene | ENSMUSG00000027799 | M400016898 |
| 35 | 0.481 | Upf2 | UPF2 regulator of nonsense transcripts homolog (yeast) Gene | ENSMUSG00000043241 | M400002223 |
| 36 | 0.480 | A230054D04Rik | RIKEN cDNA A230054D04 gene Gene | ENSMUSG00000061755 | M300020931 |
| 37 | 0.477 | Klf7 | Kruppel-like factor 7 (ubiquitous) Gene | ENSMUSG00000025959 | M400019024 |
| 38 | 0.475 | Nr2c2 | nuclear receptor subfamily 2, group C, member 2 Gene | ENSMUSG00000005893 | M400000114 |
| 39 | 0.474 | AC166113.3 | Protein FAM123A | ENSMUSG00000021986 | M200010465 |
| 40 | 0.473 | Lmo7 | LIM domain only 7 Gene | ENSMUSG00000033060 | M400001528 |
| 41 | 0.472 | Dtna | dystrobrevin alpha Gene | ENSMUSG00000024302 | M400009066 |
| 42 | 0.471 | Samd4b | sterile alpha motif domain containing 4B Gene | ENSMUSG00000037513 | M300010971 |
| 43 | 0.468 | Srrm2 | serine/arginine repetitive matrix 2 Gene | ENSMUSG00000039218 | M300012031 |
| 44 | 0.467 | Tcf4 | transcription factor 7-like 2, T-cell specific, HMG-box Gene | ENSMUSG00000053477 | M400009098 |
| 45 | 0.466 | Mef2c | myocyte enhancer factor 2C Gene | ENSMUSG00000005583 | M200004751 |
| 46 | 0.465 | Sbno1 | sno, strawberry notch homolog 1 (Drosophila) Gene | ENSMUSG00000038095 | M400002098 |
| 47 | 0.461 | Klf3 | Kruppel-like factor 3 (basic) Gene | ENSMUSG00000029178 | M200006166 |
| 48 | 0.459 | Klf9 | Kruppel-like factor 9 Gene | ENSMUSG00000033863 | M300008987 |
| 49 | 0.458 | Taok1 | TAO kinase 1 Gene | ENSMUSG00000017291 | M400002897 |
| 50 | 0.458 | Glg1 | golgi apparatus protein 1 Gene | ENSMUSG00000003316 | M200000179 |
| 51 | 0.456 | Slc4a7 | solute carrier family 4, sodium bicarbonate cotransporter, member 7 Gene | ENSMUSG00000021733 | M400000507 |
| 52 | 0.454 | Lims1 | LIM and senescent cell antigen-like domains 1 Gene | ENSMUSG00000019920 | M300001896 |
| 53 | 0.450 | Cplx2 | complexin 2 Gene | ENSMUSG00000025867 | M300004820 |
| 54 | 0.450 | AC125535.4 | Forkhead box protein N3 (Checkpoint suppressor 1) | ENSMUSG00000033713 | M200015881 |
| 55 | 0.449 | Pb1 | polybromo 1 Gene | ENSMUSG00000042323 | M400000524 |
| 56 | 0.449 | Lmo7 | LIM domain only 7 | ENSMUSG00000033060 | M400001529 |
| 57 | 0.447 | Mgat5b | mannoside acetylglucosaminyltransferase 5, isoenzyme B Gene | ENSMUSG00000043857 | M400002805 |
| 58 | 0.444 | Zfp334 | zinc finger protein 334 Gene | ENSMUSG00000017667 | M300001598 |
| 59 | 0.443 | Prkce | protein kinase C, epsilon Gene | ENSMUSG00000045038 | M200003128 |
| 60 | 0.441 | Ncor1 | nuclear receptor co-repressor 1 Gene | ENSMUSG00000018501 | M300017976 |
| 61 | 0.438 | Qk | quaking Gene | ENSMUSG00000062078 | M300011191 |
| 62 | 0.437 | Jarid1d | jumonji, AT rich interactive domain 1D (Rbp2 like) Gene | ENSMUSG00000056673 | M400005588 |
| 63 | 0.433 | Zfp292 | zinc finger protein 292 Gene | ENSMUSG00000039967 | M400002318 |
| 64 | 0.431 | Pcdh9 | protocadherin 9 Gene | ENSMUSG00000055421 | M300018586 |
| 65 | 0.430 | Serinc5 | serine incorporator 5 Gene | ENSMUSG00000021703 | M300002908 |
| 66 | 0.426 | Pb1 | polybromo 1 Gene | ENSMUSG00000042323 | M300013631 |
| 67 | 0.422 | Dlx2 | distal-less homeobox 2 Gene | ENSMUSG00000023391 | M200001491 |
| 68 | 0.421 | Setbp1 | SET binding protein 1 Gene | ENSMUSG00000024548 | M300004201 |
| 69 | 0.413 | B230380D07Rik | RIKEN cDNA B230380D07 gene Gene | ENSMUSG00000042444 | M300013696 |
| 70 | 0.408 | Car8 | carbonic anhydrase 8 Gene | ENSMUSG00000041261 | M400002486 |
| 71 | 0.406 | Sorl1 | sortilin-related receptor, LDLR class A repeats-containing Gene | ENSMUSG00000049313 | M400003529 |
| 72 | 0.402 | Nfib | nuclear factor I/B Gene | ENSMUSG00000008575 | M300000971 |
| 73 | 0.384 | Socs2 | suppressor of cytokine signaling 2 Gene | ENSMUSG00000020027 | M200001605 |
| 74 | 0.379 | Nipbl | Nipped-B homolog (Drosophila) Gene | ENSMUSG00000022141 | M300003110 |
| 75 | 0.377 | Ankrd11 | ankyrin repeat domain 11 Gene | ENSMUSG00000035569 | M400008512 |
| 76 | 0.375 | Irs2 | insulin receptor substrate 2 Gene | ENSMUSG00000038894 | M400002204 |
| 77 | 0.372 | Arid2 | AT rich interactive domain 2 (ARID, RFX-like) Gene | ENSMUSG00000033237 | M400001539 |
| 78 | 0.370 | 4930583H14Rik | RIKEN cDNA 4930583H14 gene Gene | ENSMUSG00000037161 | M200015098 |
| 79 | 0.368 | Loxl1 | lysyl oxidase-like 1 Gene | ENSMUSG00000032334 | M300008194 |
| 80 | 0.366 | Zfp533 | zinc finger protein 385B Gene | ENSMUSG00000027016 | M300005438 |
| 81 | 0.365 | Nrxn3 | neurexin III Gene | ENSMUSG00000066392 | M300016334 |
| 82 | 0.356 | Pja2 | praja 2, RING-H2 motif containing Gene | ENSMUSG00000024083 | M200013713 |
| 83 | 0.350 | Gcap14 | granule cell antiserum positive 14 Gene | ENSMUSG00000058690 | M400000513 |
| 84 | 0.338 | Fscn1 | fascin homolog 1, actin bundling protein (Strongylocentrotus purpuratus) Gene | ENSMUSG00000029581 | M200003231 |
| 85 | 0.135 | Gap43* | growth associated protein 43 Gene | ENSMUSG00000047261 | M300018113 |
| 86 | 0.133 | Ddx3y | DEAD (Asp-Glu-Ala-Asp) box polypeptide 3, Y-linked Gene | ENSMUSG00000069045 | M400001455 |
| 87 | 0.033 | Eif2s3y | eukaryotic translation initiation factor 2, subunit 3, structural gene Y-linked Gene | ENSMUSG00000069049 | M400002945 |

*Candidate genes subjected to qRT-PCR validation.
