# Supplementary material for: Nos2 Inactivation Promotes the Development of Medulloblastoma in Ptch1+/− Mice by Deregulation of Gap43–Dependent Granule Cell Precursor Migration
Source: PLoS Genet. 2012 Mar 15;8(3):e1002572. doi: 10.1371/journal.pgen.1002572 (PMC3305407; doi:10.1371/journal.pgen.1002572)
Supplement: Table S3 — Gene expression of markers for stromal cells in medulloblastomas of Ptch1+/− Nos2−/− against Ptch1+/− Nos2+/+ mice. (DOC) [file pgen.1002572.s010.doc]

**Table S3:** Gene expression of markers for stromal cells in medulloblastomas of *Ptch1^+/-^* *Nos2^-/-^* against *Ptch1^+/-^ Nos2^+/+^* mice.

| **Cell type** | **Marker gene** | **Fold change (4M/2M)*** | **Adjusted p-value** | **Quantile rank [%]** |
| --- | --- | --- | --- | --- |
| **Microglia** | *Tlr4* (toll-like receptor 4) | 1.123 | 0.696 | 53.6 |
|  | *Trem2* (triggering receptor expressed on myeloid cells 2) | 1.094 | 0.827 | 63.9 |
|  | *Iba1/Aif1* (induction of brown adipocytes)/(allograft inflammatory factor 1) | 1.048 | 0.830 | 92.8 |
|  | *Itgam/CD11b* (integrin alpha M) | NA | NA | NA |
|  | *Ptprc/CD45* (protein tyrosine phosphatase, receptor type, C) | 1.263 | 0.467 | 42 |
| **Pericyte** | *CD248* (CD 248 antigen, endosialin) | 1.046 | 0.913 | 44.9 |
|  | *Acta2/a-SMA* (actin, alpha 2, smooth muscle, aorta) | 0.976 | 0.962 | 26.4 |
|  | *Pdgfrβ* (platelet derived growth factor receptor, beta) | 0.970 | 0.914 | 98.7 |
|  | *Cspg4/Ng2* (chondroitin sulfate proteoglycan 4)/(neuron/glial antigen 2) | 1.288 | 0.459 | 23.2 |
|  | *Cav1* (Caveolin-1) | 0.990 | 0.990 | 90.1 |
| **Vascular endothelial cell** | *CD34* antigen | 0.882 | 0.788 | 30.4 |
|  | *Pecam-1/CD31* (platelet/endothelial cell adhesion molecule 1) | 0.871 | 0.455 | 15.8 |
|  | *Icam-1/CD54* (intercellular adhesion molecule 1) | 1.205 | 0.678 | 22.2 |
|  | *Tie2* (endothelial-specific receptor tyrosine kinase) | NA | NA | NA |
|  | *Vegfa* (vascular endothelial growth factor A) | 0.853 | 0.530 | 44.9 |
|  | *Cav1* (Caveolin-1) | 0.990 | 0.990 | 90.1 |

2M, *Ptch1^+/-^ Nos2^+/+^*; 4M, *Ptch1^+/-^ Nos2^-/-^*; MB, medulloblastoma; NA, not available
