# Supplementary material for: Nos2 Inactivation Promotes the Development of Medulloblastoma in Ptch1+/− Mice by Deregulation of Gap43–Dependent Granule Cell Precursor Migration
Source: PLoS Genet. 2012 Mar 15;8(3):e1002572. doi: 10.1371/journal.pgen.1002572 (PMC3305407; doi:10.1371/journal.pgen.1002572)
Supplement: Table S4 — Differentially expressed genes in P9 cerebella of Ptch1+/+ Nos2−/− against wild-type mice. (DOC) [file pgen.1002572.s011.doc]

**Table S4:** Differentially expressed genes in P9 cerebella of *Ptch1^+/+^* *Nos2^-/-^* against wildtype mice.

|  | **Fold Change** | **Symbol** | **Description** | **Ensembl ID** | **Oligo ID** |
| --- | --- | --- | --- | --- | --- |
| 1 | 8.117 | Crtam | cytotoxic and regulatory T cell molecule Gene | ENSMUSG00000032021 | M200013961 |
| 2 | 5.772 | Lgi4 | leucine-rich repeat LGI family, member 4 Gene | ENSMUSG00000036560 | M300010436 |
| 3 | 5.583 | Mapk12 | mitogen-activated protein kinase 12 Gene | ENSMUSG00000022610 | M200008051 |
| 4 | 4.884 | Atp2a3 | ATPase, Ca++ transporting, ubiquitous Gene | ENSMUSG00000020788 | M200002343 |
| 5 | 4.837 | Inadl | InaD-like (Drosophila) Gene | ENSMUSG00000061859 | M300006158 |
| 6 | 4.405 | Slc16a11 | solute carrier family 16 (monocarboxylic acid transporters), member 11 Gene | ENSMUSG00000040938 | M400002459 |
| 7 | 4.402 | D8Ertd82e | Tyrosine-protein kinase SgK223 (EC 2.7.10.2)(Sugen kinase 223) | ENSMUSG00000050271 | M300020974 |
| 8 | 4.272 | Ntrk3 | neurotrophic tyrosine kinase, receptor, type 3 Gene | ENSMUSG00000059146 | M300014878 |
| 9 | 4.130 | Ptpn22 | protein tyrosine phosphatase, non-receptor type 22 (lymphoid) Gene | ENSMUSG00000027843 | M200000147 |
| 10 | 4.101 | Itpr1 | inositol 1,4,5-triphosphate receptor 1 Gene | ENSMUSG00000030102 | M300007005 |
| 11 | 4.101 | Ppargc1b | peroxisome proliferative activated receptor, gamma, coactivator 1 beta Gene | ENSMUSG00000033871 | M200007804 |
| 12 | 4.050 | Dgkg | diacylglycerol kinase, gamma Gene | ENSMUSG00000022861 | M400008992 |
| 13 | 4.047 | Appl2 | adaptor protein, phosphotyrosine interaction, PH domain and leucine zipper containing 2 Gene | ENSMUSG00000020263 | M200006591 |
| 14 | 3.926 | 2610207I05Rik | RIKEN cDNA 2610207I05 gene Gene | ENSMUSG00000030655 | M300007346 |
| 15 | 3.915 | Tiam1 | T-cell lymphoma invasion and metastasis 1 Gene | ENSMUSG00000002489 | M400000055 |
| 16 | 3.899 | Aldh2 | aldehyde dehydrogenase 2, mitochondrial Gene | ENSMUSG00000029455 | M200000997 |
| 17 | 3.888 | Herc3 | hect domain and RLD 3 Gene | ENSMUSG00000029804 | M300006885 |
| 18 | 3.874 | Asph | aspartate-beta-hydroxylase Gene | ENSMUSG00000028207 | M300005995 |
| 19 | 3.856 | Neurl | neuralized-like homolog (Drosophila) Gene | ENSMUSG00000006435 | M200008551 |
| 20 | 3.782 | Cbx7 | chromobox homolog 7 Gene | ENSMUSG00000053411 | M300000077 |
| 21 | 3.782 | Stmn1 | stathmin 1 Gene | ENSMUSG00000028832 | M300006321 |
| 22 | 3.758 | Serpinb1a | serine (or cysteine) peptidase inhibitor, clade B, member 1a Gene | ENSMUSG00000044734 | M400009966 |
| 23 | 3.750 | Adam1a | a disintegrin and metallopeptidase domain 1a Gene | ENSMUSG00000072647 | M200000597 |
| 24 | 3.694 | Megf11 | multiple EGF-like-domains 11 Gene | ENSMUSG00000036466 | M400009628 |
| 25 | 3.625 | Acpl2 | acid phosphatase-like 2 Gene | ENSMUSG00000043587 | M300014651 |
| 26 | 3.563 | Prkcc | protein kinase C, gamma Gene | ENSMUSG00000078816 | M400004409 |
| 27 | 3.560 | Col27a1 | collagen, type XXVII, alpha 1 Gene | ENSMUSG00000045672 | M400012980 |
| 28 | 3.523 | Aifm3 | apoptosis-inducing factor, mitochondrion-associated 3 Gene | ENSMUSG00000022763 | M300003488 |
| 29 | 3.468 | Car15 | carbonic anhydrase 15 Gene | ENSMUSG00000079715 | M400000068 |
| 30 | 3.468 | Myh14 | myosin, heavy polypeptide 14 Gene | ENSMUSG00000030739 | M300007397 |
| 31 | 3.373 | Grm4 | glutamate receptor, metabotropic 4 Gene | ENSMUSG00000063239 | M400008164 |
| 32 | 3.354 | Pip5k1b | phosphatidylinositol-4-phosphate 5-kinase, type 1 beta Gene | ENSMUSG00000024867 | M300004352 |
| 33 | 3.347 | Syne1 | synaptic nuclear envelope 1 Gene | ENSMUSG00000019769 | M400000330 |
| 34 | 3.338 | Rbm3 | RNA binding motif protein 3 Gene | ENSMUSG00000031167 | M400001343 |
| 35 | 3.301 | Fat1 | FAT tumor suppressor homolog 1 (Drosophila) Gene | ENSMUSG00000070047 | M400001394 |
| 36 | 3.281 | Als2 | amyotrophic lateral sclerosis 2 (juvenile) homolog (human) Gene | ENSMUSG00000026024 | M400000884 |
| 37 | 3.247 | Prelp | proline arginine-rich end leucine-rich repeat Gene | ENSMUSG00000041577 | M300013232 |
| 38 | 3.245 | Gpr171 | G protein-coupled receptor 171 Gene | ENSMUSG00000050075 | M300020786 |
| 39 | 3.207 | A2m | alpha-2-macroglobulin Gene | ENSMUSG00000030111 | M300007009 |
| 40 | 3.191 | Dnase1l2 | deoxyribonuclease 1-like 2 Gene | ENSMUSG00000024136 | M200006279 |
| 41 | 3.180 | Bcl6 | B-cell leukemia/lymphoma 6 Gene | ENSMUSG00000022508 | M200003401 |
| 42 | 3.117 | Nptx1 | neuronal pentraxin 1 Gene | ENSMUSG00000025582 | M400000844 |
| 43 | 3.114 | Abca2 | ATP-binding cassette, sub-family A (ABC1), member 2 Gene | ENSMUSG00000026944 | M200000837 |
| 44 | 3.067 | Fgf14 | fibroblast growth factor 14 Gene | ENSMUSG00000025551 | M400011615 |
| 45 | 3.065 | Mll1 | myeloid/lymphoid or mixed-lineage leukemia 1 Gene | ENSMUSG00000002028 | M200000897 |
| 46 | 3.038 | Spsb2 | splA/ryanodine receptor domain and SOCS box containing 2 Gene | ENSMUSG00000038451 | M200000999 |
| 47 | 3.034 | Pld5 | phospholipase D family, member 5 Gene | ENSMUSG00000055214 | M300019233 |
| 48 | 3.017 | Col7a1 | collagen, type VII, alpha 1 Gene | ENSMUSG00000025650 | M400000848 |
| 49 | 2.983 | Ptch1 | patched homolog 1 Gene | ENSMUSG00000021466 | M400014718 |
| 50 | 2.979 | Rutbc2 | small G protein signaling modulator 1 Gene | ENSMUSG00000042216 | M300013570 |
| 51 | 2.977 | Il20rb | interleukin 20 receptor beta Gene | ENSMUSG00000044244 | M400002870 |
| 52 | 2.965 | Snrk | SNF related kinase Gene | ENSMUSG00000038145 | M200012902 |
| 53 | 2.963 | Cgnl1 | cingulin-like 1 Gene | ENSMUSG00000032232 | M300008140 |
| 54 | 2.961 | Jup | junction plakoglobin Gene | ENSMUSG00000001552 | M200004269 |
| 55 | 2.957 | Clic6 | chloride intracellular channel 6 Gene | ENSMUSG00000022949 | M300003597 |
| 56 | 2.932 | Camkk2 | calcium/calmodulin-dependent protein kinase kinase 2, beta Gene | ENSMUSG00000029471 | M300006694 |
| 57 | 2.924 | Sorl1 | sortilin-related receptor, LDLR class A repeats-containing Gene | ENSMUSG00000049313 | M400003529 |
| 58 | 2.908 | Kcnt1 | potassium channel, subfamily T, member 1 Gene | ENSMUSG00000058740 | M300010355 |
| 59 | 2.900 | Nr1d1 | nuclear receptor subfamily 1, group D, member 1 Gene | ENSMUSG00000020889 | M200012251 |
| 60 | 2.892 | Dao1 | D-amino acid oxidase 1 Gene | ENSMUSG00000042096 | M300013502 |
| 61 | 2.884 | Ski | ski sarcoma viral oncogene homolog (avian) Gene | ENSMUSG00000029050 | M400013207 |
| 62 | 2.860 | Pcsk6 | proprotein convertase subtilisin/kexin type 6 Gene | ENSMUSG00000030513 | M200007288 |
| 63 | 2.860 | Rreb1 | ras responsive element binding protein 1 Gene | ENSMUSG00000039087 | M300011936 |
| 64 | 2.844 | Fos | FBJ osteosarcoma oncogene Gene | ENSMUSG00000021250 | M200002112 |
| 65 | 2.844 | Lrrk2 | leucine-rich repeat kinase 2 Gene | ENSMUSG00000036273 | M200007951 |
| 66 | 2.826 | 2810051F02Rik | RIKEN cDNA 2810051F02 gene Gene | ENSMUSG00000064070 | M400008537 |
| 67 | 2.813 | Gabpb2 | GA repeat binding protein, beta 2 Gene | ENSMUSG00000038766 | M400019263 |
| 68 | 2.809 | Ypel4 | yippee-like 4 (Drosophila) Gene | ENSMUSG00000034059 | M300009115 |
| 69 | 2.799 | Dusp1 | dual specificity phosphatase 1 Gene | ENSMUSG00000024190 | M200000904 |
| 70 | 2.780 | Adamts10 | a disintegrin-like and metallopeptidase (reprolysin type) with thrombospondin type 1 motif, 10 Gene | ENSMUSG00000024299 | M200006425 |
| 71 | 2.780 | Ndrg1 | N-myc downstream regulated gene 1 Gene | ENSMUSG00000005125 | M200001574 |
| 72 | 2.755 | AC153915.5-201 | Chloride channel protein, skeletal muscle (Chloride channel protein 1)(ClC-1) | ENSMUSG00000029862 | M300006914 |
| 73 | 2.745 | Usp2 | ubiquitin specific peptidase 2 Gene | ENSMUSG00000032010 | M200003187 |
| 74 | 2.738 | Car4 | carbonic anhydrase 4 Gene | ENSMUSG00000000805 | M200000659 |
| 75 | 2.738 | Slco4a1 | solute carrier organic anion transporter family, member 4a1 Gene | ENSMUSG00000038963 | M300011867 |
| 76 | 2.732 | Ak3l1 | adenylate kinase 3-like 1 Gene | ENSMUSG00000028527 | M200013852 |
| 77 | 2.725 | Tmem149 | transmembrane protein 149 Gene | ENSMUSG00000036826 | M200012954 |
| 78 | 2.723 | Hisppd2a | histidine acid phosphatase domain containing 2A Gene | ENSMUSG00000033526 | M300008817 |
| 79 | 2.704 | Kbtbd11 | kelch repeat and BTB (POZ) domain containing 11 Gene | ENSMUSG00000055675 | M400005258 |
| 80 | 2.696 | Slc7a5 | solute carrier family 7 (cationic amino acid transporter, y+ system), member 5 Gene | ENSMUSG00000040010 | M300012485 |
| 81 | 2.689 | Cdc14b | CDC14 cell division cycle 14 homolog B (S. cerevisiae) Gene | ENSMUSG00000033102 | M300008590 |
| 82 | 2.689 | Trak1 | trafficking protein, kinesin binding 1 Gene | ENSMUSG00000032536 | M400012281 |
| 83 | 2.672 | Egr1 | early growth response 1 Gene | ENSMUSG00000038418 | M200012044 |
| 84 | 2.668 | Foxo3 | forkhead box O3 Gene | ENSMUSG00000048756 | M400013007 |
| 85 | 2.657 | Ets2 | E26 avian leukemia oncogene 2, 3' domain Gene | ENSMUSG00000022895 | M200004369 |
| 86 | 2.654 | Nr4a1 | nuclear receptor subfamily 4, group A, member 1 Gene | ENSMUSG00000023034 | M200000038 |
| 87 | 2.654 | Zbtb4 | zinc finger and BTB domain containing 4 Gene | ENSMUSG00000018750 | M400014882 |
| 88 | 2.626 | Tardbp | TAR DNA binding protein Gene | ENSMUSG00000041459 | M400013039 |
| 89 | 2.623 | Mprip | myosin phosphatase Rho interacting protein Gene | ENSMUSG00000005417 | M400018372 |
| 90 | 2.619 | Ttc39b | tetratricopeptide repeat domain 39B Gene | ENSMUSG00000038172 | M400013639 |
| 91 | 2.617 | Golga4 | golgi autoantigen, golgin subfamily a, 4 Gene | ENSMUSG00000038708 | M400002181 |
| 92 | 2.603 | Dock9 | dedicator of cytokinesis 9 Gene | ENSMUSG00000025558 | M200004905 |
| 93 | 2.603 | Per3 |  | ENSMUSG00000028957 | M400013831 |
| 94 | 2.594 | Snrp70 | U1 small nuclear ribonucleoprotein polypeptide A Gene | ENSMUSG00000063511 | M200009365 |
| 95 | 2.592 | Tanc1 | tetratricopeptide repeat, ankyrin repeat and coiled-coil containing 1 Gene | ENSMUSG00000035168 | M200005751 |
| 96 | 2.585 | Ppfia1 | protein tyrosine phosphatase, receptor type, f polypeptide (PTPRF), interacting protein, alpha 1 Gene | ENSMUSG00000037519 | M300010975 |
| 97 | 2.578 | Tyro3 | TYRO3 protein tyrosine kinase 3 Gene | ENSMUSG00000027298 | M200001103 |
| 98 | 2.556 | 2900092E17Rik | RIKEN cDNA 2900092E17 gene Gene | ENSMUSG00000030680 | M200012040 |
| 99 | 2.539 | RP23-163J20.1 | Uncharacterized protein C20orf118 homolog | ENSMUSG00000074628 | M400002079 |
| 100 | 2.535 | 9130011E15Rik | RIKEN cDNA 9130011E15 gene Gene | ENSMUSG00000039901 | M300012429 |
| 101 | 2.533 | Rasl11b | RAS-like, family 11, member B Gene | ENSMUSG00000049907 | M200012063 |
| 102 | 2.518 | Fat2 | FAT tumor suppressor homolog 2 (Drosophila) Gene | ENSMUSG00000055333 | M400005130 |
| 103 | 2.516 | Per1 | period homolog 1 (Drosophila) Gene | ENSMUSG00000020893 | M200002536 |
| 104 | 2.511 | Tmem86b | transmembrane protein 86B Gene | ENSMUSG00000045282 | M300016251 |
| 105 | 2.507 | Odf2 | outer dense fiber of sperm tails 2 Gene | ENSMUSG00000026790 | M400008190 |
| 106 | 2.486 | Ltbp3 | latent transforming growth factor beta binding protein 3 Gene | ENSMUSG00000024940 | M400010834 |
| 107 | 2.481 | Socs7 | suppressor of cytokine signaling 4 Gene | ENSMUSG00000038485 | M400013219 |
| 108 | 2.467 | Hipk1 | homeodomain interacting protein kinase 1 Gene | ENSMUSG00000008730 | M200003942 |
| 109 | 2.466 | Opn3 | opsin 3 Gene | ENSMUSG00000026525 | M300005157 |
| 110 | 2.461 | Rnf123 | ring finger protein 123 Gene | ENSMUSG00000041528 | M200004426 |
| 111 | 2.452 | Mars | methionine-tRNA synthetase Gene | ENSMUSG00000040354 | M300012617 |
| 112 | 2.452 | Trpv4 | transient receptor potential cation channel, subfamily V, member 4 Gene | ENSMUSG00000014158 | M200016100 |
| 113 | 2.447 | Speg | SPEG complex locus Gene | ENSMUSG00000026207 | M200000801 |
| 114 | 2.437 | Ulk1 | Unc-51 like kinase 1 (C. elegans) Gene | ENSMUSG00000029512 | M200007519 |
| 115 | 2.428 | Atf7 | activating transcription factor 7 Gene | ENSMUSG00000071584 | M300017651 |
| 116 | 2.418 | Centd2 | ArfGAP with RhoGAP domain, ankyrin repeat and PH domain 1 Gene | ENSMUSG00000032812 | M200013595 |
| 117 | 2.413 | Bhlhb2 | basic helix-loop-helix domain containing, class B2 Gene | ENSMUSG00000030103 | M300007008 |
| 118 | 2.410 | Caln1 | calneuron 1 Gene | ENSMUSG00000060371 | M300004590 |
| 119 | 2.400 | Foxk1 | forkhead box K1 Gene | ENSMUSG00000056493 | M400017529 |
| 120 | 2.392 | Rgl3 | ral guanine nucleotide dissociation stimulator-like 3 Gene | ENSMUSG00000040146 | M200008777 |
| 121 | 2.390 | Lats2 | large tumor suppressor 2 Gene | ENSMUSG00000021959 | M400000530 |
| 122 | 2.373 | Hnrnpm | heterogeneous nuclear ribonucleoprotein M Gene | ENSMUSG00000059208 | M200009917 |
| 123 | 2.365 | BC033915 | Serine/threonine-protein kinase QSK (EC 2.7.11.1)(Salt-inducible kinase 3)(SIK-3)(SIK3) | ENSMUSG00000034135 | M400011605 |
| 124 | 2.357 | Zfyve26 | zinc finger, FYVE domain containing 26 Gene | ENSMUSG00000066440 | M200006048 |
| 125 | 2.351 | Safb2 | scaffold attachment factor B2 Gene | ENSMUSG00000042625 | M300013793 |
| 126 | 2.347 | Enpp2 | ectonucleotide pyrophosphatase/phosphodiesterase 2 Gene | ENSMUSG00000022425 | M200005834 |
| 127 | 2.339 | Nab2 | Ngfi-A binding protein 2 Gene | ENSMUSG00000025402 | M400010850 |
| 128 | 2.336 | AC155937.4-202 | Putative uncharacterized proteinNfic protein ; | ENSMUSG00000079936 | M300002080 |
| 129 | 2.336 | BC030863 | Uncharacterized protein KIAA0819 | ENSMUSG00000051586 | M300022206 |
| 130 | 2.326 | Abcg1 | ATP-binding cassette, sub-family G (WHITE), member 1 Gene | ENSMUSG00000024030 | M200003392 |
| 131 | 2.323 | Dennd4b | DENN/MADD domain containing 4B Gene | ENSMUSG00000042404 | M400002621 |
| 132 | 2.321 | Svep1 | sushi, von Willebrand factor type A, EGF and pentraxin domain containing 1 Gene | ENSMUSG00000028369 | M200003816 |
| 133 | 2.320 | AC159283.2 | Putative uncharacterized protein | ENSMUSG00000079179 | M200004959 |
| 134 | 2.309 | Lnx2 | ligand of numb-protein X 2 Gene | ENSMUSG00000016520 | M200007585 |
| 135 | 2.307 | Idua | iduronidase, alpha-L- Gene | ENSMUSG00000033540 | M200001165 |
| 136 | 2.302 | Abca7 | ATP-binding cassette, sub-family A (ABC1), member 7 Gene | ENSMUSG00000035722 | M200008508 |
| 137 | 2.302 | Stat2 | signal transducer and activator of transcription 2 Gene | ENSMUSG00000040033 | M200004256 |
| 138 | 2.299 | A230083H22Rik | BNIP2 motif-containing molecule at the C-terminal region 1 | ENSMUSG00000039126 | M300011972 |
| 139 | 2.294 | Nrl | neural retina leucine zipper gene Gene | ENSMUSG00000040632 | M300012774 |
| 140 | 2.285 | 1110012J17Rik | RIKEN cDNA 1110012J17 gene Gene | ENSMUSG00000052105 | M400010146 |
| 141 | 2.269 | Dnm1 | dynamin 1 Gene | ENSMUSG00000026825 | M300005308 |
| 142 | 2.267 | Slfn10 | schlafen 10 Gene | ENSMUSG00000072621 | M300018375 |
| 143 | 2.264 | Adamtsl4 | ADAMTS-like 4 Gene | ENSMUSG00000015850 | M200014222 |
| 144 | 2.261 | Slc41a3 | solute carrier family 41, member 3 Gene | ENSMUSG00000030089 | M200007299 |
| 145 | 2.256 | Itpr3 | inositol 1,4,5-triphosphate receptor 3 Gene | ENSMUSG00000042644 | M200005377 |
| 146 | 2.256 | Whsc1l1 | Wolf-Hirschhorn syndrome candidate 1-like 1 (human) Gene | ENSMUSG00000054823 | M400015692 |
| 147 | 2.255 | 2210010N04Rik | RIKEN cDNA 2210010N04 gene Gene | ENSMUSG00000066621 | M400013367 |
| 148 | 2.255 | Ccdc45 | coiled-coil domain containing 45 Gene | ENSMUSG00000018372 | M300001661 |
| 149 | 2.255 | Dusp16 | dual specificity phosphatase 16 Gene | ENSMUSG00000030203 | M400001236 |
| 150 | 2.255 | Helz | helicase with zinc finger domain Gene | ENSMUSG00000020721 | M400013967 |
| 151 | 2.247 | Dlg1 | discs, large homolog 1 (Drosophila) Gene | ENSMUSG00000022770 | M300003491 |
| 152 | 2.247 | Macf1 | microtubule-actin crosslinking factor 1 Gene | ENSMUSG00000028649 | M400001104 |
| 153 | 2.244 | Dst | dystonin Gene | ENSMUSG00000026131 | M400011820 |
| 154 | 2.241 | Tnrc6c | trinucleotide repeat containing 6C Gene | ENSMUSG00000025571 | M200008262 |
| 155 | 2.238 | Ahcyl2 | S-adenosylhomocysteine hydrolase-like 2 Gene | ENSMUSG00000029772 | M300006859 |
| 156 | 2.228 | Nrxn3 | neurexin III Gene | ENSMUSG00000066392 | M300016334 |
| 157 | 2.225 | St5 | suppression of tumorigenicity 5 Gene | ENSMUSG00000031024 | M200000392 |
| 158 | 2.219 | Ptpn21 | protein tyrosine phosphatase, non-receptor type 21 Gene | ENSMUSG00000021009 | M300002509 |
| 159 | 2.218 | 3110001A13Rik | family with sequence similarity 107, member B Gene | ENSMUSG00000026655 | M200012921 |
| 160 | 2.207 | Pqlc3 | PQ loop repeat containing Gene | ENSMUSG00000045679 | M300016615 |
| 161 | 2.198 | Mtap4 | microtubule-associated protein 4 Gene | ENSMUSG00000032479 | M200003107 |
| 162 | 2.196 | RP23-455B1.3 | Likely ortholog of H. sapiens chromosome 9 open reading frame 5 (C9orf5) | ENSMUSG00000055296 | M400016348 |
| 163 | 2.192 | 1190002N15Rik | RIKEN cDNA 1190002N15 gene Gene | ENSMUSG00000045414 | M400013036 |
| 164 | 2.190 | Cited2 | Cbp/p300-interacting transactivator, with Glu/Asp-rich carboxy-terminal domain, 2 Gene | ENSMUSG00000039910 | M300012438 |
| 165 | 2.187 | Slc6a8 | solute carrier family 6 (neurotransmitter transporter, creatine), member 8 Gene | ENSMUSG00000019558 | M200013579 |
| 166 | 2.181 | Chn2 | chimerin (chimaerin) 2 Gene | ENSMUSG00000004633 | M300000581 |
| 167 | 2.180 | Acrbp | proacrosin binding protein Gene | ENSMUSG00000072770 | M200001579 |
| 168 | 2.178 | Foxn3 | forkhead box N3 Gene | ENSMUSG00000044661 | M400002923 |
| 169 | 2.173 | Car9 | carbonic anhydrase 9 Gene | ENSMUSG00000028463 | M200014286 |
| 170 | 2.169 | Fbxo45 | F-box protein 45 Gene | ENSMUSG00000035764 | M400014371 |
| 171 | 2.167 | Aox1 | aldehyde oxidase 1 Gene | ENSMUSG00000063558 | M200005350 |
| 172 | 2.163 | Plekha5 | pleckstrin homology domain containing, family A member 5 Gene | ENSMUSG00000030231 | M300007090 |
| 173 | 2.157 | Whrn | whirlin Gene | ENSMUSG00000039137 | M300011980 |
| 174 | 2.154 | Sipa1l3 | signal-induced proliferation-associated 1 like 3 Gene | ENSMUSG00000030583 | M200004625 |
| 175 | 2.151 | Cit | citron Gene | ENSMUSG00000029516 | M400016033 |
| 176 | 2.145 | Mfsd4 | major facilitator superfamily domain containing 4 Gene | ENSMUSG00000059149 | M400008673 |
| 177 | 2.145 | Spag1 | sperm associated antigen 1 Gene | ENSMUSG00000037617 | M200015401 |
| 178 | 2.144 | Olfr453 | olfactory receptor 453 Gene | ENSMUSG00000048504 | M400003429 |
| 179 | 2.144 | Trim62 | tripartite motif-containing 62 Gene | ENSMUSG00000041000 | M400002465 |
| 180 | 2.139 | Pptc7 | PTC7 protein phosphatase homolog (S. cerevisiae) Gene | ENSMUSG00000038582 | M300011640 |
| 181 | 2.135 | Tpcn2 | two pore segment channel 2 Gene | ENSMUSG00000048677 | M300009917 |
| 182 | 2.133 | Atp1a1 | ATPase, Na+/K+ transporting, alpha 1 polypeptide Gene | ENSMUSG00000033161 | M200013073 |
| 183 | 2.123 | Aff1 | AF4/FMR2 family, member 1 Gene | ENSMUSG00000029313 | M400002694 |
| 184 | 2.123 | Igf1r | insulin-like growth factor I receptor Gene | ENSMUSG00000005533 | M400014887 |
| 185 | 2.118 | Xkr6 | X Kell blood group precursor related family member 6 homolog Gene | ENSMUSG00000035067 | M300009679 |
| 186 | 2.117 | Myo15 | myosin XV Gene | ENSMUSG00000042678 | M200016050 |
| 187 | 2.116 | Traf1 | Tnf receptor-associated factor 1 Gene | ENSMUSG00000026875 | M200003178 |
| 188 | 2.105 | Usp28 | ubiquitin specific peptidase 28 Gene | ENSMUSG00000032267 | M300008156 |
| 189 | 2.104 | Ift172 | intraflagellar transport 172 homolog (Chlamydomonas) Gene | ENSMUSG00000038564 | M300006519 |
| 190 | 2.104 | Zmym1 | zinc finger, MYM domain containing 1 Gene | ENSMUSG00000043872 | M200015559 |
| 191 | 2.094 | Ccdc120 | coiled-coil domain containing 120 Gene | ENSMUSG00000031150 | M300007582 |
| 192 | 2.094 | Plekhf1 | pleckstrin homology domain containing, family F (with FYVE domain) member 1 Gene | ENSMUSG00000074170 | M200007018 |
| 193 | 2.092 | Eml3 | echinoderm microtubule associated protein like 3 Gene | ENSMUSG00000071647 | M200007041 |
| 194 | 2.092 | Epb4.1 | erythrocyte protein band 4.1 Gene | ENSMUSG00000028906 | M200006797 |
| 195 | 2.091 | Centg1 | ArfGAP with GTPase domain, ankyrin repeat and PH domain 2 Gene | ENSMUSG00000025422 | M400000829 |
| 196 | 2.086 | Gls2 | glutaminase 2 (liver, mitochondrial) Gene | ENSMUSG00000044005 | M400002835 |
| 197 | 2.086 | Pde1c | phosphodiesterase 1C Gene | ENSMUSG00000004347 | M300000548 |
| 198 | 2.082 | Pou2f1 | POU domain, class 2, transcription factor 1 Gene | ENSMUSG00000026565 | M400009801 |
| 199 | 2.076 | 2810046L04Rik | RIKEN cDNA 2810046L04 gene Gene | ENSMUSG00000049504 | M400012264 |
| 200 | 2.076 | Tmem62 | transmembrane protein 62 Gene | ENSMUSG00000054484 | M400004826 |
| 201 | 2.071 | D430015B01Rik | family with sequence similarity 13, member A Gene | ENSMUSG00000037709 | M300011086 |
| 202 | 2.065 | Dgkh | diacylglycerol kinase, eta Gene | ENSMUSG00000034731 | M400001732 |
| 203 | 2.061 | Pacsin3 | protein kinase C and casein kinase substrate in neurons 3 Gene | ENSMUSG00000027257 | M200004608 |
| 204 | 2.059 | Ece2 | endothelin converting enzyme 2 Gene | ENSMUSG00000022842 | M400011878 |
| 205 | 2.059 | Olfr938 | olfactory receptor 938 Gene | ENSMUSG00000048501 | M400003428 |
| 206 | 2.059 | Pom121 | nuclear pore membrane protein 121 Gene | ENSMUSG00000053293 | M400004416 |
| 207 | 2.058 | Plagl2 | pleiomorphic adenoma gene-like 2 Gene | ENSMUSG00000051413 | M400011297 |
| 208 | 2.058 | Rai16 | family with sequence similarity 160, member B2 Gene | ENSMUSG00000022095 | M300003088 |
| 209 | 2.056 | Cpt1b | carnitine palmitoyltransferase 1b, muscle Gene | ENSMUSG00000078937 | M400000591 |
| 210 | 2.043 | Cds1 | CDP-diacylglycerol synthase 1 Gene | ENSMUSG00000029330 | M200014600 |
| 211 | 2.042 | Inpp5a | inositol polyphosphate-5-phosphatase A Gene | ENSMUSG00000025477 | M300004653 |
| 212 | 2.041 | Pan2 | PAN2 polyA specific ribonuclease subunit homolog (S. cerevisiae) Gene | ENSMUSG00000005682 | M200007240 |
| 213 | 2.032 | D330001F17Rik | RIKEN cDNA D330001F17 gene Gene | ENSMUSG00000022558 | M400004995 |
| 214 | 2.031 | Gpc6 | glypican 6 Gene | ENSMUSG00000058571 | M400014875 |
| 215 | 2.031 | Mfhas1 | malignant fibrous histiocytoma amplified sequence 1 Gene | ENSMUSG00000070056 | M300011774 |
| 216 | 2.031 | Slc20a2 | solute carrier family 20, member 2 Gene | ENSMUSG00000037656 | M200009931 |
| 217 | 2.029 | Entpd4 | ectonucleoside triphosphate diphosphohydrolase 4 Gene | ENSMUSG00000022066 | M400011543 |
| 218 | 2.021 | Tyk2 | tyrosine kinase 2 Gene | ENSMUSG00000032175 | M400001433 |
| 219 | 2.020 | Mycbp2 | MYC binding protein 2 Gene | ENSMUSG00000033004 | M300008551 |
| 220 | 2.018 | Pcmtd2 | protein-L-isoaspartate (D-aspartate) O-methyltransferase domain containing 2 Gene | ENSMUSG00000027589 | M300005710 |
| 221 | 2.017 | C430014K11Rik | RIKEN cDNA C430014K11 gene Gene | ENSMUSG00000069564 | M400015609 |
| 222 | 2.015 | Mpdz | multiple PDZ domain protein Gene | ENSMUSG00000028402 | M300006078 |
| 223 | 2.014 | Smarcd2 | SWI/SNF related, matrix associated, actin dependent regulator of chromatin, subfamily d, member 2 Gene | ENSMUSG00000078619 | M400000411 |
| 224 | 2.011 | Btbd14a | nucleus accumbens associated 2, BEN and BTB (POZ) domain containing Gene | ENSMUSG00000026932 | M400012979 |
| 225 | 2.008 | Rora | RAR-related orphan receptor alpha Gene | ENSMUSG00000032238 | M400001441 |
| 226 | 2.007 | Boc | biregional cell adhesion molecule-related/down-regulated by oncogenes (Cdon) binding protein Gene | ENSMUSG00000022687 | M200013652 |
| 227 | 2.006 | AL604063.4 | Putative uncharacterized protein | ENSMUSG00000055697 | M400005267 |
| 228 | 2.003 | Alms1 | Alstrom syndrome 1 homolog (human) Gene | ENSMUSG00000063810 | M400008426 |
| 229 | 2.000 | Smpd4 | sphingomyelin phosphodiesterase 4 Gene | ENSMUSG00000005899 | M200007629 |
| 230 | 0.500 | Bloc1s1 | biogenesis of lysosome-related organelles complex-1, subunit 1 Gene | ENSMUSG00000025349 | M400000823 |
| 231 | 0.500 | Phf6 | PHD finger protein 6 Gene | ENSMUSG00000025626 | M200005362 |
| 232 | 0.500 | Nsmce1 | non-SMC element 1 homolog (S. cerevisiae) Gene | ENSMUSG00000030750 | M300007402 |
| 233 | 0.500 | Ssu72 | Ssu72 RNA polymerase II CTD phosphatase homolog (yeast) Gene | ENSMUSG00000029038 | M400011586 |
| 234 | 0.500 | Tmem121 | transmembrane protein 121 Gene | ENSMUSG00000049036 | M200009116 |
| 235 | 0.499 | 0610010K14Rik | RIKEN cDNA 0610010K14 gene Gene | ENSMUSG00000020831 | M300002418 |
| 236 | 0.499 | 1700025K23Rik | RIKEN cDNA 1700025K23 gene Gene | ENSMUSG00000051736 | M400012548 |
| 237 | 0.499 | 2610027C15Rik | family with sequence similarity 176, member B Gene | ENSMUSG00000050212 | M300020921 |
| 238 | 0.499 | AC105336.14 | UDP-N-acetylglucosamine transferase subunit ALG14 homolog | ENSMUSG00000039887 | M400002307 |
| 239 | 0.499 | Vps29 | vacuolar protein sorting 29 (S. pombe) Gene | ENSMUSG00000029462 | M200005961 |
| 240 | 0.498 | Def6 | differentially expressed in FDCP 6 Gene | ENSMUSG00000002257 | M200015055 |
| 241 | 0.498 | Mrps30 | mitochondrial ribosomal protein S30 Gene | ENSMUSG00000021731 | M200009486 |
| 242 | 0.498 | Polr2f | polymerase (RNA) II (DNA directed) polypeptide F Gene | ENSMUSG00000033020 | M200004328 |
| 243 | 0.498 | Deaf1 | deformed epidermal autoregulatory factor 1 (Drosophila) Gene | ENSMUSG00000058886 | M200005980 |
| 244 | 0.498 | Mcee | methylmalonyl CoA epimerase Gene | ENSMUSG00000033429 | M200002860 |
| 245 | 0.498 | Nanos1 | nanos homolog 1 (Drosophila) Gene | ENSMUSG00000072437 | M400012479 |
| 246 | 0.498 | Csk | c-src tyrosine kinase Gene | ENSMUSG00000032312 | M300008184 |
| 247 | 0.498 | Tuba1b | tubulin, alpha 1B Gene | ENSMUSG00000023004 | M300015433 |
| 248 | 0.497 | D2Bwg1335e | DNL-type zinc finger protein | ENSMUSG00000075467 | M300005386 |
| 249 | 0.497 | Igbp1 | immunoglobulin (CD79A) binding protein 1 Gene | ENSMUSG00000031221 | M400001348 |
| 250 | 0.497 | Leprot | leptin receptor overlapping transcript Gene | ENSMUSG00000035212 | M300006142 |
| 251 | 0.497 | Ppap2c | phosphatidic acid phosphatase type 2c Gene | ENSMUSG00000052151 | M300002134 |
| 252 | 0.497 | Clic1 | chloride intracellular channel 1 Gene | ENSMUSG00000007041 | M200006521 |
| 253 | 0.497 | Pten | phosphatase and tensin homolog Gene | ENSMUSG00000013663 | M200000642 |
| 254 | 0.497 | Rab11a | RAB11a, member RAS oncogene family Gene | ENSMUSG00000004771 | M200000553 |
| 255 | 0.497 | Sh3bp4 | SH3-domain binding protein 4 Gene | ENSMUSG00000036206 | M400011817 |
| 256 | 0.497 | Bcat1 | branched chain aminotransferase 1, cytosolic Gene | ENSMUSG00000030268 | M400010737 |
| 257 | 0.496 | Cd200 | CD200 antigen Gene | ENSMUSG00000022661 | M400011073 |
| 258 | 0.496 | Mrps18b | mitochondrial ribosomal protein S18B Gene | ENSMUSG00000024436 | M200006503 |
| 259 | 0.496 | Rad51 | RAD51 homolog (S. cerevisiae) Gene | ENSMUSG00000027323 | M200000087 |
| 260 | 0.495 | Peli1 | pellino 1 Gene | ENSMUSG00000020134 | M200006248 |
| 261 | 0.494 | Gfra2 | glial cell line derived neurotrophic factor family receptor alpha 2 Gene | ENSMUSG00000022103 | M200013774 |
| 262 | 0.494 | Alcam | activated leukocyte cell adhesion molecule Gene | ENSMUSG00000022636 | M300003422 |
| 263 | 0.494 | Cxcl12 | chemokine (C-X-C motif) ligand 12 Gene | ENSMUSG00000061353 | M400011403 |
| 264 | 0.494 | Rgs11 | regulator of G-protein signaling 11 Gene | ENSMUSG00000024186 | M200003232 |
| 265 | 0.494 | Tbc1d12 | TBC1D12: TBC1 domain family, member 12 Gene | ENSMUSG00000048720 | M300004420 |
| 266 | 0.494 | Chst1 | carbohydrate (keratan sulfate Gal-6) sulfotransferase 1 Gene | ENSMUSG00000027221 | M200008003 |
| 267 | 0.494 | Srd5a3 |  | ENSMUSG00000029233 | M300006564 |
| 268 | 0.493 | Pcdh7 | protocadherin 7 Gene | ENSMUSG00000029108 | M400001141 |
| 269 | 0.493 | 2410018M08Rik | RIKEN cDNA 2410018M08 gene Gene | ENSMUSG00000034173 | M200012574 |
| 270 | 0.493 | Mrpl20 | mitochondrial ribosomal protein L20 Gene | ENSMUSG00000029066 | M300006462 |
| 271 | 0.493 | Psmg4 | proteasome (prosome, macropain) assembly chaperone 4 Gene | ENSMUSG00000071451 | M300021401 |
| 272 | 0.493 | Mrpl36 | mitochondrial ribosomal protein L36 Gene | ENSMUSG00000021607 | M200005345 |
| 273 | 0.493 | Psma6 | proteasome (prosome, macropain) subunit, alpha type 6 Gene | ENSMUSG00000021024 | M300002517 |
| 274 | 0.493 | Sh3yl1 | Sh3 domain YSC-like 1 Gene | ENSMUSG00000020669 | M200013491 |
| 275 | 0.493 | Tmepai | prostate transmembrane protein, androgen induced 1 Gene | ENSMUSG00000038400 | M400011417 |
| 276 | 0.493 | Tsc22d1 | TSC22 domain family, member 1 Gene | ENSMUSG00000022010 | M200004008 |
| 277 | 0.492 | Bud31 | BUD31 homolog (yeast) Gene | ENSMUSG00000038722 | M400002183 |
| 278 | 0.492 | Gamt | guanidinoacetate methyltransferase Gene | ENSMUSG00000020150 | M200002525 |
| 279 | 0.492 | Ywhaq | tyrosine 3-monooxygenase/tryptophan 5-monooxygenase activation protein, theta polypeptide Gene | ENSMUSG00000076432 | M400011166 |
| 280 | 0.492 | Zfp637 | zinc finger protein 637 Gene | ENSMUSG00000059689 | M300013504 |
| 281 | 0.492 | 2410018G20Rik | family with sequence similarity 128, member B Gene | ENSMUSG00000022671 | M300003446 |
| 282 | 0.491 | Grinl1a | glutamate receptor, ionotropic, N-methyl D-aspartate-like 1A Gene | ENSMUSG00000032199 | M200004052 |
| 283 | 0.491 | OTTMUSG00000004461 | predicted gene, OTTMUSG00000004461 Gene | ENSMUSG00000055963 | M400005362 |
| 284 | 0.491 | AL808128.4 |  | ENSMUSG00000082044 | M400010908 |
| 285 | 0.491 | Ndufa2 | NADH dehydrogenase (ubiquinone) 1 alpha subcomplex, 2 Gene | ENSMUSG00000014294 | M300001272 |
| 286 | 0.490 | 1500032D16Rik | NADH dehydrogenase [ubiquinone] flavoprotein 3, mitochondrial Precursor (NADH-ubiquinone oxidoreductase 9 kDa subunit) | ENSMUSG00000024038 | M400011695 |
| 287 | 0.490 | 1810030N24Rik | RIKEN cDNA 1810030N24 gene Gene | ENSMUSG00000028295 | M200005821 |
| 288 | 0.490 | Emp2 | epithelial membrane protein 2 Gene | ENSMUSG00000022505 | M400010782 |
| 289 | 0.490 | Tfpi | tissue factor pathway inhibitor Gene | ENSMUSG00000027082 | M300005466 |
| 290 | 0.489 | Cd24a | CD24a antigen Gene | ENSMUSG00000047139 | M300017999 |
| 291 | 0.489 | Uchl3 | ubiquitin carboxyl-terminal esterase L3 (ubiquitin thiolesterase) Gene | ENSMUSG00000022111 | M400000540 |
| 292 | 0.489 | Tspan12 | tetraspanin 12 Gene | ENSMUSG00000029669 | M300006804 |
| 293 | 0.489 | 2310005N01Rik | RIKEN cDNA 2310005N01 gene Gene | ENSMUSG00000028863 | M300006336 |
| 294 | 0.489 | Ensa | endosulfine alpha Gene | ENSMUSG00000038619 | M400011316 |
| 295 | 0.488 | Clasp1 | CLIP associating protein 1 Gene | ENSMUSG00000064302 | M300005067 |
| 296 | 0.488 | Hccs | holocytochrome c synthetase Gene | ENSMUSG00000031352 | M200002845 |
| 297 | 0.488 | Psmd13 | proteasome (prosome, macropain) 26S subunit, non-ATPase, 13 Gene | ENSMUSG00000025487 | M200006641 |
| 298 | 0.488 | Tcp1 | t-complex protein 1 Gene | ENSMUSG00000068039 | M400011215 |
| 299 | 0.488 | A230051G13Rik | RIKEN cDNA A230051G13 gene Gene | ENSMUSG00000049287 | M300020030 |
| 300 | 0.488 | AC142244.11 | Uridine-cytidine kinase 2 (UCK 2)(EC 2.7.1.48)(Uridine monophosphokinase 2)(Cytidine monophosphokinase 2) | ENSMUSG00000026558 | M200005101 |
| 301 | 0.487 | Actr10 | ARP10 actin-related protein 10 homolog (S. cerevisiae) Gene | ENSMUSG00000021076 | M200006427 |
| 302 | 0.487 | Fbxo25 | F-box protein 25 Gene | ENSMUSG00000038365 | M400002121 |
| 303 | 0.487 | Nme1 | non-metastatic cells 1, protein (NM23A) expressed in Gene | ENSMUSG00000037601 | M300011019 |
| 304 | 0.487 | Atp6v0d1 | ATPase, H transporting, lysosomal V0 subunit D1 Gene | ENSMUSG00000013160 | M200000393 |
| 305 | 0.487 | Psmb5 | proteasome (prosome, macropain) subunit, beta type 5 Gene | ENSMUSG00000022193 | M300003132 |
| 306 | 0.486 | 6530401N04Rik | RIKEN cDNA 6530401N04 gene Gene | ENSMUSG00000020956 | M300002485 |
| 307 | 0.486 | Exosc1 | exosome component 1 Gene | ENSMUSG00000034321 | M300009281 |
| 308 | 0.486 | Pdgfa | platelet derived growth factor, alpha Gene | ENSMUSG00000025856 | M400010861 |
| 309 | 0.486 | Set | SET translocation Gene | ENSMUSG00000054766 | M400004935 |
| 310 | 0.486 | Slc25a5 | solute carrier family 25 (mitochondrial carrier, adenine nucleotide translocator), member 5 Gene | ENSMUSG00000016319 | M400000252 |
| 311 | 0.485 | Eif4e2 | eukaryotic translation initiation factor 4E member 2 Gene | ENSMUSG00000026254 | M300005004 |
| 312 | 0.485 | Sirt4 | sirtuin 4 (silent mating type information regulation 2 homolog) 4 (S. cerevisiae) Gene | ENSMUSG00000029524 | M200008673 |
| 313 | 0.485 | Tagln2 | transgelin 2 Gene | ENSMUSG00000026547 | M400000941 |
| 314 | 0.485 | Ppap2a | phosphatidic acid phosphatase 2a Gene | ENSMUSG00000021759 | M300002937 |
| 315 | 0.485 | Cisd1 | CDGSH iron sulfur domain 1 Gene | ENSMUSG00000037710 | M200006286 |
| 316 | 0.485 | Uqcrh | ubiquinol-cytochrome c reductase hinge protein Gene | ENSMUSG00000063882 | M400008456 |
| 317 | 0.484 | Mrps24 | mitochondrial ribosomal protein S24 Gene | ENSMUSG00000020477 | M200002524 |
| 318 | 0.484 | Fdft1 | farnesyl diphosphate farnesyl transferase 1 Gene | ENSMUSG00000021273 | M300009717 |
| 319 | 0.484 | Fem1b | feminization 1 homolog b (C. elegans) Gene | ENSMUSG00000032244 | M200004007 |
| 320 | 0.484 | Mtap1b | microtubule-associated protein 1B Gene | ENSMUSG00000052727 | M200001684 |
| 321 | 0.484 | Polr2j | polymerase (RNA) II (DNA directed) polypeptide J Gene | ENSMUSG00000039771 | M200002020 |
| 322 | 0.484 | D2Ertd750e | predicted gene, OTTMUSG00000015636 Gene | ENSMUSG00000027331 | M200003045 |
| 323 | 0.484 | Glrx3 | glutaredoxin 3 Gene | ENSMUSG00000031068 | M300007546 |
| 324 | 0.484 | Pxmp4 | peroxisomal membrane protein 4 Gene | ENSMUSG00000000876 | M200004970 |
| 325 | 0.484 | Tacc3 | transforming, acidic coiled-coil containing protein 3 Gene | ENSMUSG00000037313 | M200005723 |
| 326 | 0.483 | E2f2 | E2F transcription factor 2 Gene | ENSMUSG00000018983 | M300001757 |
| 327 | 0.483 | Plk2 | polo-like kinase 2 (Drosophila) Gene | ENSMUSG00000021701 | M200000136 |
| 328 | 0.483 | Snx4 | sorting nexin 4 Gene | ENSMUSG00000022808 | M200005877 |
| 329 | 0.483 | Rps11 | ribosomal protein S11 Gene | ENSMUSG00000003429 | M400011218 |
| 330 | 0.483 | Snx16 | sorting nexin 16 Gene | ENSMUSG00000027534 | M200005652 |
| 331 | 0.483 | Cmpk1 | cytidine monophosphate (UMP-CMP) kinase 1 Gene | ENSMUSG00000028719 | M200006649 |
| 332 | 0.483 | Tmem128 |  | ENSMUSG00000067365 | M400011488 |
| 333 | 0.482 | Rraga | Ras-related GTP binding A Gene | ENSMUSG00000070934 | M300018457 |
| 334 | 0.482 | Uqcrc2 | ubiquinol cytochrome c reductase core protein 2 Gene | ENSMUSG00000030884 | M300007481 |
| 335 | 0.482 | Znrf2 | zinc and ring finger 2 Gene | ENSMUSG00000058446 | M400006347 |
| 336 | 0.482 | Mtx2 | metaxin 2 Gene | ENSMUSG00000027099 | M200012105 |
| 337 | 0.482 | Ndufb7 | NADH dehydrogenase (ubiquinone) 1 beta subcomplex, 7 Gene | ENSMUSG00000033938 | M200006599 |
| 338 | 0.481 | 2510006D16Rik | RIKEN cDNA 2510006D16 gene Gene | ENSMUSG00000028797 | M400009305 |
| 339 | 0.481 | Asf1b | ASF1 anti-silencing function 1 homolog B (S. cerevisiae) Gene | ENSMUSG00000005470 | M200006598 |
| 340 | 0.481 | Med27 | mediator complex subunit 27 Gene | ENSMUSG00000026799 | M300005291 |
| 341 | 0.481 | Mfap2 | microfibrillar-associated protein 2 Gene | ENSMUSG00000060572 | M200002538 |
| 342 | 0.481 | Praf2 | PRA1 domain family 2 Gene | ENSMUSG00000031149 | M200009302 |
| 343 | 0.481 | Dpy30 | dpy-30 homolog (C. elegans) Gene | ENSMUSG00000024067 | M400011462 |
| 344 | 0.481 | Guk1 | guanylate kinase 1 Gene | ENSMUSG00000020444 | M300002194 |
| 345 | 0.481 | Tkt | transketolase Gene | ENSMUSG00000021957 | M200009754 |
| 346 | 0.481 | Trappc1 | trafficking protein particle complex 1 Gene | ENSMUSG00000049299 | M400003528 |
| 347 | 0.480 | Pno1 | partner of NOB1 homolog (S. cerevisiae) Gene | ENSMUSG00000020116 | M200005719 |
| 348 | 0.480 | 5430435G22Rik | RIKEN cDNA 5430435G22 gene Gene | ENSMUSG00000052688 | M400011899 |
| 349 | 0.480 | Sec13 | SEC13 homolog (S. cerevisiae) Gene | ENSMUSG00000030298 | M200006422 |
| 350 | 0.479 | Psmd6 | proteasome (prosome, macropain) 26S subunit, non-ATPase, 6 Gene | ENSMUSG00000021737 | M200005618 |
| 351 | 0.479 | Mrps28 | mitochondrial ribosomal protein S28 Gene | ENSMUSG00000040269 | M200008075 |
| 352 | 0.479 | Pgp | phosphoglycolate phosphatase Gene | ENSMUSG00000043445 | M400002754 |
| 353 | 0.479 | Syngr1 | synaptogyrin 1 Gene | ENSMUSG00000022415 | M300003269 |
| 354 | 0.478 | CT009518.6-201 |  | ENSMUSG00000051723 | M400005915 |
| 355 | 0.478 | Foxm1 | forkhead box M1 Gene | ENSMUSG00000001517 | M200013895 |
| 356 | 0.477 | Ets1 | E26 avian leukemia oncogene 1, 5' domain Gene | ENSMUSG00000032035 | M300008034 |
| 357 | 0.477 | Mad2l1bp | MAD2L1 binding protein Gene | ENSMUSG00000034509 | M200005697 |
| 358 | 0.477 | Pbx3 | pre B-cell leukemia transcription factor 3 Gene | ENSMUSG00000038718 | M200002526 |
| 359 | 0.476 | 2700081O15Rik | RIKEN cDNA 2700081O15 gene Gene | ENSMUSG00000053080 | M400004331 |
| 360 | 0.476 | Hadh | hydroxyacyl-Coenzyme A dehydrogenase Gene | ENSMUSG00000027984 | M200000939 |
| 361 | 0.476 | Nrp1 | neuropilin 1 Gene | ENSMUSG00000025810 | M300004780 |
| 362 | 0.475 | BC028528 | Uncharacterized protein C1orf54 homolog Precursor (Protein L259) | ENSMUSG00000038543 | M300011618 |
| 363 | 0.475 | Cdh11 | cadherin 11 Gene | ENSMUSG00000031673 | M200001793 |
| 364 | 0.475 | Pacs2 | phosphofurin acidic cluster sorting protein 2 Gene | ENSMUSG00000021143 | M300002585 |
| 365 | 0.475 | Aipl1 | aryl hydrocarbon receptor-interacting protein-like 1 Gene | ENSMUSG00000040554 | M300012724 |
| 366 | 0.474 | A230106M20Rik | RIKEN cDNA A230106M20 gene Gene | ENSMUSG00000013367 | M300001219 |
| 367 | 0.474 | Eif3eip | eukaryotic translation initiation factor 3, subunit E interacting protein Gene | ENSMUSG00000033047 | M200013087 |
| 368 | 0.474 | Psmb4 | proteasome (prosome, macropain) subunit, beta type 4 Gene | ENSMUSG00000005779 | M200000129 |
| 369 | 0.474 | AC134441.4 |  | ENSMUSG00000057157 | M400005791 |
| 370 | 0.474 | Wdr18 | WD repeat domain 18 Gene | ENSMUSG00000035754 | M300010076 |
| 371 | 0.473 | Aprt | adenine phosphoribosyl transferase Gene | ENSMUSG00000006589 | M400010967 |
| 372 | 0.473 | Hdac2 | histone deacetylase 2 Gene | ENSMUSG00000019777 | M400000332 |
| 373 | 0.473 | Mdh2 | malate dehydrogenase 2, NAD (mitochondrial) Gene | ENSMUSG00000019179 | M200004205 |
| 374 | 0.473 | Rcn2 | reticulocalbin 2 Gene | ENSMUSG00000032320 | M300008186 |
| 375 | 0.473 | 2810428I15Rik | RIKEN cDNA 2810428I15 gene Gene | ENSMUSG00000058833 | M200005904 |
| 376 | 0.473 | Atxn10 | ataxin 10 Gene | ENSMUSG00000016541 | M200001593 |
| 377 | 0.473 | Bhlhb9 | basic helix-loop-helix domain containing, class B9 Gene | ENSMUSG00000072964 | M400005113 |
| 378 | 0.472 | Map1lc3a | microtubule-associated protein 1 light chain 3 alpha Gene | ENSMUSG00000027602 | M300005718 |
| 379 | 0.472 | Serpinh1 | serine (or cysteine) peptidase inhibitor, clade H, member 1 Gene | ENSMUSG00000070436 | M400010981 |
| 380 | 0.472 | Cdk2 | cyclin-dependent kinase 2 Gene | ENSMUSG00000025358 | M200000037 |
| 381 | 0.472 | Exosc5 | exosome component 5 Gene | ENSMUSG00000061286 | M200005728 |
| 382 | 0.472 | Igsf1 | immunoglobulin superfamily, member 1 Gene | ENSMUSG00000031111 | M300007562 |
| 383 | 0.472 | Reep5 | receptor accessory protein 5 Gene | ENSMUSG00000005873 | M200004094 |
| 384 | 0.471 | Phf14 | PHD finger protein 14 Gene | ENSMUSG00000029629 | M300006783 |
| 385 | 0.471 | Nf2 | neurofibromatosis 2 Gene | ENSMUSG00000009073 | M300001009 |
| 386 | 0.471 | Prdx1 | peroxiredoxin 1 Gene | ENSMUSG00000028691 | M300006233 |
| 387 | 0.471 | 3110056O03Rik | RIKEN cDNA 3110056O03 gene Gene | ENSMUSG00000035206 | M300009780 |
| 388 | 0.471 | Satb1 | special AT-rich sequence binding protein 1 Gene | ENSMUSG00000023927 | M200001716 |
| 389 | 0.471 | Vps35 | vacuolar protein sorting 35 Gene | ENSMUSG00000031696 | M200011977 |
| 390 | 0.470 | AC034122.1 | Lisch-like isoform 1 | ENSMUSG00000040612 | M400013964 |
| 391 | 0.470 | Ddit4 | DNA-damage-inducible transcript 4 Gene | ENSMUSG00000020108 | M200004194 |
| 392 | 0.470 | Atp5c1 | ATP synthase, H transporting, mitochondrial F1 complex, gamma polypeptide 1 Gene | ENSMUSG00000025781 | M300004763 |
| 393 | 0.470 | 2810022L02Rik | RIKEN cDNA 2810022L02 gene Gene | ENSMUSG00000038305 | M200005657 |
| 394 | 0.470 | Gnas | GNAS (guanine nucleotide binding protein, alpha stimulating) complex locus Gene | ENSMUSG00000027523 | M400001016 |
| 395 | 0.469 | 2410131K14Rik | RIKEN cDNA 2410131K14 gene Gene | ENSMUSG00000032840 | M200008520 |
| 396 | 0.469 | Slc43a3 | solute carrier family 43, member 3 Gene | ENSMUSG00000027074 | M200008829 |
| 397 | 0.469 | Il34 | interleukin 34 Gene | ENSMUSG00000031750 | M300007876 |
| 398 | 0.469 | Klhl5 | kelch-like 5 (Drosophila) Gene | ENSMUSG00000054920 | M300006538 |
| 399 | 0.469 | Pid1 | phosphotyrosine interaction domain containing 1 Gene | ENSMUSG00000045658 | M300016593 |
| 400 | 0.469 | Prkacb | protein kinase, cAMP dependent, catalytic, beta Gene | ENSMUSG00000005034 | M200003462 |
| 401 | 0.468 | Pmf1 | polyamine-modulated factor 1 Gene | ENSMUSG00000028066 | M200007882 |
| 402 | 0.468 | Rhoc | ras homolog gene family, member C Gene | ENSMUSG00000002233 | M200000097 |
| 403 | 0.467 | 4933426K21Rik | RIKEN cDNA 4933426K21 gene Gene | ENSMUSG00000040649 | M400009453 |
| 404 | 0.467 | S100a13 | S100 calcium binding protein A13 Gene | ENSMUSG00000042312 | M200002378 |
| 405 | 0.467 | Med9 | mediator of RNA polymerase II transcription, subunit 9 homolog (yeast) Gene | ENSMUSG00000061650 | M200008968 |
| 406 | 0.467 | Pole3 | polymerase (DNA directed), epsilon 3 (p17 subunit) Gene | ENSMUSG00000028394 | M400011397 |
| 407 | 0.467 | 5930416I19Rik | RIKEN cDNA 5930416I19 gene Gene | ENSMUSG00000048668 | M300019447 |
| 408 | 0.467 | Ly75 | lymphocyte antigen 75 Gene | ENSMUSG00000026980 | M400000977 |
| 409 | 0.467 | Rab31 | RAB31, member RAS oncogene family Gene | ENSMUSG00000056515 | M200006411 |
| 410 | 0.466 | Defb3 | defensin beta 3 Gene | ENSMUSG00000039775 | M300012365 |
| 411 | 0.466 | 1500032L24Rik | RIKEN cDNA 1500032L24 gene Gene | ENSMUSG00000022452 | M200011939 |
| 412 | 0.465 | Eif4g2 | eukaryotic translation initiation factor 4, gamma 2 Gene | ENSMUSG00000005610 | M300000699 |
| 413 | 0.465 | Pdhx | pyruvate dehydrogenase complex, component X Gene | ENSMUSG00000010914 | M300001117 |
| 414 | 0.465 | Psmb7 | proteasome (prosome, macropain) subunit, beta type 7 Gene | ENSMUSG00000026750 | M200000852 |
| 415 | 0.465 | Gtf2f2 | general transcription factor IIF, polypeptide 2 Gene | ENSMUSG00000067995 | M400011582 |
| 416 | 0.465 | Col4a2 | collagen, type IV, alpha 2 Gene | ENSMUSG00000031503 | M200011974 |
| 417 | 0.465 | Wbp1 | WW domain binding protein 1 Gene | ENSMUSG00000030035 | M300006974 |
| 418 | 0.464 | 1810006K21Rik | RIKEN cDNA 1810006K21 gene Gene | ENSMUSG00000036372 | M300010353 |
| 419 | 0.464 | Gusb | glucuronidase, beta Gene | ENSMUSG00000025534 | M300004681 |
| 420 | 0.464 | 2310008M10Rik | RIKEN cDNA 2310008M10 gene Gene | ENSMUSG00000041084 | M400002473 |
| 421 | 0.464 | Tmed3 | transmembrane emp24 domain containing 3 Gene | ENSMUSG00000032353 | M200005621 |
| 422 | 0.464 | Cenpm | centromere protein M Gene | ENSMUSG00000068101 | M400011504 |
| 423 | 0.464 | Insig1 | insulin induced gene 1 Gene | ENSMUSG00000045294 | M300016263 |
| 424 | 0.464 | Smpd3 | sphingomyelin phosphodiesterase 3, neutral Gene | ENSMUSG00000031906 | M200009485 |
| 425 | 0.463 | Aes | amino-terminal enhancer of split Gene | ENSMUSG00000054452 | M300009535 |
| 426 | 0.463 | Mocs2 | molybdenum cofactor synthesis 2 Gene | ENSMUSG00000015536 | M200003720 |
| 427 | 0.462 | Rnaseh2b |  | ENSMUSG00000021932 | M300003020 |
| 428 | 0.462 | Sc4mol | sterol-C4-methyl oxidase-like Gene | ENSMUSG00000031604 | M200006850 |
| 429 | 0.462 | Schip1 | schwannomin interacting protein 1 Gene | ENSMUSG00000027777 | M300005774 |
| 430 | 0.461 | Arrdc4 | arrestin domain containing 4 Gene | ENSMUSG00000042659 | M200013596 |
| 431 | 0.461 | Tmem141 | transmembrane protein 141 Gene | ENSMUSG00000026939 | M300005395 |
| 432 | 0.461 | S100a1 | S100 calcium binding protein A1 Gene | ENSMUSG00000044080 | M200004967 |
| 433 | 0.460 | Peci | peroxisomal delta3, delta2-enoyl-Coenzyme A isomerase Gene | ENSMUSG00000021417 | M200006211 |
| 434 | 0.459 | Pdgfra | platelet derived growth factor receptor, alpha polypeptide Gene | ENSMUSG00000029231 | M200001112 |
| 435 | 0.459 | Hdac3 | histone deacetylase 3 Gene | ENSMUSG00000024454 | M300004138 |
| 436 | 0.458 | Cox7a2l | cytochrome c oxidase subunit VIIa polypeptide 2-like Gene | ENSMUSG00000024248 | M400000731 |
| 437 | 0.458 | E2f8 | E2F transcription factor 8 Gene | ENSMUSG00000046179 | M400003127 |
| 438 | 0.458 | Mmp15 | matrix metallopeptidase 15 Gene | ENSMUSG00000031790 | M300007902 |
| 439 | 0.457 | 2700038C09Rik | RIKEN cDNA 2700038C09 gene Gene | ENSMUSG00000016344 | M200012112 |
| 440 | 0.457 | AC109311.12 | methylenetetrahydrofolate dehydrogenase (NADP+ dependent) 2-like | ENSMUSG00000029376 | M200007209 |
| 441 | 0.457 | Poldip2 | polymerase (DNA-directed), delta interacting protein 2 Gene | ENSMUSG00000001100 | M200012802 |
| 442 | 0.457 | Cbfb | core binding factor beta Gene | ENSMUSG00000031885 | M200000766 |
| 443 | 0.456 | Bzw1 | basic leucine zipper and W2 domains 1 Gene | ENSMUSG00000051223 | M400003801 |
| 444 | 0.456 | H2afv | H2A histone family, member V Gene | ENSMUSG00000041126 | M300013029 |
| 445 | 0.456 | 2700060E02Rik | RIKEN cDNA 2700060E02 gene Gene | ENSMUSG00000021807 | M300002960 |
| 446 | 0.455 | Cxcr4 | chemokine (C-X-C motif) receptor 4 Gene | ENSMUSG00000045382 | M400014726 |
| 447 | 0.455 | RP23-355N5.1 | antagonist of mitotic exit network 1 homolog | ENSMUSG00000068250 | M300021237 |
| 448 | 0.455 | Thy1 | thymus cell antigen 1, theta Gene | ENSMUSG00000032011 | M400001424 |
| 449 | 0.455 | Pts | 6-pyruvoyl-tetrahydropterin synthase Gene | ENSMUSG00000032067 | M300008054 |
| 450 | 0.455 | Srm | spermidine synthase Gene | ENSMUSG00000006442 | M200000004 |
| 451 | 0.455 | Sumo2 | SMT3 suppressor of mif two 3 homolog 2 (yeast) Gene | ENSMUSG00000020738 | M300002370 |
| 452 | 0.454 | Cat | catalase Gene | ENSMUSG00000027187 | M300005501 |
| 453 | 0.454 | Tk1 | thymidine kinase 1 Gene | ENSMUSG00000025574 | M200001015 |
| 454 | 0.454 | Ctsd | cathepsin D Gene | ENSMUSG00000007891 | M400000147 |
| 455 | 0.454 | Vangl2 | vang-like 2 (van gogh, Drosophila) Gene | ENSMUSG00000026556 | M200012875 |
| 456 | 0.454 | 5031439G07Rik | RIKEN cDNA 5031439G07 gene Gene | ENSMUSG00000036046 | M300010219 |
| 457 | 0.454 | AL928608.17 |  | ENSMUSG00000083569 | M400005866 |
| 458 | 0.453 | Glrx | glutaredoxin Gene | ENSMUSG00000021591 | M200006620 |
| 459 | 0.453 | Bub1 | budding uninhibited by benzimidazoles 1 homolog (S. cerevisiae) Gene | ENSMUSG00000027379 | M200000832 |
| 460 | 0.453 | Cox7c | cytochrome c oxidase, subunit VIIc Gene | ENSMUSG00000017778 | M400000275 |
| 461 | 0.453 | Cct5 | chaperonin containing Tcp1, subunit 5 (epsilon) Gene | ENSMUSG00000022234 | M200000704 |
| 462 | 0.452 | Jmjd4 | jumonji domain containing 4 Gene | ENSMUSG00000036819 | M300010546 |
| 463 | 0.452 | 3110003A17Rik | RIKEN cDNA 3110003A17 gene Gene | ENSMUSG00000078453 | M200005858 |
| 464 | 0.452 | Dck | deoxycytidine kinase Gene | ENSMUSG00000029366 | M300006637 |
| 465 | 0.452 | Hnrpab | heterogeneous nuclear ribonucleoprotein A/B Gene | ENSMUSG00000020358 | M200000009 |
| 466 | 0.451 | Ebf3 | early B-cell factor 3 Gene | ENSMUSG00000010476 | M400013942 |
| 467 | 0.451 | P2ry12 | purinergic receptor P2Y, G-protein coupled 12 Gene | ENSMUSG00000036353 | M400011613 |
| 468 | 0.450 | Nedd8 | neural precursor cell expressed, developmentally down-regulated gene 8 Gene | ENSMUSG00000010376 | M300001097 |
| 469 | 0.450 | Optn | optineurin Gene | ENSMUSG00000026672 | M200008850 |
| 470 | 0.450 | Twf1 | twinfilin, actin-binding protein, homolog 1 (Drosophila) Gene | ENSMUSG00000022451 | M300003298 |
| 471 | 0.450 | 6720467C03Rik | RIKEN cDNA 6720467C03 gene Gene | ENSMUSG00000028218 | M200005112 |
| 472 | 0.450 | Lsm10 | U7 snRNP-specific Sm-like protein LSM10 Gene | ENSMUSG00000050188 | M400003658 |
| 473 | 0.450 | Slc35b2 | solute carrier family 35, member B2 Gene | ENSMUSG00000037089 | M200004134 |
| 474 | 0.449 | Gabarap | gamma-aminobutyric acid receptor associated protein Gene | ENSMUSG00000018567 | M200006815 |
| 475 | 0.449 | Uqcrfs1 | ubiquinol-cytochrome c reductase, Rieske iron-sulfur polypeptide 1 Gene | ENSMUSG00000038462 | M200012042 |
| 476 | 0.449 | BC035537 | family with sequence similarity 149, member A Gene | ENSMUSG00000070044 | M400012198 |
| 477 | 0.449 | Psma2 | proteasome (prosome, macropain) subunit, alpha type 2 Gene | ENSMUSG00000015671 | M200012118 |
| 478 | 0.449 | Trappc2l | trafficking protein particle complex 2-like Gene | ENSMUSG00000015013 | M200008106 |
| 479 | 0.448 | Gsn | gelsolin Gene | ENSMUSG00000026879 | M200004057 |
| 480 | 0.448 | A330021E22Rik | RIKEN cDNA A330021E22 gene Gene | ENSMUSG00000040473 | M300012687 |
| 481 | 0.448 | Wdr1 | WD repeat domain 1 Gene | ENSMUSG00000005103 | M200001010 |
| 482 | 0.448 | Polr2d | polymerase (RNA) II (DNA directed) polypeptide D Gene | ENSMUSG00000024258 | M300004017 |
| 483 | 0.448 | Amz2 | archaelysin family metallopeptidase 2 Gene | ENSMUSG00000020610 | M200006537 |
| 484 | 0.447 | Ccnb1 | cyclin B1 Gene | ENSMUSG00000041431 | M200004432 |
| 485 | 0.447 | 2700094K13Rik | RIKEN cDNA 2700094K13 gene Gene | ENSMUSG00000076437 | M400004766 |
| 486 | 0.447 | Freq | frequenin homolog (Drosophila) Gene | ENSMUSG00000062661 | M200013519 |
| 487 | 0.447 | Prmt1 | protein arginine N-methyltransferase 1 Gene | ENSMUSG00000052429 | M200005596 |
| 488 | 0.447 | Aurkb | aurora kinase B Gene | ENSMUSG00000020897 | M300002450 |
| 489 | 0.447 | Psma1 | proteasome (prosome, macropain) subunit, alpha type 1 Gene | ENSMUSG00000030751 | M200006835 |
| 490 | 0.447 | Slc39a8 | solute carrier family 39 (metal ion transporter), member 8 Gene | ENSMUSG00000053897 | M200006914 |
| 491 | 0.446 | Dhcr7 | 7-dehydrocholesterol reductase Gene | ENSMUSG00000058454 | M400001334 |
| 492 | 0.446 | Dyrk2 | dual-specificity tyrosine-(Y)-phosphorylation regulated kinase 2 Gene | ENSMUSG00000028630 | M400001100 |
| 493 | 0.446 | Id1 | inhibitor of DNA binding 1 Gene | ENSMUSG00000042745 | M200000163 |
| 494 | 0.446 | Magoh | mago-nashi homolog, proliferation-associated (Drosophila) Gene | ENSMUSG00000028609 | M200000288 |
| 495 | 0.446 | Ifit2 | interferon-induced protein with tetratricopeptide repeats 2 Gene | ENSMUSG00000045932 | M300016852 |
| 496 | 0.446 | Gpr83 | G protein-coupled receptor 83 Gene | ENSMUSG00000031932 | M200001879 |
| 497 | 0.445 | 1110031B06Rik | RIKEN cDNA 1110031B06 gene Gene | ENSMUSG00000009894 | M400009880 |
| 498 | 0.445 | Crlf2 | cytokine receptor-like factor 2 Gene | ENSMUSG00000033467 | M400013225 |
| 499 | 0.445 | AC123060.4 |  | ENSMUSG00000058700 | M400006451 |
| 500 | 0.445 | Slc25a20 | solute carrier family 25 (mitochondrial carnitine/acylcarnitine translocase), member 20 Gene | ENSMUSG00000032602 | M200006592 |
| 501 | 0.444 | Ak5 | adenylate kinase 5 Gene | ENSMUSG00000039058 | M300011913 |
| 502 | 0.444 | Nol3 | nucleolar protein 3 (apoptosis repressor with CARD domain) Gene | ENSMUSG00000014776 | M200014645 |
| 503 | 0.444 | Rad23b | RAD23b homolog (S. cerevisiae) Gene | ENSMUSG00000028426 | M200012782 |
| 504 | 0.444 | Nasp | nuclear autoantigenic sperm protein (histone-binding) Gene | ENSMUSG00000028693 | M300006234 |
| 505 | 0.444 | Ramp1 | receptor (calcitonin) activity modifying protein 1 Gene | ENSMUSG00000034353 | M200001264 |
| 506 | 0.444 | Taldo1 | transaldolase 1 Gene | ENSMUSG00000025503 | M300004665 |
| 507 | 0.444 | Rhoa | ras homolog gene family, member A Gene | ENSMUSG00000007815 | M300000921 |
| 508 | 0.444 | Slc12a1 | solute carrier family 12, member 1 Gene | ENSMUSG00000027202 | M200001503 |
| 509 | 0.443 | Dtymk | deoxythymidylate kinase Gene | ENSMUSG00000026281 | M400011427 |
| 510 | 0.443 | Drctnnb1a | family with sequence similarity 126, member A Gene | ENSMUSG00000028995 | M300006419 |
| 511 | 0.443 | Dcun1d4 | DCN1, defective in cullin neddylation 1, domain containing 4 (S. cerevisiae) Gene | ENSMUSG00000051674 | M400006137 |
| 512 | 0.442 | Nt5c3l | 5'-nucleotidase, cytosolic III-like Gene | ENSMUSG00000017176 | M300001557 |
| 513 | 0.442 | Pdlim7 | PDZ and LIM domain 7 Gene | ENSMUSG00000021493 | M400008888 |
| 514 | 0.442 | Homer2 | homer homolog 2 (Drosophila) Gene | ENSMUSG00000025813 | M200000086 |
| 515 | 0.441 | AL929446.5 |  | ENSMUSG00000081613 | M400005838 |
| 516 | 0.441 | Apool | apolipoprotein O-like Gene | ENSMUSG00000025525 | M200005424 |
| 517 | 0.441 | Rps9 | ribosomal protein S9 Gene | ENSMUSG00000006333 | M300000782 |
| 518 | 0.441 | Sorbs1 | sorbin and SH3 domain containing 1 Gene | ENSMUSG00000025006 | M400009136 |
| 519 | 0.441 | EG434402 | predicted gene, EG434402 Gene | ENSMUSG00000042293 | M300013622 |
| 520 | 0.440 | Atp5j2 | ATP synthase, H+ transporting, mitochondrial F0 complex, subunit f, isoform 2 Gene | ENSMUSG00000038690 | M400011371 |
| 521 | 0.440 | Ap2s1 | adaptor-related protein complex 2, sigma 1 subunit Gene | ENSMUSG00000008036 | M300000948 |
| 522 | 0.440 | Idh2 | isocitrate dehydrogenase 2 (NADP), mitochondrial Gene | ENSMUSG00000030541 | M300007287 |
| 523 | 0.440 | Psma4 | proteasome (prosome, macropain) subunit, alpha type 4 Gene | ENSMUSG00000032301 | M200006931 |
| 524 | 0.440 | AC162932.2 |  | ENSMUSG00000073240 | M300016267 |
| 525 | 0.440 | AC139042.18 |  | ENSMUSG00000055771 | M400005291 |
| 526 | 0.440 | Commd9 | COMM domain containing 9 Gene | ENSMUSG00000027163 | M300005490 |
| 527 | 0.440 | Taf10 | TAF10 RNA polymerase II, TATA box binding protein (TBP)-associated factor Gene | ENSMUSG00000043866 | M300007488 |
| 528 | 0.439 | 2700050L05Rik | RIKEN cDNA 2700050L05 gene Gene | ENSMUSG00000039990 | M300012474 |
| 529 | 0.439 | Gtf3a | general transcription factor III A Gene | ENSMUSG00000016503 | M300001521 |
| 530 | 0.439 | Hes6 | hairy and enhancer of split 6 (Drosophila) Gene | ENSMUSG00000067071 | M400011312 |
| 531 | 0.439 | Mrpl13 | mitochondrial ribosomal protein L13 Gene | ENSMUSG00000022370 | M200002853 |
| 532 | 0.438 | Kif11 | kinesin family member 11 Gene | ENSMUSG00000012443 | M300001180 |
| 533 | 0.438 | Bat3 | HLA-B-associated transcript 3 Gene | ENSMUSG00000024392 | M300004104 |
| 534 | 0.438 | Atp5d | ATP synthase, H+ transporting, mitochondrial F1 complex, delta subunit Gene | ENSMUSG00000003072 | M300000374 |
| 535 | 0.437 | Ndufb9 | NADH dehydrogenase (ubiquinone) 1 beta subcomplex, 9 Gene | ENSMUSG00000022354 | M300003231 |
| 536 | 0.437 | Pop4 | processing of precursor 4, ribonuclease P/MRP family, (S. cerevisiae) Gene | ENSMUSG00000030423 | M200004345 |
| 537 | 0.436 | Ccdc80 | coiled-coil domain containing 80 Gene | ENSMUSG00000022665 | M300003443 |
| 538 | 0.436 | Ap1s1 | adaptor protein complex AP-1, sigma 1 Gene | ENSMUSG00000004849 | M400010727 |
| 539 | 0.436 | Erc2 | ELKS/RAB6-interacting/CAST family member 2 Gene | ENSMUSG00000040640 | M400012437 |
| 540 | 0.436 | Tmem44 | transmembrane protein 44 Gene | ENSMUSG00000022537 | M300003354 |
| 541 | 0.435 | Slc25a4 | solute carrier family 25 (mitochondrial carrier, adenine nucleotide translocator), member 4 Gene | ENSMUSG00000031633 | M200003422 |
| 542 | 0.435 | Ralb | v-ral simian leukemia viral oncogene homolog B (ras related) Gene | ENSMUSG00000004451 | M300000556 |
| 543 | 0.434 | Ndufa3 | NADH dehydrogenase (ubiquinone) 1 alpha subcomplex, 3 Gene | ENSMUSG00000035674 | M300010040 |
| 544 | 0.433 | Dstn | destrin Gene | ENSMUSG00000015932 | M200006229 |
| 545 | 0.433 | Gng10 | guanine nucleotide binding protein (G protein), gamma 10 Gene | ENSMUSG00000038607 | M200013747 |
| 546 | 0.433 | Mtch1 | mitochondrial carrier homolog 1 (C. elegans) Gene | ENSMUSG00000024012 | M200006584 |
| 547 | 0.432 | Bak1 | BCL2-antagonist/killer 1 Gene | ENSMUSG00000057789 | M300003980 |
| 548 | 0.432 | Commd8 | COMM domain containing 8 Gene | ENSMUSG00000029213 | M200014062 |
| 549 | 0.431 | 1110001J03Rik | formation of mitochondrial complexes 1 homolog (S. cerevisiae) Gene | ENSMUSG00000019689 | M200004783 |
| 550 | 0.431 | AL772308.4 |  | ENSMUSG00000078289 | M400010049 |
| 551 | 0.430 | B830045N13Rik | RIKEN cDNA B830045N13 gene Gene | ENSMUSG00000035131 | M300009718 |
| 552 | 0.430 | 2610029G23Rik | RIKEN cDNA 2610029G23 gene Gene | ENSMUSG00000031226 | M200011952 |
| 553 | 0.430 | Cyb5 | cytochrome b-5 Gene | ENSMUSG00000024646 | M300004251 |
| 554 | 0.429 | BC043098 | family with sequence similarity 168, member B Gene | ENSMUSG00000037503 | M300010963 |
| 555 | 0.429 | Tbcb | tubulin folding cofactor B Gene | ENSMUSG00000006095 | M200005766 |
| 556 | 0.429 | Ndufb8 | NADH dehydrogenase (ubiquinone) 1 beta subcomplex 8 Gene | ENSMUSG00000025204 | M200000786 |
| 557 | 0.429 | Nsdhl |  | ENSMUSG00000031349 | M200008113 |
| 558 | 0.429 | Arf4 | ADP-ribosylation factor 4 Gene | ENSMUSG00000021877 | M200000609 |
| 559 | 0.429 | Slc40a1 | solute carrier family 40 (iron-regulated transporter), member 1 Gene | ENSMUSG00000025993 | M300004878 |
| 560 | 0.428 | AC120548.15 |  | ENSMUSG00000071052 | M400005655 |
| 561 | 0.428 | Brp44l | brain protein 44-like Gene | ENSMUSG00000023861 | M400010089 |
| 562 | 0.428 | Synpr | synaptoporin Gene | ENSMUSG00000056296 | M400005462 |
| 563 | 0.428 | AC121603.4 |  | ENSMUSG00000056849 | M400005656 |
| 564 | 0.428 | Evl | Ena-vasodilator stimulated phosphoprotein Gene | ENSMUSG00000021262 | M300002653 |
| 565 | 0.428 | Limd2 | LIM domain containing 2 Gene | ENSMUSG00000040699 | M200004191 |
| 566 | 0.427 | Selplg | selectin, platelet (p-selectin) ligand Gene | ENSMUSG00000048163 | M200004309 |
| 567 | 0.427 | Wdr25 | WD repeat domain 25 Gene | ENSMUSG00000040877 | M300012914 |
| 568 | 0.427 | Armc10 | armadillo repeat containing 10 Gene | ENSMUSG00000038525 | M200004264 |
| 569 | 0.427 | Plp2 | proteolipid protein 2 Gene | ENSMUSG00000031146 | M300007578 |
| 570 | 0.427 | Zwint | ZW10 interactor Gene | ENSMUSG00000019923 | M300001897 |
| 571 | 0.426 | Eif3s5 | eukaryotic translation initiation factor 3, subunit F Gene | ENSMUSG00000031029 | M200012121 |
| 572 | 0.426 | Cdc42 | cell division cycle 42 homolog (S. cerevisiae) Gene | ENSMUSG00000006699 | M300000845 |
| 573 | 0.425 | Abhd11 | abhydrolase domain containing 11 Gene | ENSMUSG00000040532 | M200005157 |
| 574 | 0.425 | Ube2o | ubiquitin-conjugating enzyme E2O Gene | ENSMUSG00000020802 | M300002396 |
| 575 | 0.425 | Cox18 | COX18 cytochrome c oxidase assembly homolog (S. cerevisiae) Gene | ENSMUSG00000035505 | M400001817 |
| 576 | 0.425 | Nfu1 | NFU1 iron-sulfur cluster scaffold homolog (S. cerevisiae) Gene | ENSMUSG00000029993 | M400011354 |
| 577 | 0.425 | Ttll1 | tubulin tyrosine ligase-like 1 Gene | ENSMUSG00000022442 | M200015371 |
| 578 | 0.424 | Nap1l1 | nucleosome assembly protein 1-like 1 Gene | ENSMUSG00000058799 | M400005271 |
| 579 | 0.424 | Rassf4 | Ras association (RalGDS/AF-6) domain family member 4 Gene | ENSMUSG00000042129 | M300013525 |
| 580 | 0.424 | Tmem9 | transmembrane protein 9 Gene | ENSMUSG00000026411 | M400000923 |
| 581 | 0.423 | 2310007D09Rik | RIKEN cDNA 2310007D09 gene Gene | ENSMUSG00000027654 | M200015690 |
| 582 | 0.423 | Cox7b | cytochrome c oxidase subunit VIIb Gene | ENSMUSG00000031231 | M200009381 |
| 583 | 0.423 | Wdr6 | WD repeat domain 6 Gene | ENSMUSG00000066357 | M200006508 |
| 584 | 0.423 | Nudt19 | nudix (nucleoside diphosphate linked moiety X)-type motif 19 Gene | ENSMUSG00000034875 | M400001743 |
| 585 | 0.423 | Ckap2 | cytoskeleton associated protein 2 Gene | ENSMUSG00000037725 | M400002048 |
| 586 | 0.422 | Dynlrb1 | dynein light chain roadblock-type 1 Gene | ENSMUSG00000047459 | M300018303 |
| 587 | 0.422 | 2900002K06Rik | RIKEN cDNA 2900002K06 gene Gene | ENSMUSG00000055188 | M400005078 |
| 588 | 0.422 | Mobkl2a | MOB1, Mps One Binder kinase activator-like 2A (yeast) Gene | ENSMUSG00000003348 | M300000408 |
| 589 | 0.422 | Srp19 | signal recognition particle 19 Gene | ENSMUSG00000014504 | M300001305 |
| 590 | 0.421 | Fstl1 | follistatin-like 1 Gene | ENSMUSG00000022816 | M300003525 |
| 591 | 0.421 | RP23-168M5.3 | Novel protein | ENSMUSG00000078570 | M300016531 |
| 592 | 0.421 | Cdca7 | cell division cycle associated 7 Gene | ENSMUSG00000055612 | M300001755 |
| 593 | 0.420 | Bri3bp | Bri3 binding protein Gene | ENSMUSG00000037905 | M300011212 |
| 594 | 0.420 | Tagln | transgelin Gene | ENSMUSG00000032085 | M400011145 |
| 595 | 0.420 | Dut | deoxyuridine triphosphatase Gene | ENSMUSG00000027203 | M400012814 |
| 596 | 0.419 | Lyrm2 | LYR motif containing 2 Gene | ENSMUSG00000045854 | M300016780 |
| 597 | 0.418 | Mrpl52 | mitochondrial ribosomal protein L52 Gene | ENSMUSG00000010406 | M200006915 |
| 598 | 0.418 | Traf4 | Tnf receptor associated factor 4 Gene | ENSMUSG00000017386 | M200001791 |
| 599 | 0.417 | Med30 | mediator complex subunit 30 Gene | ENSMUSG00000038622 | M300011673 |
| 600 | 0.417 | Psme1 | proteasome (prosome, macropain) 28 subunit, alpha Gene | ENSMUSG00000022216 | M300018890 |
| 601 | 0.417 | C1d | nuclear DNA binding protein Gene | ENSMUSG00000000581 | M200000938 |
| 602 | 0.416 | Lgmn | legumain Gene | ENSMUSG00000021190 | M200003483 |
| 603 | 0.416 | Cyb5b | cytochrome b5 type B Gene | ENSMUSG00000031924 | M200003839 |
| 604 | 0.416 | Uchl1 | ubiquitin carboxy-terminal hydrolase L1 Gene | ENSMUSG00000029223 | M400011160 |
| 605 | 0.416 | Cyb561d2 | cytochrome b-561 domain containing 2 Gene | ENSMUSG00000037190 | M200005012 |
| 606 | 0.416 | Mia1 | melanoma inhibitory activity 1 Gene | ENSMUSG00000058217 | M400006240 |
| 607 | 0.415 | Socs2 | suppressor of cytokine signaling 2 Gene | ENSMUSG00000020027 | M200001605 |
| 608 | 0.415 | Emid1 | EMI domain containing 1 Gene | ENSMUSG00000034164 | M200000295 |
| 609 | 0.415 | Tuba1c | tubulin, alpha 1C Gene | ENSMUSG00000043091 | M300014179 |
| 610 | 0.414 | Glce | glucuronyl C5-epimerase Gene | ENSMUSG00000032252 | M200009949 |
| 611 | 0.414 | Sh3glb1 | SH3-domain GRB2-like B1 (endophilin) Gene | ENSMUSG00000037062 | M300010714 |
| 612 | 0.413 | Socs3 | suppressor of cytokine signaling 3 Gene | ENSMUSG00000053113 | M200001348 |
| 613 | 0.413 | Cnih3 | cornichon homolog 3 (Drosophila) Gene | ENSMUSG00000026514 | M200015434 |
| 614 | 0.413 | Psmb2 | proteasome (prosome, macropain) subunit, beta type 2 Gene | ENSMUSG00000028837 | M200004330 |
| 615 | 0.412 | Entpd2 | ectonucleoside triphosphate diphosphohydrolase 2 Gene | ENSMUSG00000015085 | M300001354 |
| 616 | 0.412 | Klhl13 | kelch-like 13 (Drosophila) Gene | ENSMUSG00000036782 | M200005293 |
| 617 | 0.412 | Myc | myelocytomatosis oncogene Gene | ENSMUSG00000022346 | M200000925 |
| 618 | 0.412 | Pvrl3 | poliovirus receptor-related 3 Gene | ENSMUSG00000022656 | M300003438 |
| 619 | 0.412 | Wbp5 | WW domain binding protein 5 Gene | ENSMUSG00000042712 | M300013846 |
| 620 | 0.412 | CT009567.9 |  | ENSMUSG00000068487 | M400003089 |
| 621 | 0.411 | Hint2 | histidine triad nucleotide binding protein 2 Gene | ENSMUSG00000028470 | M200005089 |
| 622 | 0.410 | AL845293.5 |  | ENSMUSG00000081999 | M400003423 |
| 623 | 0.410 | Mrpl34 | mitochondrial ribosomal protein L34 Gene | ENSMUSG00000034880 | M200006506 |
| 624 | 0.410 | Slc6a11 | solute carrier family 6 (neurotransmitter transporter, GABA), member 11 Gene | ENSMUSG00000030307 | M300007126 |
| 625 | 0.410 | Vps28 | vacuolar protein sorting 28 (yeast) Gene | ENSMUSG00000062381 | M200006791 |
| 626 | 0.410 | Haao | 3-hydroxyanthranilate 3,4-dioxygenase Gene | ENSMUSG00000000673 | M200006837 |
| 627 | 0.410 | Pomp | proteasome maturation protein Gene | ENSMUSG00000029649 | M200000831 |
| 628 | 0.409 | Bace2 | beta-site APP-cleaving enzyme 2 Gene | ENSMUSG00000040605 | M200005841 |
| 629 | 0.409 | Ndfip2 | Nedd4 family interacting protein 2 Gene | ENSMUSG00000053253 | M400004403 |
| 630 | 0.409 | Stx7 | syntaxin 7 Gene | ENSMUSG00000019998 | M200003010 |
| 631 | 0.408 | 3300001G02Rik | RIKEN cDNA 3300001G02 gene Gene | ENSMUSG00000040767 | M200006744 |
| 632 | 0.408 | Mark4 | MAP/microtubule affinity-regulating kinase 4 Gene | ENSMUSG00000030397 | M300007191 |
| 633 | 0.408 | Tnni1 | troponin I, skeletal, slow 1 Gene | ENSMUSG00000026418 | M300005095 |
| 634 | 0.407 | Zfhx3 | zinc finger homeobox 3 Gene | ENSMUSG00000038872 | M200001664 |
| 635 | 0.407 | 5430432M24Rik | family with sequence similarity 110, member A Gene | ENSMUSG00000027459 | M200012310 |
| 636 | 0.407 | Cdc42se2 | CDC42 small effector 2 Gene | ENSMUSG00000052298 | M400004064 |
| 637 | 0.407 | Rnd3 | Rho family GTPase 3 Gene | ENSMUSG00000017144 | M200014530 |
| 638 | 0.406 | 1810058I24Rik | RIKEN cDNA 1810058I24 gene Gene | ENSMUSG00000073155 | M400013672 |
| 639 | 0.406 | Prickle4 | prickle homolog 4 (Drosophila) Gene | ENSMUSG00000033475 | M400001561 |
| 640 | 0.405 | Akr1a4 | aldo-keto reductase family 1, member A4 (aldehyde reductase) Gene | ENSMUSG00000028692 | M200006829 |
| 641 | 0.405 | Shisa4 | shisa homolog 4 (Xenopus laevis) Gene | ENSMUSG00000041889 | M300013417 |
| 642 | 0.405 | Oxct1 | 3-oxoacid CoA transferase 1 Gene | ENSMUSG00000022186 | M400000547 |
| 643 | 0.404 | Gpx7 | glutathione peroxidase 7 Gene | ENSMUSG00000028597 | M200003823 |
| 644 | 0.404 | Khdrbs3 | KH domain containing, RNA binding, signal transduction associated 3 Gene | ENSMUSG00000022332 | M200003563 |
| 645 | 0.403 | Phlda3 | pleckstrin homology-like domain, family A, member 3 Gene | ENSMUSG00000041801 | M400002551 |
| 646 | 0.403 | 2010204K13Rik | RIKEN cDNA 2010204K13 gene Gene | ENSMUSG00000063018 | M300021184 |
| 647 | 0.403 | Mpzl1 | myelin protein zero-like 1 Gene | ENSMUSG00000026566 | M300005180 |
| 648 | 0.403 | Oprl1 | opioid receptor-like 1 Gene | ENSMUSG00000027584 | M400011092 |
| 649 | 0.402 | 5730446C15Rik | family with sequence similarity 108, member B Gene | ENSMUSG00000047368 | M400011926 |
| 650 | 0.402 | Tes | testis derived transcript Gene | ENSMUSG00000029552 | M400001181 |
| 651 | 0.402 | Fxc1 | fractured callus expressed transcript 1 Gene | ENSMUSG00000030882 | M200001569 |
| 652 | 0.402 | Pdcd5 | programmed cell death 5 Gene | ENSMUSG00000030417 | M200004183 |
| 653 | 0.401 | Mcm3 | minichromosome maintenance deficient 3 (S. cerevisiae) Gene | ENSMUSG00000041859 | M300013395 |
| 654 | 0.401 | Kif21b | kinesin family member 21B Gene | ENSMUSG00000041642 | M300013278 |
| 655 | 0.401 | Lmcd1 | LIM and cysteine-rich domains 1 Gene | ENSMUSG00000057604 | M200012898 |
| 656 | 0.399 | Bcl11b | B-cell leukemia/lymphoma 11B Gene | ENSMUSG00000048251 | M400014858 |
| 657 | 0.399 | Stab1 | stabilin 1 Gene | ENSMUSG00000042286 | M400002608 |
| 658 | 0.399 | Ndufs6 | NADH dehydrogenase (ubiquinone) Fe-S protein 6 Gene | ENSMUSG00000021606 | M400000492 |
| 659 | 0.398 | AC158396.2-201 |  | ENSMUSG00000072714 | M400009999 |
| 660 | 0.397 | 4930452B06Rik | RIKEN cDNA 4930452B06 gene Gene | ENSMUSG00000021747 | M200012674 |
| 661 | 0.397 | Pde1a | phosphodiesterase 1A, calmodulin-dependent Gene | ENSMUSG00000059173 | M200008305 |
| 662 | 0.397 | Gnl3l | guanine nucleotide binding protein-like 3 (nucleolar)-like Gene | ENSMUSG00000025266 | M300004547 |
| 663 | 0.397 | Mpdu1 | mannose-P-dolichol utilization defect 1 Gene | ENSMUSG00000018761 | M200016004 |
| 664 | 0.397 | Ptk2b | PTK2 protein tyrosine kinase 2 beta Gene | ENSMUSG00000059456 | M300003077 |
| 665 | 0.396 | Acat1 | acetyl-Coenzyme A acetyltransferase 1 Gene | ENSMUSG00000032047 | M300008045 |
| 666 | 0.396 | Dgat2 | diacylglycerol O-acyltransferase 2 Gene | ENSMUSG00000030747 | M200011933 |
| 667 | 0.396 | Cox7a2 | cytochrome c oxidase, subunit VIIa 2 Gene | ENSMUSG00000032330 | M200000818 |
| 668 | 0.395 | Ahi1 | Abelson helper integration site 1 Gene | ENSMUSG00000019986 | M300001939 |
| 669 | 0.394 | Ndufb2 | NADH dehydrogenase (ubiquinone) 1 beta subcomplex, 2 Gene | ENSMUSG00000002416 | M200006474 |
| 670 | 0.394 | 1810037I17Rik | RIKEN cDNA 1810037I17 gene Gene | ENSMUSG00000054091 | M400004704 |
| 671 | 0.393 | Banf1 | barrier to autointegration factor 1 Gene | ENSMUSG00000024844 | M200002560 |
| 672 | 0.393 | Fxyd6 | FXYD domain-containing ion transport regulator 6 Gene | ENSMUSG00000066705 | M400011405 |
| 673 | 0.392 | Abr | active BCR-related gene Gene | ENSMUSG00000017631 | M300001591 |
| 674 | 0.392 | Cops5 | COP9 (constitutive photomorphogenic) homolog, subunit 5 (Arabidopsis thaliana) Gene | ENSMUSG00000025917 | M200000933 |
| 675 | 0.392 | H19 | H19 fetal liver mRNA Gene | ENSMUSG00000000031 | M300000002 |
| 676 | 0.391 | Eif3k | eukaryotic translation initiation factor 3, subunit K Gene | ENSMUSG00000053565 | M300007308 |
| 677 | 0.391 | Ngfrap1 | nerve growth factor receptor (TNFRSF16) associated protein 1 Gene | ENSMUSG00000046432 | M200016095 |
| 678 | 0.391 | AC116736.14 | Putative uncharacterized protein | ENSMUSG00000051761 | M400003880 |
| 679 | 0.391 | Hoxa5 | homeo box A5 Gene | ENSMUSG00000038253 | M200000062 |
| 680 | 0.391 | Tbc1d7 | TBC1 domain family, member 7 Gene | ENSMUSG00000021368 | M200013638 |
| 681 | 0.390 | Ihpk2 | inositol hexaphosphate kinase 2 Gene | ENSMUSG00000032599 | M200016005 |
| 682 | 0.390 | Rap1a | RAS-related protein-1a Gene | ENSMUSG00000068798 | M400011902 |
| 683 | 0.390 | Ndufv2 | NADH dehydrogenase (ubiquinone) flavoprotein 2 Gene | ENSMUSG00000024099 | M200000835 |
| 684 | 0.390 | Pbx4 | pre-B-cell leukemia homeobox 4 Gene | ENSMUSG00000031860 | M300007937 |
| 685 | 0.389 | Paqr7 | progestin and adipoQ receptor family member VII Gene | ENSMUSG00000037348 | M200009388 |
| 686 | 0.389 | 6330419J24Rik | RIKEN cDNA 6330419J24 gene Gene | ENSMUSG00000054850 | M400004958 |
| 687 | 0.389 | Ppic | peptidylprolyl isomerase C Gene | ENSMUSG00000024538 | M200001825 |
| 688 | 0.389 | Casp7 | caspase 7 Gene | ENSMUSG00000025076 | M300004466 |
| 689 | 0.388 | Ankrd6 | ankyrin repeat domain 6 Gene | ENSMUSG00000040183 | M300012553 |
| 690 | 0.387 | Ddt | D-dopachrome tautomerase Gene | ENSMUSG00000001666 | M200002268 |
| 691 | 0.387 | Eif3i | eukaryotic translation initiation factor 3, subunit I Gene | ENSMUSG00000028798 | M200008679 |
| 692 | 0.386 | Ccdc34 | coiled-coil domain containing 34 Gene | ENSMUSG00000027160 | M300005489 |
| 693 | 0.386 | Mrpl43 | mitochondrial ribosomal protein L43 Gene | ENSMUSG00000025208 | M300004513 |
| 694 | 0.386 | Ppp2r2b | protein phosphatase 2 (formerly 2A), regulatory subunit B (PR 52), beta isoform Gene | ENSMUSG00000024500 | M300004178 |
| 695 | 0.385 | Apex1 | apurinic/apyrimidinic endonuclease 1 Gene | ENSMUSG00000035960 | M300010175 |
| 696 | 0.384 | C530008M17Rik | RIKEN cDNA C530008M17 gene Gene | ENSMUSG00000036377 | M300010354 |
| 697 | 0.384 | Epb4.1l2 | erythrocyte protein band 4.1-like 2 Gene | ENSMUSG00000019978 | M400008704 |
| 698 | 0.384 | Tead2 | TEA domain family member 2 Gene | ENSMUSG00000030796 | M200001157 |
| 699 | 0.383 | A930005H10Rik | RIKEN cDNA A930005H10 gene Gene | ENSMUSG00000054426 | M400004807 |
| 700 | 0.383 | Ick | intestinal cell kinase Gene | ENSMUSG00000009828 | M200014116 |
| 701 | 0.383 | Zfp57 | zinc finger protein 57 Gene | ENSMUSG00000036036 | M200004111 |
| 702 | 0.382 | AC109220.16 |  | ENSMUSG00000083820 | M400002187 |
| 703 | 0.382 | Esco2 | establishment of cohesion 1 homolog 2 (S. cerevisiae) Gene | ENSMUSG00000022034 | M200014513 |
| 704 | 0.382 | Fau | Finkel-Biskis-Reilly murine sarcoma virus (FBR-MuSV) ubiquitously expressed (fox derived) Gene | ENSMUSG00000038274 | M400002114 |
| 705 | 0.382 | Mmab | methylmalonic aciduria (cobalamin deficiency) type B homolog (human) Gene | ENSMUSG00000029575 | M200012831 |
| 706 | 0.382 | Rrm2 | ribonucleotide reductase M2 Gene | ENSMUSG00000020649 | M200000030 |
| 707 | 0.382 | Rerg | RAS-like, estrogen-regulated, growth-inhibitor Gene | ENSMUSG00000030222 | M400001238 |
| 708 | 0.381 | Pgrmc1 | progesterone receptor membrane component 1 Gene | ENSMUSG00000006373 | M300000793 |
| 709 | 0.381 | Snx7 | sorting nexin 7 Gene | ENSMUSG00000028007 | M200007424 |
| 710 | 0.381 | 2610110G12Rik | RIKEN cDNA 2610110G12 gene Gene | ENSMUSG00000024426 | M300004122 |
| 711 | 0.380 | Sf3b5 | splicing factor 3b, subunit 5 Gene | ENSMUSG00000078348 | M200006630 |
| 712 | 0.379 | Cox5a | cytochrome c oxidase, subunit Va Gene | ENSMUSG00000000088 | M300000005 |
| 713 | 0.378 | Bdh1 | 3-hydroxybutyrate dehydrogenase, type 1 Gene | ENSMUSG00000046598 | M200006708 |
| 714 | 0.378 | Pvrl2 | poliovirus receptor-related 2 Gene | ENSMUSG00000062300 | M400000063 |
| 715 | 0.378 | Impdh1 | inosine 5'-phosphate dehydrogenase 1 Gene | ENSMUSG00000003500 | M400000073 |
| 716 | 0.378 | Olfm2 | olfactomedin 2 Gene | ENSMUSG00000032172 | M300008097 |
| 717 | 0.377 | Dcakd | dephospho-CoA kinase domain containing Gene | ENSMUSG00000020935 | M200012012 |
| 718 | 0.377 | Fxyd7 | FXYD domain-containing ion transport regulator 7 Gene | ENSMUSG00000036578 | M200013741 |
| 719 | 0.376 | AC190403.1 |  | ENSMUSG00000035129 | M400001756 |
| 720 | 0.376 | Loxl1 | lysyl oxidase-like 1 Gene | ENSMUSG00000032334 | M300008195 |
| 721 | 0.375 | Dda1 | DET1 and DDB1 associated 1 Gene | ENSMUSG00000074247 | M200006719 |
| 722 | 0.375 | Cdk4 | cyclin-dependent kinase 4 Gene | ENSMUSG00000006728 | M200002442 |
| 723 | 0.375 | Pak3 | p21 (CDKN1A)-activated kinase 3 Gene | ENSMUSG00000031284 | M200001308 |
| 724 | 0.374 | 2010107E04Rik | RIKEN cDNA 2010107E04 gene Gene | ENSMUSG00000021290 | M200000112 |
| 725 | 0.374 | Actr1a | ARP1 actin-related protein 1 homolog A, centractin alpha (yeast) Gene | ENSMUSG00000025228 | M300004523 |
| 726 | 0.373 | Ppib | peptidylprolyl isomerase B Gene | ENSMUSG00000032383 | M200000909 |
| 727 | 0.373 | Manbal | mannosidase, beta A, lysosomal-like Gene | ENSMUSG00000063019 | M200016286 |
| 728 | 0.373 | Ndufa11 | NADH dehydrogenase (ubiquinone) 1 alpha subcomplex 11 Gene | ENSMUSG00000002379 | M200005413 |
| 729 | 0.373 | Rap2a | RAS related protein 2a Gene | ENSMUSG00000051615 | M300022233 |
| 730 | 0.372 | Ncapg | on-SMC condensin I complex, subunit G Gene | ENSMUSG00000015880 | M400008584 |
| 731 | 0.372 | Slc16a1 | solute carrier family 16 (monocarboxylic acid transporters), member 1 Gene | ENSMUSG00000032902 | M200002767 |
| 732 | 0.371 | Cct6a | chaperonin containing Tcp1, subunit 6a (zeta) Gene | ENSMUSG00000029447 | M400001166 |
| 733 | 0.371 | Hoxb5 | homeo box B5 Gene | ENSMUSG00000038700 | M200000076 |
| 734 | 0.371 | Rgs19 | regulator of G-protein signaling 19 Gene | ENSMUSG00000002458 | M200003820 |
| 735 | 0.371 | Txndc17 | thioredoxin domain containing 17 Gene | ENSMUSG00000020803 | M200005648 |
| 736 | 0.369 | Hras1 | Harvey rat sarcoma virus oncogene 1 Gene | ENSMUSG00000025499 | M400010813 |
| 737 | 0.369 | Sin3b | transcriptional regulator, SIN3B (yeast) Gene | ENSMUSG00000031622 | M300007824 |
| 738 | 0.369 | Ube2c | ubiquitin-conjugating enzyme E2C Gene | ENSMUSG00000001403 | M300000188 |
| 739 | 0.368 | R74862 |  | ENSMUSG00000059277 | M200002223 |
| 740 | 0.367 | CT010576.9 |  | ENSMUSG00000052192 | M400004034 |
| 741 | 0.367 | Psmb1 | proteasome (prosome, macropain) subunit, beta type 1 Gene | ENSMUSG00000014769 | M300001326 |
| 742 | 0.367 | Rsu1 | Ras suppressor protein 1 Gene | ENSMUSG00000026727 | M200000322 |
| 743 | 0.367 | Eif3h | eukaryotic translation initiation factor 3, subunit H Gene | ENSMUSG00000022312 | M400010040 |
| 744 | 0.366 | Acat2 | acetyl-Coenzyme A acetyltransferase 2 Gene | ENSMUSG00000023832 | M200012683 |
| 745 | 0.365 | Psmd8 | proteasome (prosome, macropain) 26S subunit, non-ATPase, 8 Gene | ENSMUSG00000030591 | M200007825 |
| 746 | 0.364 | Limk1 | LIM-domain containing, protein kinase Gene | ENSMUSG00000029674 | M200003373 |
| 747 | 0.363 | AC154266.2 |  | ENSMUSG00000069196 | M400011265 |
| 748 | 0.363 | Agpat1 | 1-acylglycerol-3-phosphate O-acyltransferase 1 (lysophosphatidic acid acyltransferase, alpha) Gene | ENSMUSG00000034254 | M200002732 |
| 749 | 0.363 | Vat1 | vesicle amine transport protein 1 homolog (T californica) Gene | ENSMUSG00000034993 | M300009648 |
| 750 | 0.362 | Sh3bgrl | SH3-binding domain glutamic acid-rich protein like Gene | ENSMUSG00000031246 | M200004130 |
| 751 | 0.362 | Sepp1 | selenoprotein P, plasma, 1 Gene | ENSMUSG00000064373 | M400010899 |
| 752 | 0.361 | Acot1 | acyl-CoA thioesterase 1 Gene | ENSMUSG00000072949 | M300013759 |
| 753 | 0.361 | Kif4 | kinesin family member 4 Gene | ENSMUSG00000034311 | M300009278 |
| 754 | 0.360 | Cox6b1 | cytochrome c oxidase, subunit VIb polypeptide 1 Gene | ENSMUSG00000036751 | M200000150 |
| 755 | 0.360 | Lhfpl3 | lipoma HMGIC fusion partner-like 3 Gene | ENSMUSG00000058361 | M400006300 |
| 756 | 0.360 | Samd4b | sterile alpha motif domain containing 4B Gene | ENSMUSG00000037513 | M300010971 |
| 757 | 0.360 | Mad2l2 | MAD2 mitotic arrest deficient-like 2 (yeast) Gene | ENSMUSG00000029003 | M200002809 |
| 758 | 0.360 | Pde1b | phosphodiesterase 1B, Ca2+calmodulin dependent Gene | ENSMUSG00000022489 | M200000021 |
| 759 | 0.359 | Sqle | squalene epoxidase Gene | ENSMUSG00000022351 | M200004466 |
| 760 | 0.358 | Pfn | perforin 1 (pore forming protein) Gene | ENSMUSG00000027805 | M400001029 |
| 761 | 0.358 | Eif4a1 | eukaryotic translation initiation factor 4A1 Gene | ENSMUSG00000059796 | M400000306 |
| 762 | 0.357 | Gipc1 | GIPC PDZ domain containing family, member 1 Gene | ENSMUSG00000019433 | M200004021 |
| 763 | 0.357 | Nrarp | Notch-regulated ankyrin repeat protein Gene | ENSMUSG00000078202 | M400011526 |
| 764 | 0.357 | Foxo6 | forkhead box O6 Gene | ENSMUSG00000052135 | M400004007 |
| 765 | 0.356 | Hcrtr1 | hypocretin (orexin) receptor 1 Gene | ENSMUSG00000028778 | M300006286 |
| 766 | 0.356 | Mfsd2 | major facilitator superfamily domain containing 2 Gene | ENSMUSG00000028655 | M200008045 |
| 767 | 0.355 | Ranbp1 | RAN binding protein 1 Gene | ENSMUSG00000005732 | M200001437 |
| 768 | 0.354 | Sox11 | SRY-box containing gene 11 Gene | ENSMUSG00000063632 | M400013413 |
| 769 | 0.354 | Scn3a | sodium channel, voltage-gated, type III, alpha Gene | ENSMUSG00000057182 | M400000982 |
| 770 | 0.353 | Nav1 | neuron navigator 1 Gene | ENSMUSG00000009418 | M300001042 |
| 771 | 0.353 | Mllt11 | myeloid/lymphoid or mixed-lineage leukemia (trithorax homolog, Drosophila); translocated to, 11 Gene | ENSMUSG00000053192 | M200005065 |
| 772 | 0.352 | Hmmr | hyaluronan mediated motility receptor (RHAMM) Gene | ENSMUSG00000020330 | M400000383 |
| 773 | 0.352 | Skp1a | S-phase kinase-associated protein 1A Gene | ENSMUSG00000036309 | M300010327 |
| 774 | 0.351 | Shb | src homology 2 domain-containing transforming protein B Gene | ENSMUSG00000044813 | M300015805 |
| 775 | 0.351 | Tmem158 | transmembrane protein 158 Gene | ENSMUSG00000054871 | M400004961 |
| 776 | 0.351 | Tpx2 | deleted in azoospermia-like Gene | ENSMUSG00000027469 | M200003024 |
| 777 | 0.350 | D4Bwg0951e | Uncharacterized protein C9orf150 homolog | ENSMUSG00000048706 | M200007845 |
| 778 | 0.350 | Ndufs8 | NADH dehydrogenase (ubiquinone) Fe-S protein 8 Gene | ENSMUSG00000059734 | M300004360 |
| 779 | 0.348 | Kpna2 | karyopherin (importin) alpha 2 Gene | ENSMUSG00000018362 | M400000288 |
| 780 | 0.347 | C230078M08Rik | RIKEN cDNA C230078M08 gene Gene | ENSMUSG00000047439 | M300018282 |
| 781 | 0.347 | Slc25a1 | solute carrier family 25 (mitochondrial carrier, citrate transporter), member 1 Gene | ENSMUSG00000003528 | M200004472 |
| 782 | 0.347 | Igfbp4 | insulin-like growth factor binding protein 4 Gene | ENSMUSG00000017493 | M200004334 |
| 783 | 0.347 | Cx3cl1 | chemokine (C-X3-C motif) ligand 1 Gene | ENSMUSG00000031778 | M200001237 |
| 784 | 0.347 | Ier5l | immediate early response 5-like Gene | ENSMUSG00000078200 | M400011699 |
| 785 | 0.347 | Csrp2 | cysteine and glycine-rich protein 2 Gene | ENSMUSG00000020186 | M400010759 |
| 786 | 0.346 | Arpc3 | actin related protein 2/3 complex, subunit 3 Gene | ENSMUSG00000029465 | M200004909 |
| 787 | 0.346 | Gnb1 | guanine nucleotide binding protein (G protein), beta 1 Gene | ENSMUSG00000029064 | M200000883 |
| 788 | 0.346 | Hmgcr | 3-hydroxy-3-methylglutaryl-Coenzyme A reductase Gene | ENSMUSG00000021670 | M400008904 |
| 789 | 0.344 | Rps6ka6 | ribosomal protein S6 kinase polypeptide 6 Gene | ENSMUSG00000025665 | M300004731 |
| 790 | 0.344 | Atg9b | ATG9 autophagy related 9 homolog B (S. cerevisiae) Gene | ENSMUSG00000038295 | M300011479 |
| 791 | 0.344 | Ndufc1 | NADH dehydrogenase (ubiquinone) 1, subcomplex unknown, 1 Gene | ENSMUSG00000037152 | M400012805 |
| 792 | 0.343 | Serf1 | small EDRK-rich factor 1 Gene | ENSMUSG00000021643 | M200000687 |
| 793 | 0.343 | 5730559C18Rik | RIKEN cDNA 5730559C18 gene Gene | ENSMUSG00000041605 | M300005088 |
| 794 | 0.343 | Lpl | lipoprotein lipase Gene | ENSMUSG00000015568 | M300001423 |
| 795 | 0.343 | Unc119 | unc-119 homolog (C. elegans) Gene | ENSMUSG00000002058 | M200005682 |
| 796 | 0.343 | Lars2 | leucyl-tRNA synthetase, mitochondrial Gene | ENSMUSG00000035202 | M300009774 |
| 797 | 0.341 | Ccdc23 | coiled-coil domain containing 23 Gene | ENSMUSG00000028643 | M200007879 |
| 798 | 0.340 | Lmo4 | LIM domain only 4 Gene | ENSMUSG00000028266 | M200006369 |
| 799 | 0.340 | Pdzrn3 | PDZ domain containing RING finger 3 Gene | ENSMUSG00000035357 | M300009878 |
| 800 | 0.340 | Csnk1e | casein kinase 1, epsilon Gene | ENSMUSG00000022433 | M200016278 |
| 801 | 0.339 | Cyp51 | cytochrome P450, family 51 Gene | ENSMUSG00000001467 | M200009287 |
| 802 | 0.338 | Dera | 2-deoxyribose-5-phosphate aldolase homolog (C. elegans) Gene | ENSMUSG00000030225 | M400001239 |
| 803 | 0.338 | Dlk2 | delta-like 2 homolog (Drosophila) Gene | ENSMUSG00000047428 | M400003306 |
| 804 | 0.338 | AC167978.4 |  | ENSMUSG00000066620 | M400008233 |
| 805 | 0.338 | Cox6a1 | cytochrome c oxidase, subunit VI a, polypeptide 1 Gene | ENSMUSG00000041697 | M200013993 |
| 806 | 0.338 | E2f1 | E2F transcription factor 1 Gene | ENSMUSG00000027490 | M300005663 |
| 807 | 0.334 | Slc18a2 | solute carrier family 18 (vesicular monoamine), member 2 Gene | ENSMUSG00000025094 | M300004482 |
| 808 | 0.334 | Dynlt1 | dynein light chain Tctex-type 1D Gene | ENSMUSG00000000579 | M300000090 |
| 809 | 0.332 | Ccdc95 | INO80 complex subunit E Gene | ENSMUSG00000030689 | M300019970 |
| 810 | 0.332 | Slc25a43 | solute carrier family 25, member 43 Gene | ENSMUSG00000037636 | M300011038 |
| 811 | 0.331 | Dos | downstream of Stk11 Gene | ENSMUSG00000035640 | M300010025 |
| 812 | 0.330 | Eef1e1 | eukaryotic translation elongation factor 1 epsilon 1 Gene | ENSMUSG00000001707 | M200007877 |
| 813 | 0.329 | Lypla2 | lysophospholipase 2 Gene | ENSMUSG00000028670 | M200007536 |
| 814 | 0.329 | Bmp4 | bone morphogenetic protein 4 Gene | ENSMUSG00000021835 | M200002432 |
| 815 | 0.328 | 1110008P14Rik | RIKEN cDNA 1110008P14 gene Gene | ENSMUSG00000039195 | M300012020 |
| 816 | 0.328 | Ppp2r2a | protein phosphatase 2 (formerly 2A), regulatory subunit B (PR 52), alpha isoform Gene | ENSMUSG00000022052 | M400000535 |
| 817 | 0.327 | Psmb3 | proteasome (prosome, macropain) subunit, beta type 3 Gene | ENSMUSG00000069744 | M400005941 |
| 818 | 0.326 | 0610009D07Rik | RIKEN cDNA 0610009D07 gene Gene | ENSMUSG00000037361 | M200006613 |
| 819 | 0.325 | Nefl | neurofilament, light polypeptide Gene | ENSMUSG00000022055 | M200000742 |
| 820 | 0.325 | Atpif1 | ATPase inhibitory factor 1 Gene | ENSMUSG00000054428 | M400004809 |
| 821 | 0.324 | Odc1 | ornithine decarboxylase, structural 1 Gene | ENSMUSG00000011179 | M300001129 |
| 822 | 0.324 | Phlda1 | pleckstrin homology-like domain, family A, member 1 Gene | ENSMUSG00000020205 | M200001196 |
| 823 | 0.323 | AC151732.2 |  | ENSMUSG00000069962 | M400005977 |
| 824 | 0.323 | Pafah1b3 | platelet-activating factor acetylhydrolase, isoform 1b, alpha1 subunit Gene | ENSMUSG00000005447 | M200000211 |
| 825 | 0.321 | AC123752.8 |  | ENSMUSG00000078291 | M400006353 |
| 826 | 0.320 | Cxadr | coxsackie virus and adenovirus receptor Gene | ENSMUSG00000022865 | M400013381 |
| 827 | 0.319 | Cgref1 | cell growth regulator with EF hand domain 1 Gene | ENSMUSG00000029161 | M200014182 |
| 828 | 0.318 | Ndufa4 | NADH dehydrogenase (ubiquinone) 1 alpha subcomplex, 4 Gene | ENSMUSG00000029632 | M200013792 |
| 829 | 0.318 | Hist2h2ac | histone cluster 2, H2ac Gene | ENSMUSG00000068855 | M400012323 |
| 830 | 0.316 | Gpr17 | G protein-coupled receptor 17 Gene | ENSMUSG00000052229 | M300020359 |
| 831 | 0.316 | Nkiras2 | NFKB inhibitor interacting Ras-like protein 2 Gene | ENSMUSG00000017837 | M300001613 |
| 832 | 0.316 | Sh3bgrl3 | SH3 domain binding glutamic acid-rich protein-like 3 Gene | ENSMUSG00000028843 | M200004332 |
| 833 | 0.315 | 2010317E24Rik | RIKEN cDNA 2010317E24 gene Gene | ENSMUSG00000026955 | M400000976 |
| 834 | 0.315 | Ccna2 | cyclin A2 Gene | ENSMUSG00000027715 | M300005764 |
| 835 | 0.314 | Numbl | numb-like Gene | ENSMUSG00000063160 | M400011088 |
| 836 | 0.314 | Dok4 | docking protein 4 Gene | ENSMUSG00000040631 | M200013028 |
| 837 | 0.313 | Pcdhgc4 | protocadherin gamma subfamily A, 11 Gene | ENSMUSG00000023036 | M400009080 |
| 838 | 0.312 | Lrrtm1 | leucine rich repeat transmembrane neuronal 1 Gene | ENSMUSG00000060780 | M200004818 |
| 839 | 0.312 | AC175032.1-203 | Transmembrane protein C10orf57 homolog | ENSMUSG00000072676 | M200016354 |
| 840 | 0.312 | Alpk1 | alpha-kinase 1 Gene | ENSMUSG00000028028 | M400005807 |
| 841 | 0.312 | Isoc1 | isochorismatase domain containing 1 Gene | ENSMUSG00000024601 | M400000763 |
| 842 | 0.312 | Ngef | neuronal guanine nucleotide exchange factor Gene | ENSMUSG00000026259 | M200009440 |
| 843 | 0.311 | Mcm6 | minichromosome maintenance deficient 6 (MIS5 homolog, S. pombe) (S. cerevisiae) Gene | ENSMUSG00000026355 | M200002043 |
| 844 | 0.311 | Atox1 | ATX1 (antioxidant protein 1) homolog 1 (yeast) Gene | ENSMUSG00000018585 | M300001695 |
| 845 | 0.310 | Uhrf1 | ubiquitin-like, containing PHD and RING finger domains, 1 Gene | ENSMUSG00000001228 | M400011084 |
| 846 | 0.309 | Gnb2 | guanine nucleotide binding protein (G protein), beta 2 Gene | ENSMUSG00000029713 | M200006860 |
| 847 | 0.309 | Mgst3 | microsomal glutathione S-transferase 3 Gene | ENSMUSG00000026688 | M300005240 |
| 848 | 0.309 | Mycn | v-myc myelocytomatosis viral related oncogene, neuroblastoma derived (avian) Gene | ENSMUSG00000037169 | M200003436 |
| 849 | 0.308 | Smarcd3 | SWI/SNF related, matrix associated, actin dependent regulator of chromatin, subfamily d, member 3 Gene | ENSMUSG00000028949 | M200009308 |
| 850 | 0.308 | F2r | coagulation factor II (thrombin) receptor Gene | ENSMUSG00000048376 | M300019182 |
| 851 | 0.307 | Cdc20 | cell division cycle 20 homolog (S. cerevisiae) Gene | ENSMUSG00000006398 | M300000799 |
| 852 | 0.307 | Sirt2 | sirtuin 2 (silent mating type information regulation 2, homolog) 2 (S. cerevisiae) Gene | ENSMUSG00000015149 | M200005063 |
| 853 | 0.306 | Nipsnap3a | nipsnap homolog 3A (C. elegans) Gene | ENSMUSG00000015247 | M200008035 |
| 854 | 0.305 | Casp3 | caspase 3 Gene | ENSMUSG00000031628 | M400010978 |
| 855 | 0.305 | Cpne6 | copine VI Gene | ENSMUSG00000022212 | M200002222 |
| 856 | 0.303 | Ppp1r14b | protein phosphatase 1, regulatory (inhibitor) subunit 14B Gene | ENSMUSG00000056612 | M400005565 |
| 857 | 0.302 | CT010471.11 |  | ENSMUSG00000063180 | M400008351 |
| 858 | 0.302 | Meis2 | Meis homeobox 2 Gene | ENSMUSG00000027210 | M400000987 |
| 859 | 0.299 | Fabp7 | fatty acid binding protein 7, brain Gene | ENSMUSG00000019874 | M400000340 |
| 860 | 0.299 | Rasgef1b | RasGEF domain family, member 1B Gene | ENSMUSG00000029333 | M300006616 |
| 861 | 0.298 | Lhx2 | LIM homeobox protein 2 Gene | ENSMUSG00000000247 | M200009425 |
| 862 | 0.298 | Serpina3n | serine (or cysteine) peptidase inhibitor, clade A, member 3N Gene | ENSMUSG00000021091 | M300002557 |
| 863 | 0.296 | Scn3b | sodium channel, voltage-gated, type III, beta Gene | ENSMUSG00000049281 | M400012915 |
| 864 | 0.296 | Sfrp1 | secreted frizzled-related protein 1 Gene | ENSMUSG00000031548 | M400013755 |
| 865 | 0.293 | Gata3 | GATA binding protein 3 Gene | ENSMUSG00000015619 | M200000214 |
| 866 | 0.293 | Pgls | 6-phosphogluconolactonase Gene | ENSMUSG00000031807 | M200006491 |
| 867 | 0.292 | Mcts2 | malignant T cell amplified sequence 2 Gene | ENSMUSG00000042814 | M400011498 |
| 868 | 0.292 | Cdca3 | cell division cycle associated 3 Gene | ENSMUSG00000023505 | M200004327 |
| 869 | 0.292 | Tmem176b | transmembrane protein 176B Gene | ENSMUSG00000029810 | M200005978 |
| 870 | 0.288 | AC115045.13 | Transmembrane protein 176A (Kidney-expressed gene 2 protein)(Gene signature 188) | ENSMUSG00000023367 | M200005402 |
| 871 | 0.288 | Igsf3 | immunoglobulin superfamily, member 3 Gene | ENSMUSG00000042035 | M400002573 |
| 872 | 0.288 | Tmem179 | transmembrane protein 179 Gene | ENSMUSG00000054013 | M400004673 |
| 873 | 0.287 | Snx26 | sorting nexin 26 Gene | ENSMUSG00000036882 | M300010599 |
| 874 | 0.287 | Mki67 | antigen identified by monoclonal antibody Ki 67 Gene | ENSMUSG00000031004 | M200001580 |
| 875 | 0.287 | Fkbp1b | FK506 binding protein 1b Gene | ENSMUSG00000020635 | M200003910 |
| 876 | 0.285 | Myod1 | myogenic differentiation 1 Gene | ENSMUSG00000009471 | M200000620 |
| 877 | 0.284 | Hap1 | huntingtin-associated protein 1 Gene | ENSMUSG00000006930 | M200002975 |
| 878 | 0.284 | Lphn1 | latrophilin 1 Gene | ENSMUSG00000013033 | M300001207 |
| 879 | 0.283 | Blvrb | biliverdin reductase B (flavin reductase (NADPH)) Gene | ENSMUSG00000040466 | M400011885 |
| 880 | 0.282 | AC166102.2 | Hematological and neurological expressed 1-like protein (HN1-like protein) | ENSMUSG00000024165 | M400000721 |
| 881 | 0.280 | Gsto1 | glutathione S-transferase omega 1 Gene | ENSMUSG00000025068 | M200000102 |
| 882 | 0.280 | Sulf2 | sulfatase 2 Gene | ENSMUSG00000006800 | M300000855 |
| 883 | 0.280 | Timp2 |  | ENSMUSG00000017466 | M300001586 |
| 884 | 0.279 | Hmgn2 | high mobility group nucleosomal binding domain 2 Gene | ENSMUSG00000003038 | M400000066 |
| 885 | 0.278 | Ndufc2 | NADH dehydrogenase (ubiquinone) 1, subcomplex unknown, 2 Gene | ENSMUSG00000030647 | M400001304 |
| 886 | 0.277 | Lmo3 | LIM domain only 3 Gene | ENSMUSG00000030226 | M400001240 |
| 887 | 0.276 | Panx1 | pannexin 1 Gene | ENSMUSG00000031934 | M200009383 |
| 888 | 0.275 | 6330527O06Rik | RIKEN cDNA 6330527O06 gene Gene | ENSMUSG00000027270 | M400011677 |
| 889 | 0.274 | Gpc1 | glypican 1 Gene | ENSMUSG00000034220 | M400001663 |
| 890 | 0.271 | Shroom2 | shroom family member 2 Gene | ENSMUSG00000045180 | M300016154 |
| 891 | 0.271 | Pftk1 | PFTAIRE protein kinase 1 Gene | ENSMUSG00000028926 | M200002365 |
| 892 | 0.270 | Hmgb2 | high mobility group box 2 Gene | ENSMUSG00000054717 | M400012694 |
| 893 | 0.270 | Ifi203 | interferon activated gene 203 Gene | ENSMUSG00000039997 | M300005163 |
| 894 | 0.269 | Zdhhc12 | zinc finger, DHHC domain containing 12 Gene | ENSMUSG00000015335 | M300001402 |
| 895 | 0.268 | Tubb5 | tubulin, beta 5 Gene | ENSMUSG00000001525 | M300000203 |
| 896 | 0.268 | Arhgdia | Rho GDP dissociation inhibitor (GDI) alpha Gene | ENSMUSG00000025132 | M200016277 |
| 897 | 0.267 | Ptn | pleiotrophin Gene | ENSMUSG00000029838 | M200001169 |
| 898 | 0.267 | Ddah2 | dimethylarginine dimethylaminohydrolase 2 Gene | ENSMUSG00000007039 | M200000598 |
| 899 | 0.267 | Tusc3 | tumor suppressor candidate 3 Gene | ENSMUSG00000039530 | M200015414 |
| 900 | 0.267 | Hsbp1 | heat shock factor binding protein 1 Gene | ENSMUSG00000031839 | M300007929 |
| 901 | 0.266 | Serpine2 | serine (or cysteine) peptidase inhibitor, clade E, member 2 Gene | ENSMUSG00000026249 | M200001184 |
| 902 | 0.265 | CT009486.7 | Ferritin light chain 1 (Ferritin L subunit 1) | ENSMUSG00000062382 | M400006088 |
| 903 | 0.262 | Mycl1 | v-myc myelocytomatosis viral oncogene homolog 1, lung carcinoma derived (avian) Gene | ENSMUSG00000028654 | M200000384 |
| 904 | 0.262 | Rnd2 | Rho family GTPase 2 Gene | ENSMUSG00000001313 | M300000179 |
| 905 | 0.262 | Nola3 | nucleolar protein family A, member 3 Gene | ENSMUSG00000027133 | M200005732 |
| 906 | 0.261 | Nsg2 | neuron specific gene family member 2 Gene | ENSMUSG00000020297 | M300002118 |
| 907 | 0.259 | Tspan6 | tetraspanin 6 Gene | ENSMUSG00000067377 | M400011324 |
| 908 | 0.259 | Hes5 | hairy and enhancer of split 5 (Drosophila) Gene | ENSMUSG00000048001 | M300018827 |
| 909 | 0.258 | Calm3 | calmodulin 3 Gene | ENSMUSG00000019370 | M300001785 |
| 910 | 0.258 | Gng12 | guanine nucleotide binding protein (G protein), gamma 12 Gene | ENSMUSG00000036402 | M200013566 |
| 911 | 0.257 | Marcks | myristoylated alanine rich protein kinase C substrate Gene | ENSMUSG00000069662 | M400012790 |
| 912 | 0.256 | AC159001.2 |  | ENSMUSG00000058777 | M400008454 |
| 913 | 0.255 | Tex14 | testis expressed gene 14 Gene | ENSMUSG00000010342 | M200008499 |
| 914 | 0.253 | Fads2 | fatty acid desaturase 2 Gene | ENSMUSG00000024665 | M200008136 |
| 915 | 0.253 | Bid | BH3 interacting domain death agonist Gene | ENSMUSG00000004446 | M200007560 |
| 916 | 0.252 | Hn1 | hematological and neurological expressed sequence 1 Gene | ENSMUSG00000020737 | M200000693 |
| 917 | 0.252 | Pfdn4 | prefoldin 4 Gene | ENSMUSG00000052033 | M400003978 |
| 918 | 0.250 | Apcdd1 | adenomatosis polyposis coli down-regulated 1 Gene | ENSMUSG00000071847 | M300021469 |
| 919 | 0.249 | Hint1 | histidine triad nucleotide binding protein 1 Gene | ENSMUSG00000020267 | M200000158 |
| 920 | 0.248 | Fgf13 | fibroblast growth factor 13 Gene | ENSMUSG00000031137 | M200002625 |
| 921 | 0.244 | Atp5k | ATP synthase, H+ transporting, mitochondrial F1F0 complex, subunit e Gene | ENSMUSG00000050856 | M400010735 |
| 922 | 0.243 | AC117232.3 | 40S ribosomal protein S26 | ENSMUSG00000025362 | M400000825 |
| 923 | 0.243 | Rras2 | related RAS viral (r-ras) oncogene homolog 2 Gene | ENSMUSG00000055723 | M400005273 |
| 924 | 0.242 | Fzd2 | frizzled homolog 2 (Drosophila) Gene | ENSMUSG00000050288 | M200016293 |
| 925 | 0.241 | Ccnd2 | cyclin D2 Gene | ENSMUSG00000000184 | M200001209 |
| 926 | 0.239 | Rps20 | ribosomal protein S20 Gene | ENSMUSG00000028234 | M400011542 |
| 927 | 0.238 | 8030462N17Rik | RIKEN cDNA 8030462N17 gene Gene | ENSMUSG00000047466 | M400012487 |
| 928 | 0.238 | Tcf7l2 | transcription factor 7-like 2, T-cell specific, HMG-box Gene | ENSMUSG00000024985 | M400009143 |
| 929 | 0.237 | Prc1 | protein regulator of cytokinesis 1 Gene | ENSMUSG00000038943 | M400009510 |
| 930 | 0.236 | Gng2 | guanine nucleotide binding protein (G protein), gamma 2 Gene | ENSMUSG00000043004 | M300014088 |
| 931 | 0.234 | Cst3 | cystatin C Gene | ENSMUSG00000027447 | M200001658 |
| 932 | 0.233 | 6720460F02Rik | RIKEN cDNA 6720460F02 gene Gene | ENSMUSG00000020808 | M200004319 |
| 933 | 0.233 | Id3 | inhibitor of DNA binding 3 Gene | ENSMUSG00000007872 | M200000033 |
| 934 | 0.233 | Hoxb6 | homeo box B6 Gene | ENSMUSG00000000690 | M200000081 |
| 935 | 0.233 | Tubb2b | tubulin, beta 2b Gene | ENSMUSG00000045136 | M200012963 |
| 936 | 0.232 | Crabp1 | cellular retinoic acid binding protein I Gene | ENSMUSG00000032291 | M200007659 |
| 937 | 0.227 | Cenpj | centromere protein J Gene | ENSMUSG00000064128 | M300012555 |
| 938 | 0.222 | Spc25 | SPC25, NDC80 kinetochore complex component, homolog (S. cerevisiae) Gene | ENSMUSG00000005233 | M300000653 |
| 939 | 0.221 | Pld3 | phospholipase D family, member 3 Gene | ENSMUSG00000003363 | M200002371 |
| 940 | 0.221 | Tpbg | trophoblast glycoprotein Gene | ENSMUSG00000035274 | M300009824 |
| 941 | 0.220 | Npnt | nephronectin Gene | ENSMUSG00000040998 | M400014702 |
| 942 | 0.220 | Top2a | topoisomerase (DNA) II alpha Gene | ENSMUSG00000020914 | M200001651 |
| 943 | 0.218 | Ckap2l | cytoskeleton associated protein 2-like Gene | ENSMUSG00000048327 | M200014321 |
| 944 | 0.217 | Sh3gl3 | SH3-domain GRB2-like 3 Gene | ENSMUSG00000030638 | M300007341 |
| 945 | 0.217 | Tuba1a | tubulin, alpha 1A Gene | ENSMUSG00000072235 | M400011156 |
| 946 | 0.216 | Dbn1 | drebrin 1 Gene | ENSMUSG00000034675 | M200003719 |
| 947 | 0.214 | Idh1 | isocitrate dehydrogenase 1 (NADP+), soluble Gene | ENSMUSG00000025950 | M200002837 |
| 948 | 0.213 | Rprm | reprimo, TP53 dependent G2 arrest mediator candidate Gene | ENSMUSG00000075334 | M400011438 |
| 949 | 0.213 | Tmeff1 | transmembrane protein with EGF-like and two follistatin-like domains 1 Gene | ENSMUSG00000028347 | M200013742 |
| 950 | 0.210 | Ly6h | lymphocyte antigen 6 complex, locus H Gene | ENSMUSG00000022577 | M300003390 |
| 951 | 0.208 | Maged2 | melanoma antigen, family D, 2 Gene | ENSMUSG00000025268 | M200004435 |
| 952 | 0.205 | Sfrp2 | secreted frizzled-related protein 2 Gene | ENSMUSG00000027996 | M400010896 |
| 953 | 0.203 | Cdc2a | cell division cycle 2 homolog A (S. pombe) Gene | ENSMUSG00000019942 | M300001903 |
| 954 | 0.201 | Nkd1 | naked cuticle 1 homolog (Drosophila) Gene | ENSMUSG00000031661 | M200006903 |
| 955 | 0.197 | Rps5 | ribosomal protein S5 Gene | ENSMUSG00000012848 | M200002233 |
| 956 | 0.192 | Pbk | PDZ binding kinase Gene | ENSMUSG00000022033 | M300003073 |
| 957 | 0.189 | Hmgb3 | high mobility group box 3 Gene | ENSMUSG00000015217 | M300001378 |
| 958 | 0.189 | Ptpro | protein tyrosine phosphatase, non-receptor type 15 Gene | ENSMUSG00000030223 | M300007079 |
| 959 | 0.187 | Grem2 | gremlin 2 homolog, cysteine knot superfamily (Xenopus laevis) Gene | ENSMUSG00000050069 | M200005192 |
| 960 | 0.187 | Pcsk9 | proprotein convertase subtilisin/kexin type 9 Gene | ENSMUSG00000044254 | M300015282 |
| 961 | 0.185 | Rab3b | RAB3B, member RAS oncogene family Gene | ENSMUSG00000003411 | M200016300 |
| 962 | 0.184 | Cd9 | CD9 antigen Gene | ENSMUSG00000030342 | M200001129 |
| 963 | 0.175 | Nnat | neuronatin Gene | ENSMUSG00000067786 | M400012513 |
| 964 | 0.172 | Slc17a6 | solute carrier family 17 (sodium-dependent inorganic phosphate cotransporter), member 6 Gene | ENSMUSG00000030500 | M200015295 |
| 965 | 0.171 | Vash2 | vasohibin 2 Gene | ENSMUSG00000037568 | M300011001 |
| 966 | 0.167 | Dynll1 | dynein light chain LC8-type 1 Gene | ENSMUSG00000009013 | M400011327 |
| 967 | 0.158 | 3110035E14Rik | RIKEN cDNA 3110035E14 gene Gene | ENSMUSG00000067879 | M400012474 |
| 968 | 0.153 | 6330403K07Rik | RIKEN cDNA 6330403K07 gene Gene | ENSMUSG00000018451 | M300001680 |
| 969 | 0.150 | Tmsb4x | thymosin, beta 4, X chromosome Gene | ENSMUSG00000049775 | M200009414 |
| 970 | 0.149 | Ddc | dopa decarboxylase Gene | ENSMUSG00000020182 | M200003183 |
| 971 | 0.145 | Tubb2a | tubulin, beta 2a Gene | ENSMUSG00000058672 | M400010936 |
| 972 | 0.140 | Cdh13 | cadherin 13 Gene | ENSMUSG00000031841 | M200004974 |
| 973 | 0.134 | Zcchc12 | zinc finger, CCHC domain containing 12 Gene | ENSMUSG00000036699 | M200013665 |
| 974 | 0.132 | Sox4 | SRY-box containing gene 4 Gene | ENSMUSG00000076431 | M400010912 |
| 975 | 0.117 | Stx1a | syntaxin 1A (brain) Gene | ENSMUSG00000007207 | M200002322 |
| 976 | 0.116 | Dpysl3 | dihydropyrimidinase-like 3 Gene | ENSMUSG00000024501 | M400014765 |
| 977 | 0.115 | Arhgdig | Rho GDP dissociation inhibitor (GDI) gamma Gene | ENSMUSG00000073433 | M300003965 |
| 978 | 0.112 | Dcx | doublecortin Gene | ENSMUSG00000031285 | M200003157 |
| 979 | 0.105 | Basp1 | brain abundant, membrane attached signal protein 1 Gene | ENSMUSG00000045763 | M300016694 |
| 980 | 0.105 | Mfap4 | microfibrillar-associated protein 4 Gene | ENSMUSG00000042436 | M200006465 |
| 981 | 0.086 | Ctxn1 | cortexin 1 Gene | ENSMUSG00000048644 | M300019424 |
| 982 | 0.079 | Efcbp2 | N-terminal EF-hand calcium binding protein 2 Gene | ENSMUSG00000031837 | M200009009 |
| 983 | 0.071 | Marcksl1 | MARCKS-like 1 Gene | ENSMUSG00000047945 | M300018771 |
| 984 | 0.050 | Gap43 | growth associated protein 43 Gene | ENSMUSG00000047261 | M300018113 |
