# Supplementary material for: Nos2 Inactivation Promotes the Development of Medulloblastoma in Ptch1+/− Mice by Deregulation of Gap43–Dependent Granule Cell Precursor Migration
Source: PLoS Genet. 2012 Mar 15;8(3):e1002572. doi: 10.1371/journal.pgen.1002572 (PMC3305407; doi:10.1371/journal.pgen.1002572)
Supplement: Table S5 — Differentially expressed genes in P9 cerebella of Ptch1+/− Nos2+/+ against wild-type mice. (DOC) [file pgen.1002572.s012.doc]

**Table S5:** Differentially expressed genes in P9 cerebella of *Ptch1^+/-^* *Nos2^+/+^* against wildtype mice.

|  | **Fold Change** | **Symbol** | **Description** | **Ensembl ID** | **Oligo ID** |
| --- | --- | --- | --- | --- | --- |
| 1 | 3.694 | Stmn1 | Dlgap1 | stathmin 1 Gene | discs, large (Drosophila) homolog-associated protein 1 Gene | ENSMUSG00000028832 ENSMUSG00000003279 | M300006321 |
| 2 | 2.045 | Hyou1 | hypoxia up-regulated 1 Gene | ENSMUSG00000032115 | M200008813 |
| 3 | 2.013 | Sorl1 | sortilin-related receptor, LDLR class A repeats-containing Gene | ENSMUSG00000049313 | M400003529 |
| 4 | 0.475 | Mctp1 | multiple C2 domains, transmembrane 1 Gene | ENSMUSG00000021596 | M200011487 |
| 5 | 0.360 | Agxt2l1 | alanine-glyoxylate aminotransferase 2-like 1 Gene | ENSMUSG00000019232 | M200006336 |
