# Supplementary material for: Nos2 Inactivation Promotes the Development of Medulloblastoma in Ptch1+/− Mice by Deregulation of Gap43–Dependent Granule Cell Precursor Migration
Source: PLoS Genet. 2012 Mar 15;8(3):e1002572. doi: 10.1371/journal.pgen.1002572 (PMC3305407; doi:10.1371/journal.pgen.1002572)
Supplement: Table S6 — Differentially expressed genes in P9 cerebella of Ptch1+/− Nos2−/− against wild-type mice. (DOC) [file pgen.1002572.s013.doc]

**Table S6:** Differentially expressed genes in P9 cerebella of *Ptch1^+/-^* *Nos2^-/-^* against wildtype mice.

|  | **Fold Change** | **Symbol** | **Description** | **Ensembl ID** | **Oligo ID** |
| --- | --- | --- | --- | --- | --- |
| 1 | 13.371 | Stmn1 | Dlgap1 | stathmin 1 Gene | discs, large (Drosophila) homolog-associated protein 1 Gene | ENSMUSG00000028832 | ENSMUSG00000003279 | M300006321 |
| 2 | 4.042 | Pomc | pro-opiomelanocortin-alpha Gene | ENSMUSG00000020660 | M200004240 |
| 3 | 3.008 | Aldh2 | aldehyde dehydrogenase 2, mitochondrial Gene | ENSMUSG00000029455 | M200000997 |
| 4 | 2.442 | Cit | citron Gene | ENSMUSG00000029516 | M400016033 |
| 5 | 2.325 | Uchl1 | ubiquitin carboxy-terminal hydrolase L1 Gene | ENSMUSG00000029223 | M300006559 |
| 6 | 2.181 | Ptch2 | patched homolog 2 Gene | ENSMUSG00000028681 | M200006050 |
| 7 | 2.167 | Rusc2 | RUN and SH3 domain containing 2 Gene | ENSMUSG00000035969 | M300010178 |
| 8 | 2.108 | Scn3a | sodium channel, voltage-gated, type III, alpha Gene | ENSMUSG00000057182 | M400000982 |
| 9 | 2.099 | 2610024B07Rik | RIKEN cDNA 2610024B07 gene Gene | ENSMUSG00000055593 | M400005234 |
| 10 | 2.099 | BC007180 | BC007180 protein Fragment | ENSMUSG00000078161 | M400004944 |
| 11 | 2.079 | AC119876.9 | Peroxisome assembly factor 1 (PAF-1)(Peroxin-2)(Peroxisomal membrane protein 3) | ENSMUSG00000040374 | M300012630 |
| 12 | 2.061 | Nova1 | neuro-oncological ventral antigen 1 Gene | ENSMUSG00000053548 | M400004507 |
| 13 | 2.045 | Spsb2 | splA/ryanodine receptor domain and SOCS box containing 2 Gene | ENSMUSG00000038451 | M200000999 |
| 14 | 2.004 | Rutbc2 | small G protein signaling modulator 1 Gene | ENSMUSG00000042216 | M300013570 |
| 15 | 0.500 | Blvrb | biliverdin reductase B (flavin reductase (NADPH)) Gene | ENSMUSG00000040466 | M400011885 |
| 16 | 0.496 | Skp1a | S-phase kinase-associated protein 1A Gene | ENSMUSG00000036309 | M300010327 |
| 17 | 0.495 | 0610040J01Rik | RIKEN cDNA 0610040J01 gene Gene | ENSMUSG00000060512 | M200008012 |
| 18 | 0.490 | Hmgn2 | high mobility group nucleosomal binding domain 2 Gene | ENSMUSG00000003038 | M400000066 |
| 19 | 0.487 | Ddt | D-dopachrome tautomerase Gene | ENSMUSG00000001666 | M200002268 |
| 20 | 0.483 | Cntn2 | contactin 2 Gene | ENSMUSG00000053024 | M400004317 |
| 21 | 0.481 | Syngr1 | synaptogyrin 1 Gene | ENSMUSG00000022415 | M300003269 |
| 22 | 0.480 | Tom1l2 | target of myb1-like 2 (chicken) Gene | ENSMUSG00000000538 | M400012183 |
| 23 | 0.478 | Lypla2 | lysophospholipase 2 Gene | ENSMUSG00000028670 | M200007536 |
| 24 | 0.468 | Zfp219 | zinc finger protein 219 Gene | ENSMUSG00000049295 | M400003527 |
| 25 | 0.424 | Agpat1 | 1-acylglycerol-3-phosphate O-acyltransferase 1 (lysophosphatidic acid acyltransferase, alpha) Gene | ENSMUSG00000034254 | M200002732 |
| 26 | 0.421 | Hcn2 | hyperpolarization-activated, cyclic nucleotide-gated K+ 2 Gene | ENSMUSG00000020331 | M200016228 |
| 27 | 0.371 | Gpc1 | glypican 1 Gene | ENSMUSG00000034220 | M400001663 |
| 28 | 0.361 | Lphn1 | latrophilin 1 Gene | ENSMUSG00000013033 | M300001207 |
| 29 | 0.335 | Dlg4 | discs, large homolog 4 (Drosophila) Gene | ENSMUSG00000020886 | M400010772 |
| 30 | 0.335 | Pcdhgc4 | protocadherin gamma subfamily A, 11 Gene | ENSMUSG00000023036 | M400009080 |
| 31 | 0.189 | 8030462N17Rik | RIKEN cDNA 8030462N17 gene Gene | ENSMUSG00000047466 | M400012487 |
| 32 | 0.042 | Gap43 | growth associated protein 43 Gene | ENSMUSG00000047261 | M300018113 |
