# Supplementary material for: Nos2 Inactivation Promotes the Development of Medulloblastoma in Ptch1+/− Mice by Deregulation of Gap43–Dependent Granule Cell Precursor Migration
Source: PLoS Genet. 2012 Mar 15;8(3):e1002572. doi: 10.1371/journal.pgen.1002572 (PMC3305407; doi:10.1371/journal.pgen.1002572)
Supplement: Table S7 — Housekeeping genes used for qRT-PCR analyses. (DOC) [file pgen.1002572.s014.doc]

**Table S7:** Housekeeping genes used for qRT-PCR analyses.

|  | **Used in** | | | |
| --- | --- | --- | --- | --- |
| **Housekeeping gene** | **Tissue**  **(Shh genes)** | **Tissue**  **(MB candidate genes)** | **C17.2 cells** | **D458 cells** |
| ***Mrpl32*** (mitochondrial ribosomal protein L32) [Mus musculus] | X |  |  |  |
| ***Anxa2*** (annexin A2) [Mus musculus] |  | X | X |  |
| ***Lmna*** (lamin A) [Mus musculus] |  | X | X |  |
| ***Tjp1*** (tight junction protein 1) [*Mus musculus*] |  | X |  |  |
| ***Ldha*** (lactate dehydrogenase A) [*Mus musculus*] |  | X | X |  |
| ***Mrps15*** (mitochondrial ribosomal protein S15) [*Mus musculus*] |  | X | X |  |
| ***LMNA*** (lamin A) [Homo sapiens] |  |  |  | X |
| ***TJP1*** (tight junction protein 1) [*Homo sapiens*] |  |  |  | X |
| ***ARF1*** (ADP-ribosylation factor 1) [*Homo sapiens*] |  |  |  | X |
| ***DCTN2*** (dynactin 2) [Homo sapiens] |  |  |  | X |

Shh, Sonic hedgehog; MB, medulloblastoma
