# Supplementary material for: Nos2 Inactivation Promotes the Development of Medulloblastoma in Ptch1+/− Mice by Deregulation of Gap43–Dependent Granule Cell Precursor Migration
Source: PLoS Genet. 2012 Mar 15;8(3):e1002572. doi: 10.1371/journal.pgen.1002572 (PMC3305407; doi:10.1371/journal.pgen.1002572)
Supplement: Table S8 — Primer sequences used for qRT-PCR analyses. (DOC) [file pgen.1002572.s015.doc]

**Table S8:** Primer sequences used for qRT-PCR analyses.

| **Gene** | **Sequence *forward* primer (5'🡪3')** | **Sequence *reverse* primer (5'🡪3')** |
| --- | --- | --- |
| ***Anxa2*** | TGGCAAGTCCCTGTACTA | TGCTGAGCCCTTCAGTCAT |
| ***Gap43*** | AGGAGGAGAAAGACGCTGTA | GGCAACGTGGAAAGCCATT |
| ***Ldha*** | GAAGGACTTGGCGGATGAG | CCGCGGTGATAATGACCAG |
| ***Lmna*** | CAGCAGTCTCGAATCCGCA | AGGTCACGCAGCTTTGCCT |
| ***Mrps15*** | CTGTCAGGATCCGCAATTATG | ACATCATAGTTGGTCTGACGG |
| ***Otx1*** | AAGACAAGCCACTCCGACAA | GCGAAGTCCTCCAAGCTGTT |
| ***Ptch1*** | TTCCTCCTGAAACCCAAAGC | CATAAAGGCTGACCCCCAG |
| ***Stmn1*** | CCCGTCCTAAATATCCAAAGAC | ACATCTCACGGTCTGGATCTA |
| ***Tjp1*** | CCATCACTCAGAGCCTGCT | CTGGATCATAACGTTTCTCACT |
| ***ANXA2*** | TCAGCATTTGGGGACGCTC | GAACAGTAGACATTTTGAAGGAAG |
| ***ARF1*** | GACCACGATCCTCTACAAGC | TCCCACACAGTGAAGCTGATG |
| ***DCTN2*** | CGCCATGGCGGACCCTAAAT | TTGTCAGCTCCTCCGCATCGAA |
| ***GAP43*** | CTGAAGAGAACATAGAAGCTG | TTCTTTACCCTCGTCCTGCC |
| ***LDHA*** | GTTCCAAGTCCAATATGGCAAC | GTTCATCTGCCAAGTCCTTCA |
| ***LMNA*** | GAGGCTCTGCTGAACTCCAA | ATCCTGAAGTTGCTTCTTGGC |
| ***MRPS15*** | GCCCACAAACGCTATCTGCT | CCTGGAAAACCCGAATGCAC |
| ***TJP1*** | CAGCAATGGAGGAAACAGCTA | AATGAGGATTATCTCGTCCACC |
| ***Ptch1*** (wildtype specific) | GGCAGCTAATCTCGAGACCA | GCCTCTTCTCCTATCTTCTGACG |
| ***Gli1*** | GACCATGCGCAGACACACGG | CGTGTGCGACCGAAGGTGCG |
| ***N-myc*** | CCGGAGAGGATACCTTGAGCG | TCTTGGGACGCACAGTGATC |
| ***Mrpl32*** | ACTACCATGGCCGGTGCGTC | ATGGATGGTCTCGAGACCA |
