# Supplementary material for: Nos2 Inactivation Promotes the Development of Medulloblastoma in Ptch1+/− Mice by Deregulation of Gap43–Dependent Granule Cell Precursor Migration
Source: PLoS Genet. 2012 Mar 15;8(3):e1002572. doi: 10.1371/journal.pgen.1002572 (PMC3305407; doi:10.1371/journal.pgen.1002572)
Supplement: Table S9 — Primer sequences used for duplex-PCR analyses and genotyping. (DOC) [file pgen.1002572.s016.doc]

**Table S9:** Primer sequences used for duplex-PCR analyses and genotyping.

| **Gene** | **Sequence *forward* primer (5'🡪3')** | **Sequence *reverse* primer (5'🡪3')** |
| --- | --- | --- |
| ***P19^ARF^*** | GCACCGGAATCCTGGAC | CGGATGCACAGAAGCACGCG |
| ***P16^INK4A^*** | GGCACTGCTGGAAGCCGG | CCGACTGCAGATGGGAC |
| ***Ptch1wt duplex*** | GGCAGCTAATCTCGAGACCA | GGCTTCTCGTTGGCTACAAGG |
| ***N-Myc*** | CGGAGAGGATACCTTGAGC | GGAGGCTGGTGAACAGAAG |
| ***Nkx2.2*** | CGACAGCAGCGACAACC | GGTTACTGCCTCAGCAATGG |
| ***E.coli lacZ*** | CGGAAAGCTGGCTGGAGTG | GCGTCTGGCCTTCCTGTAG |
| ***Nos2 exon12*** | CCCCAACTCTCCTTCCTTTC | GGGACACAGCACAGACAGAG |
| ***Nos2 exon11_neo*** | GCATCTCAGTCCACTCCATC | GAGCAGCCGATTGTCTGTTG |
| ***Tp53 exon 4.1*** | GGTGTTGGGCTGGTAGGCTG | CCATGGAGTGGCTGGGGCAG |
| ***Tp53 exon 4.2*** | CTGCAGCACAGGACCCTGTC | CACACGAAAGACAACTCCCCGG |
| ***Tp53 exon 5*** | CCGACCTCCGTTCTCTCTCC | CCACAGGCGGTGTTGAGGGC |
| ***Tp53 exon 6*** | GGCTTCTGACTTATTCTTGC | CAACTGTCTCTAAGACGCAC |
| ***Tp53 exon 7*** | GCTGCAGGTCACCTGTAGTG | GCTAACCTAACCTACCACGC |
| ***Tp53 exon 8*** | CTGGTCCTTTTCTTGTCCCG | GGGTGAAGCTCAACAGGCTC |
| ***Tp53 exon 9*** | CACCTCTTGCTCTCTCCTTC | CCTGGCAACCTGCTAATAAC |
| ***Tp53 exon 10*** | GTGGTTGTGTGACCTTGTCC | GTCTGGGTAGAGCACCACAG |
