# Supplementary material for: Nos2 Inactivation Promotes the Development of Medulloblastoma in Ptch1+/− Mice by Deregulation of Gap43–Dependent Granule Cell Precursor Migration
Source: PLoS Genet. 2012 Mar 15;8(3):e1002572. doi: 10.1371/journal.pgen.1002572 (PMC3305407; doi:10.1371/journal.pgen.1002572)
Supplement: Text S1 — Supporting Results. (DOC) [file pgen.1002572.s017.doc]

# Supporting Results

## Targeted molecular analyses of selected genes

Exons 4 to 10 of the *Tp53* gene were screened for mutations by single-strand conformation polymorphism (SSCP) analysis as reported . Gene copy numbers of the *Ptch1,* *p16^INK4a^, p19^ARF^* and *N-myc* genes were assessed by duplex PCR analysis using specific primers for each gene and *Nkx2.2* as reference gene. An increased target/reference gene ratio of more than threefold of the ratio obtained for constitutional DNA was considered as evidence for gene amplification. A reduced target/reference gene ratio of less than 0.3-fold relative to the ratio obtained for constitutional DNA was considered as evidence for homozygous gene deletion. Table S1 summarizes the results obtained by the targeted molecular analyses. *Tp53* mutations were not detected in the investigated 21 medulloblastomas (8 *Ptch1^+/-^Nos2^+/+^* and 13 *Ptch1^+/-^ Nos2^-/-^*). A deletion of the wildtype *Ptch1* allele was found in 11 of 21 tumors. Losses of the *p16^INK4a^* locus were not identified, but two medulloblastomas showed deletions affecting *p19^ARF^*, with one tumor demonstrating evidence of homozygous *p19^ARF^* loss. Amplification of the *N-myc* gene was not detectable (for primer sequences see Table S9).

## Assessment of stromal marker expression

Since possibly involved non-neoplastic cells, like pericytes, vascular endothelial cells or microglia, are presumably present in low amounts and thus undergo a high dilution of their specific expression signatures within profiles of entire tissues, a set of specific marker genes for each of these cell types was separately assessed for transcript abundance. As a result, 14 out of 16 markers displayed a low to moderate signal intensity on the array with no difference being detectable between genotypes (Table S3). Nevertheless, reduction of microglia activity due to impaired NO production as a minor effect on tumor progression cannot be excluded.

## Identification of housekeeping genes

In order to identify constitutively expressed genes suitable for normalization of qRT-PCR measurements, microarray profiles of all samples were assessed for features that show low overall variance and a concurrent median to high intensity (quantile rank). Stably measurable corresponding transcripts were subsequently selected as housekeeping genes (HKGs) for murine cerebellum and partially complemented with literature-derived HKGs for the measurements in the human medulloblastoma cell line (Table S7).

## N-myc expression in medulloblastoma samples

According to previous studies by Ciani *et al*., nitric oxide (NO) reduces N-myc in GCPs, thus leading to a decreased proliferation . However, expression analyses of our tumors did not show any changes of *N-myc* transcript levels in *Nos2*-deficient medulloblastomas. Nevertheless, as the effect described by Ciani *et al*. would provide a straightforward explanation for the increased tumor rate observed in *Ptch1^+/-^ Nos2^-/-^* mice, N-myc protein levels were examined by Western blot in three *Ptch1^+/-^* *Nos2^+/+^* and five *Ptch1^+/-^ Nos2^-/-^* medulloblastoma samples. The results presented in Figure S2 showed very heterogeneous signal intensities for N‑myc across all tumors, independent of the genotype. Adjustment to co-staining of the housekeeping gene α-tubulin, digital readout, and subsequent quantification via ImageJ finally yielded no significant difference of N-myc protein abundances between medulloblastomas of the two distinct genotypes.

## QRT-PCR validation of candidate genes

The comparison of expression profiles from medulloblastoma samples revealed *Otx1* (orthodenticle homolog 1), a marker for the nodular/desmoplastic variant , to be the most upregulated gene in combined *Ptch1^+/-^ Nos2^-/-^* mice versus *Ptch1^+/-^* *Nos2^+/+^* single mutants (5.66-fold). Furthermore, *Pdgfra* (platelet derived growth factor receptor alpha), which is known to be an important proto-oncogene in the pathogenesis of many CNS tumors , as well as *Stmn1* with a previously reported role in human medulloblastomas were selected as candidate genes for qRT-PCR validation. QRT-PCR measurements were conducted in an expanded medulloblastoma sample set of seven tumors per genotype. As shown in Figure S3A, *Otx1* gene expression was increased by trend in the *Ptch1^+/-^ Nos2^-/-^* genotype, although the average difference did not reach significance (p=0.071). Upregulation of *Pdgfra* in *Ptch1^+/-^ Nos2^-/-^* tumors also failed to be significant in the validation set (Figure S3B). Finally, expression of *Stmn1* in qRT-PCR did not correspond to the array data at all and displayed no consistent difference between tumors of both genotypes (Figure S3C). The significant differential expression of the three selected candidate proto-oncogenes observed in the microarray analysis could therefore not be confirmed for higher sample numbers.

## Copy number alterations in medulloblastoma samples

Analyses of array-CGH experiments indicated *Ptch1^+/-^* *Nos2^+/+^* medulloblastoma samples to have a more heterogeneous karyotype compared to the *Ptch1^+/-^ Nos2^-/-^* genotype*.* In the *Ptch1^+/-^* *Nos2^+/+^* group, gains of whole chromosomes 8 and 17 were observed in two out of five cases with an additional trisomy of chromosome 14 and 18 being also present in these samples, respectively. In contrast, six of seven *Ptch1^+/-^ Nos2^-/-^* tumors displayed exactly the same karyotype with one outlier showing an additional trisomy of chromosome 14. However, a different degree of chromosomal instability as an effect secondary to Nos2-deficiency is rather unlikely, since there was at least one *Ptch1^+/-^* *Nos2^+/+^* tumor that completely lacked any gross chromosomal aberration.

## Inhibition of nitric oxide production

Inhibition of NO synthases in cell culture using L-NAME was confirmed by photometric measurements following analytical reaction. As shown in Figure S4, detectable amounts of the NO-metabolite nitrite were considerably reduced in both cell lines 24 hours after inhibitor application. Notably, addition of the inhibitor substance *per se* increased baseline NO-measurement, possibly due to the nitro group present in the molecule itself.

## FACS analysis after L-NAME treatment

Owed to the large pattern of possible NO interactions and thereby affected physiological processes, a general reduction of cellular NO is likely to exhibit numerous effects. In order to confirm the observed downregulation of GAP43 to be a regulatory consequence of NO deprivation rather than an implication secondary to physiological changes, apoptosis and cell cycle analyses were performed. FACS measurements of Annexin V-PE stained cells indicated no difference in cell death between treatment and control (Figure S5B). FACS analyses of propidium iodide stained cells showed no alteration in cell cycle due to L-NAME administration (Figure S5A).

## Gap43 knockdown in c17.2 cells

The neuronal precursor cell line c17.2 was separately transfected with five different shRNA-expressing vector constructs (sh38-sh42, pLKO.1-puro), designed by Sigma-Aldrich to target murine *Gap43* transcripts. Furthermore, a validated non-target shRNA (shNT) and a construct targeting GFP (shGFP) were inserted into the same vector backbone to serve as negative controls. Transfection efficiency was slightly above 40 % as determined in advance by GFP expression from a pEGFP-C1 vector. Following 72 hours after initial transfection, Gap43 expression was measured on protein level using Western blot analysis. Estimation of knockdown efficiencies were done by quantification of protein bands normalized to α-tubulin levels using the Image J software. This demonstrated a minor reduction of Gap43 for sh38, sh40 and sh41 (Figure S6A-B). In contrast, the two constructs sh39 and sh42 exhibited the highest efficiencies with a decrease on protein level of 40 % and 60 %, respectively, and were therefore used for subsequent experiments.

Concerning the phenotypic implications, all shRNA constructs were found to impede migration with the degree of reduction closely matching their respective efficiency to downregulate Gap43 protein expression (Figure S6C).

## FACS analysis after Gap43 knockdown

To exclude the possibility that the knockdown-specific differences observed in migration assays are reflecting unequal proliferation, cell cycle analyses were performed. FACS measurements of propidium iodide-stained cells indicated unchanged mitosis in knockdown and control experiments, which underlined the Boyden chamber assay results to be attributable to altered migration properties (Figure S7).

1. Reifenberger J, Wolter M, Knobbe CB, Kohler B, Schonicke A, et al. (2005) Somatic mutations in the PTCH, SMOH, SUFUH and TP53 genes in sporadic basal cell carcinomas. Br J Dermatol 152: 43-51.

2. Ciani E, Severi S, Contestabile A, Bartesaghi R (2004) Nitric oxide negatively regulates proliferation and promotes neuronal differentiation through N-Myc downregulation. J Cell Sci 117: 4727-4737.

3. de Haas T, Oussoren E, Grajkowska W, Perek-Polnik M, Popovic M, et al. (2006) OTX1 and OTX2 expression correlates with the clinicopathologic classification of medulloblastomas. J Neuropathol Exp Neurol 65: 176-186.

4. Toepoel M, Joosten PH, Knobbe CB, Afink GB, Zotz RB, et al. (2008) Haplotype-specific expression of the human PDGFRA gene correlates with the risk of glioblastomas. Int J Cancer 123: 322-329.

5. Gilbertson RJ, Langdon JA, Hollander A, Hernan R, Hogg TL, et al. (2006) Mutational analysis of PDGFR-RAS/MAPK pathway activation in childhood medulloblastoma. Eur J Cancer 42: 646-649.

6. Blom T, Roselli A, Hayry V, Tynninen O, Wartiovaara K, et al. Amplification and overexpression of KIT, PDGFRA, and VEGFR2 in medulloblastomas and primitive neuroectodermal tumors. J Neurooncol 97: 217-224.

7. MacDonald TJ, Brown KM, LaFleur B, Peterson K, Lawlor C, et al. (2001) Expression profiling of medulloblastoma: PDGFRA and the RAS/MAPK pathway as therapeutic targets for metastatic disease. Nat Genet 29: 143-152.

8. Neben K, Korshunov A, Benner A, Wrobel G, Hahn M, et al. (2004) Microarray-based screening for molecular markers in medulloblastoma revealed STK15 as independent predictor for survival. Cancer Res 64: 3103-3111.

9. Kuo MF, Wang HS, Kuo QT, Shun CT, Hsu HC, et al. (2009) High expression of stathmin protein predicts a fulminant course in medulloblastoma. J Neurosurg Pediatr 4: 74-80.
