# Supplementary material for: Nos2 Inactivation Promotes the Development of Medulloblastoma in Ptch1+/− Mice by Deregulation of Gap43–Dependent Granule Cell Precursor Migration
Source: PLoS Genet. 2012 Mar 15;8(3):e1002572. doi: 10.1371/journal.pgen.1002572 (PMC3305407; doi:10.1371/journal.pgen.1002572)
Supplement: Text S2 — Supporting Methods. (DOC) [file pgen.1002572.s018.doc]

# Supporting Methods

## Housing of mice

All mice were housed in the central animal facility of Heinrich Heine University Düsseldorf at 22 °C and 55 % relative humidity. They were exposed for 12 h daily to 320 lux white light. *Ptch1^+/-^* mice were genotyped using PCR primers specific to the *lacZ* insert . The Nos2 knock-out was confirmed by PCR with primers either specific for the wild-type exon 12 or the exon 11-neo insert (for primer sequences see Table S9).

## Microarray production

A set of 35 852 lyophilized 70mer oligonucleotide probes (Mouse Genome Oligo Set Version 4.0; Operon, Cologne, Germany) were dissolved in spotting buffer (3 x SSC, 1.5 M betaine) to a final concentration of 20 µM and printed on epoxysilane-coated glass slides (Schott Nexterion, Mayence, Germany). Chip printing was conducted using a VersArray Chipwriter Pro System (BioRad, Hercules, USA) equipped with 48 946MP3 Micro Spotting Pins (Arrayit, Sunnyvale, USA). Parameters for the printing procedure were set to generate an array format that contained 36 480 spots (features) in 48 subarrays of 28 columns x 28 rows. Printed chips were baked for 60 minutes at 70 °C and stored airtight under dry condition at 4 °C until use. Prior to hybridization, slides containing spotted arrays were chemically blocked for 20 minutes at 50 °C (0.3 % ethanolamine, 0.1 % SDS, 0.1 M Tris pH 9.0), washed for 2 minutes in distilled water and dipped for 10 seconds in hot water (95 °C).

## Hybridization procedure

Purified dye-labeled sample and reference cDNA were combined for two-color hybridizations with 140 µl UltraHyb buffer (Ambion, Austin, USA) pre-warmed to 70 °C. The sample hybridization mix was incubated under rotation for 60 minutes at 60 °C and subsequently heated to 70 °C for 10 minutes. Array hybridization and washing procedures were automatically conducted in a GeneTac chamber (Genomic Solutions, Ann Arbor, USA). Hybridization conditions were set to 42 °C for 23 hours with gentle agitation. Hybridized arrays were then successively washed at 36 °C with *i)* medium stringency buffer (0.5 x SSC, 0.1 % SDS (w/v)), *ii)* high stringency buffer (0.05 x SSC, 0.1 % SDS (w/v)), and *iii)* post wash buffer (0.05 x SSC). Subsequently, the chambers were dissembled and the chips were incubated for 1 minute in post wash/Tween (0.05x SSC, 0.05% Tween-20) for 30 seconds at room temperature. Immediately thereafter, slides were dried by centrifugation.

## Readout and data preprocessing

Gene expression microarray read out was accomplished in a two-color Agilent Scanner G25505B (Agilent, Santa Clara, USA) with 5 µm resolution and automatically adjusting PMT voltages according to manufacturer’s specification. Recorded images were analyzed using GenePix Pro 6.0 software (Axon, Burlingame, USA)) and raw intensity tables were preprocessed on the in-house analysis platform ChipYard (http://www.dkfz.de/genetics/ChipYard/). This included data filtering based on *i)* mean-to-median ratio in both color channels (< 3.0), *ii)* minimal signal intensity in at least one color channel (> 250), *iii)* signal-to-background ratio in at least one color channel (> 1.5) and *iv)* manual user flag for each feature. Subsequently, variance stabilization normalization was applied and the gene-wise average signal values of color switch experiments were calculated. Annotation of oligonucleotide probes present on the gene expression array was retrieved via the PIMS© database by sequence alignment to EnsEMBL database version 54.

The Read out of CGH arrays was accomplished as described above. Recorded images were analyzed using Feature Extraction 9.1 software (Agilent). Raw intensity data tables were filtered according to *i)* mean-to-median ratio (< 3), *ii)* minimal signal intensity (> 90), and *iii)* signal-to-background ratio (> 1.5) in both color channels, respectively. For arrays showing high levels of noise, meaning an average noise bandwidth of more than 2-fold the overall calculated balanced state, the filter criteria were slightly adjusted with *i)* mean-to-median ratio lying within 1 x the interquartile range, *ii)* minimal signal intensity (> 200), and *iii)* signal-to-background ratio (> 2), again being applied to both color channels, respectively. Finally, array-wise data normalization was performed using the LOESS algorithm. Annotation of oligonucleotide probes on CGH arrays were retrieved from the PIMS database and referred to EnsEMBL database version 56.

## Statistical analyses of microarray data

Differential gene expression was assessed using the *limma* package for the R computing environment . Oligonucleotide probes were excluded from analysis if absent in more than 50 % of all individuals within one minimal group that consisted of all samples representing the same specimen conditions and genotype. Missing values for remaining probes were imputed according to the k-nearest neighbor algorithm. A probe was considered to be significantly differentially expressed for at least a 2-fold linear difference between the two groups of interest and a p-value below 0.05 calculated in a moderated t-statistic and adjusted for multiple testing according to Benjamini & Hochberg. Array features matching multiple genes or untranslated genomic regions were excluded from lists of differentially expressed genes. Values of identical probes were averaged and multiple probes detecting the same transcript were selected for the most 3’-matching one. Unsupervised hierarchical cluster analysis was performed using the *pvclust* package environment . Sample trees were generated by applying the Euclidean distance measure and the complete linkage method in 1000 repeats of bootstrap resampling. Heat map visualizations were performed with normalized log2 ratios of sample against Universal Mouse Reference (Stratagene, La Jolla, USA) using the MultiExperiment Viewer v4.0 software . For the identification of genomic aberrations from array-CGH experiments, packages of the Bioconductor project implemented in the ChipYard framework (http://www.dkfz.de/genetics/ChipYard/) were used. The GLAD algorithm was applied for segmentation and to detect breakpoints. A minimal region size of 3 oligonucleotide probes for low noise arrays and 6 oligonucleotide probes for high noise arrays was considered as true outliers, respectively. Regions with log2 ratios (to balanced region) of less than -1.25 were scored as losses and regions with log2 ratios higher than 1.25 were scored as gains. For high noise arrays, these thresholds were set to -0.45 and 0.45. Enrichment for Gli targets was determined based on a set of 420 genes that were recently identified to be Gli1-regulated in GCPs of murine cerebellum . Accordingly, sets of differentially expressed genes from the different comparisons were partitioned and subjected to a chi-square test against the total of valid probe signals (18 905) to calculate p-values.

## Western blot procedure

SDS-PAGE was conducted with 1 mm spacing and 10 % acrylamide. Samples were adjusted to equal concentrations in protein-lysis buffer (5 % SDS, 10 mM NaF, 1 mM sodium orthovanadate, 5 mM EDTA, 10 mM phenylmethylsulfonylfluorid (PMSF), 1 x Mini Protease Inhibitor Cocktail (Roche, Mannheim, Germany)) and supplemented with Laemmli buffer before being heated to 96 °C for 5 minutes. Electrophoresis was performed for 10 minutes at 65 V for sample stacking, followed by 60 minutes at 130 V and 250 mA for protein separation. Electrophoretic transfer of separated proteins from PAA (polyacrylamide) gel to PVDF (polyvinylidene fluoride) membrane was carried out in a Mini Trans-Blot wet gel transfer apparatus (BioRad). The PVDF membrane was briefly activated in methanol and then soaked in transfer buffer (25 mM Tris, pH 8.8, 200 mM glycine, and 20 % methanol) before being assembled with the PAA gel. Transfer was conducted in ice-cold transfer buffer for at ~ 150 V 50 minutes with increasing amperage (100 mA, 200 mA, 300 mA, 400 mA, and 500 mA). Subsequently, the PDVF membrane was washed in TBS-T and blocked in TBS-T/5 % whole milk powder, for 1 hour at room temperature. For protein detection, PDVF membranes were stained with primary antibody diluted in either TBS‑T/5 % whole milk powder (α-tubulin, 1:2000, Sigma-Aldrich and GAP43, 1:1000, Sigma-Aldrich) or TBS‑T/5 % BSA (N-MYC, 1:1000, Cell Signaling, Danvers, USA). Incubation with primary antibodies was performed on a rotator over night at 4 °C. Membranes were then washed in TBS-T and incubated with corresponding secondary antibody, conjugated with horseradish peroxidase, for 1 hour at room temperature (dilution: 1:10 000 in TBS‑T/5 % whole milk powder). After washing membranes in TBS‑T, the chemiluminescent substrate ECL Western Blotting Detection Reagent (Amersham, Amersham, UK) was applied according to manufacturer’s instructions. Developed X-ray films were scanned with high resolution and staining intensities were determined using the ImageJ software. Sample-wise protein amount was calculated as intensity ratio between the protein of interest and the house-keeping protein (α-tubulin).

## Cell lines and culture

For functional analyses, the neuronal progenitor cell line c17.2 (European Collection of Cell Cultures) and the medulloblastoma cell line D458 (kindly provided by Prof. Dr. Darell Bigner, Duke University, Durham) were cultivated in DMEM (Invitrogen, Carlsbad, USA) supplemented with 10 % FCS and Improved MEM Zinc Option medium (Invitrogen) supplemented with 20 % FCS, respectively.

The cell line c17.2 was originally generated from cultured neuronal progenitor cells of the EGL derived from a neonatal mouse cerebellum and immortalized by retroviral transduction of the avian *v-myc* oncogene . In engraftment experiments performed in newborn mice, cells of this lineage were demonstrated to integrate into a developing cerebellum in a non-tumorigenic fashion and to fully differentiate into granule neurons and glial cells . Although this cell line does not express detectable amounts of *Nos2* (data not shown), *Nos1*-derived NO presumably takes over the regulation of Gap43. The cell line D458 was isolated from a relapsed human MB and demonstrates basal *NOS2* expression level (data not shown).

## Nitric oxide assay

Effectiveness of Nos inhibition by L-NAME was verified in c17.2 and D458 cells by Griess reaction of nitrite (NO_2_^-^), in the presence of 1 mM sulfanilamide and 1 mM *N*-(1-naphthyl)-ethylenediamine (NED).Cells were cultivated in DMEM without phenol red to avoid interference with the colorimetric measurement. For each cell culture sample, 50 µl supernatant were separated from cell debris by ultrafiltration (YM-10 filter columns, Millipore, Billerica, USA) and the flow-through was used for the following assay procedure. First, reduction of present nitrate to nitrite was carried out in a total volume of 50 µl with 50 nU nitrate reductase (Roche), 0.5 µM NADPH, and 50 nM FAD for 15 minutes at 37 °C. Then, residual NADPH was oxidized with 11 mU lactate dehydrogenase (Roche) and 0.2 mM sodium pyruvate for 5 minutes at 37 °C. Subsequently, samples were cooled down to 4 °C and supplemented with 7 µl sulfanilamide (10 mM). Following a short incubation for 1 minute at 4 °C, 7 µl 0.1 M HCL were added and the reaction was kept at 4 °C for 5 minutes. The samples were then cleared by centrifugation and 50 µl of the supernatant was mixed with 7 ml NED (10 mM). After 10 minutes incubation at room temperature, absorbance at 540 nm was measured. For relative quantification, absorbance of samples was normalized to a water control accordingly treated in parallel (Figure S3.)

## Cell cycle analysis

The applied cell cycle analysis is based on propidium iodide (PI) intercalating into DNA, to discriminate between a haploid karyotype (G_0_ or G_1_-phase), a tetraploid karyotype (G_2‑_phase or early mitosis), or an intermediate state (S-phase). The relative amount of cells in G_0_/G_1_ or G_2_/M thus reflects differences in proliferation behavior or distinct imbalances in checkpoint control. Between 1 x 10^6^ and 2 x 10^6^ cells per cell culture sample were trypsinized, thoroughly resuspended in 2 ml PBS and subsequently flushed through to a 30 µm MACS pre-separation filter (Miltenyi Biotec, Bergisch Gladbach, Germany). Separated cells were then pelleted by centrifugation and, after discarding the supernatant, resuspended in 50 µl pre-cooled PBS (4 °C) before being transferred into a FACS tube. For fixation, 800 µl of 70 % ethanol (‑20 °C) were added to the sample in a drop-wise manner while shaking. Cells samples were incubated at -20 °C for at least 1 hour and then replenished with 2.7 ml cold PBS (4 °C). Following a further centrifugation step, the supernatant was discarded and the cell pellet was resuspended in 200 µl PBS containing 50 ng/µl PI and 200 ng/µl RNAse. Next, cell samples were incubated for 30 minutes at 37 °C in the dark and subjected to FACS measurement. The flow cytometry analyses reported here were conducted on a FACS Canto II flow cytometer (BD Bioscience, Franklin Lakes, USA) using BD FACS Diva software (BD Bioscience).

## Apoptosis assay

For measuring the amount of dying cells, Annexin V was used to mark the exposure of phosphatidylserine at the plasma membrane asan early stage of apoptosis. Furthermore, the DNA-intercalating fluorescent dye 7-aminoactinomycin (7AAD) was applied to also identify late apoptotic or necrotic cells showing disrupted membrane integrity. A total of 1 x 10^6^ - 2 x 10^6^ cells were harvested from each cell culture sample and pelleted by centrifugation. The supernatant was discarded and the cells were resuspended in 30 µl Annexin V-PE/7‑AAD/1 x Annexin binding-buffer (BD Bioscience) (10 %/10 %/80 % (v/v)). Following incubation for 15 minutes at 4 °C, 150 µl 1 x Annexin binding-buffer were added and cell samples were immediately subjected to FACS measurement.

## Preprocessing of formalin-fixed and paraffin-embedded (FFPE) sections

Sections of 4 µm thicknesses were cut from blocks of FFPE postnatal cerebellar tissue specimens using a microtome (Leica, Wetzlar, Germany). Sections were mounted on silane-coated glass slides, and dried for 24 hours at 37 °C. Deparaffinization was accomplished by successive incubation in *i)* xylol (100 %) for 5 minutes, *ii)* xylol (100 %) for 5 minutes, *iii)* ethanol (100 %) for 2 minutes, *iv)* ethanol (100 %) for 2 minutes, *v)* ethanol (96 %) for 2 minutes, *vi)* ethanol (70 %) for 2 minutes, and *vii)* distilled water for 1 minute. For antigen retrieval, sections were then incubated in a plastic cuvette filled with sodium citrate buffer (10 mM sodium citrate, pH 6.0, and 0.05 Tween-20) for 30 minutes at about 95 °C. Thereafter, the cuvette was cooled down at room temperature for 20 minutes and sodium citrate buffer was removed by dipping sections into distilled water for 1 minute. Next, sections were washed in TBS for 2 x 5 minutes followed by incubation in 10 % H_2_O_2_/TBS for 10 minutes to inactivate endogenous peroxidases. Prior to antibody incubation, endogenous biotin was blocked with Avidin D Blocking-Solution (Dako, Glostrup, Denmark) for 15 minutes. After a further washing step in TBS, residual avidin was in turn blocked with Biotin Blocking-Solution for 15 minutes.

1. Goodrich LV, Milenkovic L, Higgins KM, Scott MP (1997) Altered neural cell fates and medulloblastoma in mouse patched mutants. Science 277: 1109-1113.

2. Laubach VE, Shesely EG, Smithies O, Sherman PA (1995) Mice lacking inducible nitric oxide synthase are not resistant to lipopolysaccharide-induced death. Proc Natl Acad Sci U S A 92: 10688-10692.

3. Huber W, von Heydebreck A, Sultmann H, Poustka A, Vingron M (2002) Variance stabilization applied to microarray data calibration and to the quantification of differential expression. Bioinformatics 18 Suppl 1: S96-104.

4. Smyth GK (2004) Linear models and empirical bayes methods for assessing differential expression in microarray experiments. Stat Appl Genet Mol Biol 3: Article3.

5. Gentleman RC, Carey VJ, Bates DM, Bolstad B, Dettling M, et al. (2004) Bioconductor: open software development for computational biology and bioinformatics. Genome Biol 5: R80.

6. Shimodaira H (2002) An approximately unbiased test of phylogenetic tree selection. Syst Biol 51: 492-508.

7. Saeed AI, Sharov V, White J, Li J, Liang W, et al. (2003) TM4: a free, open-source system for microarray data management and analysis. Biotechniques 34: 374-378.

8. Hupe P, Stransky N, Thiery JP, Radvanyi F, Barillot E (2004) Analysis of array CGH data: from signal ratio to gain and loss of DNA regions. Bioinformatics 20: 3413-3422.

9. Lee EY, Ji H, Ouyang Z, Zhou B, Ma W, et al. (2010) Hedgehog pathway-regulated gene networks in cerebellum development and tumorigenesis. Proc Natl Acad Sci U S A 107: 9736-9741.

10. Ryder EF, Snyder EY, Cepko CL (1990) Establishment and characterization of multipotent neural cell lines using retrovirus vector-mediated oncogene transfer. J Neurobiol 21: 356-375.

11. Snyder EY, Deitcher DL, Walsh C, Arnold-Aldea S, Hartwieg EA, et al. (1992) Multipotent neural cell lines can engraft and participate in development of mouse cerebellum. Cell 68: 33-51.

12. He XM, Wikstrand CJ, Friedman HS, Bigner SH, Pleasure S, et al. (1991) Differentiation characteristics of newly established medulloblastoma cell lines (D384 Med, D425 Med, and D458 Med) and their transplantable xenografts. Lab Invest 64: 833-843.
